# ADAMTS8 Promotes Cardiac Fibrosis Partly Through Activating EGFR Dependent Pathway

**DOI:** 10.3389/fcvm.2022.797137

**Published:** 2022-02-10

**Authors:** Yafang Zha, Yanyan Li, Zhuowang Ge, Jian Wang, Yuheng Jiao, Jiayan Zhang, Song Zhang

**Affiliations:** ^1^Department of Cardiology, Renji Hospital, School of Medicine, Shanghai Jiao Tong University, Shanghai, China; ^2^Department of Cardiology, Xinhua Hospital, School of Medicine, Shanghai Jiao Tong University, Shanghai, China

**Keywords:** ADAMTS8, cardiac fibrosis, heart failure, myofibroblast, EGFR pathway

## Abstract

Myocardial infarction or pressure overload leads to cardiac fibrosis, the leading cause of heart failure. ADAMTS8 (A disintegrin and metalloproteinase with thrombospondin motifs 8) has been reported to be involved in many fibrosis-related diseases. However, the specific role of ADAMTS8 in cardiac fibrosis caused by myocardial infarction or pressure overload is yet unclear. The present study aimed to explore the function of ADAMTS8 in cardiac fibrosis and its underlying mechanism. ADAMTS8 expression was significantly increased in patients with dilated cardiomyopathy; its expression myocardial infarction and TAC rat models was also increased, accompanied by increased expression of α-SMA and Collagen1. Adenovirus-mediated overexpression of ADAMTS8 through cardiac *in situ* injection aggravated cardiac fibrosis and impaired cardiac function in the myocardial infarction rat model. Furthermore, *in vitro* studies revealed that ADAMTS8 promoted the activation of cardiac fibroblasts; ADAMTS8 acted as a paracrine mediator allowing for cardiomyocytes and fibroblasts to communicate indirectly. Our findings showed that ADAMTS8 could damage the mitochondrial function of cardiac fibroblasts and then activate the PI3K-Akt pathway and MAPK pathways, promoting up-regulation of YAP expression, with EGFR upstream of this pathway. This study systematically revealed the pro-fibrosis effect of ADAMTS8 in cardiac fibrosis and explored its potential role as a therapeutic target for the treatment of cardiac fibrosis and heart failure.

## Introduction

Heart failure is an increasingly concerning public health problem with alarming morbidity and mortality, and cardiac fibrosis remains the main pathological outcome of heart failure (1). The prominent features of cardiac fibrosis are excessive remodeling and accumulation of extracellular matrix (ECM) (2). Replacement fibrosis in research animal models is typically established by myocardial infarction and the reactive fibrosis is induced by the pressure load model (3). When injury or pressure overload occurs, cardiac fibroblasts differentiate into highly specialized myofibroblasts, increasing the production/secretion of fibrous collagen, stromal cell proteins, and the expression of α-smooth muscle actin (α-SMA, ACTA2 gene) to cope with injury or stress (4). Additionally, the development of cardiac fibrosis is also controlled by the myocyte-fibroblast communication (5). Appropriate fibrotic response contributes to tissue repair. However, excessive fibrosis caused by continuous activation of myofibroblasts can lead to a gradual decrease in tissue compliance, reduced nutrient and oxygen delivery, and increased myocardial atrophy and cell death, leading to progressive left ventricular dilation and dysfunction (6–8). A recent study showed that ablation of myofibroblasts under chronic stress has cardioprotective effects; eliminating the stress stimulus after activation of myofibroblasts permitted these cells to restore to a quiescent state, indicating that myofibroblasts could be a potential target to reduce fibrosis and lessen the severity of disease (9). Therefore, the present study investigated the molecular mechanism of the activation and maintenance of cardiac fibroblasts to find a new method for heart failure treatment.

ADAMTS family proteins (ADAMTS) are similar in structure and function to metalloproteinases (ADAM) (10). The main difference is that ADAM is a membrane-anchored protein, while ADAMTS is a secreted protease that binds to the extracellular matrix (ECM) (11). Recent reports indicated that certain ADAMTSs play an important role in regulating cell proliferation, adhesion, migration and intracellular signal transduction (10, 11). In the transverse aortic constriction (TAC) model, the ADAMTS16 expression level was increased and its overexpression *in vitro* activated fibroblasts (12). In the pressure overload mouse model, ADAMTS2 was upregulated during cardiac hypertrophy (13). Many studies have described the important role of ADAMTSs in cardiac fibrosis and heart failure, specifically ADAMTS8 playing a key role in cardiovascular disease (14). ADAMTS8 promoted the development of pulmonary arterial hypertension (PH) and right ventricular failure and the proliferation of PASMCs, ECM remodeling, and endothelial dysfunction in an autocrine/paracrine manner (15). Besides, Badshah et al. discovered that ADAMTS8 was upregulated in linear morphoea and normal fibroblasts transfected with ADAMTS8 overexpression plasmid differentiated into myofibroblasts (16). However, the effect of ADAMTS8 in myocardial fibrosis has not been thoroughly investigated. Therefore, this study aimed to explore the effect and mechanism of ADAMTS8 in myocardial fibrosis after injury or stress.

## Materials and Methods

### Human Heart Tissue

Heart tissue with severe fibrosis was collected from patients with DCM who underwent surgery at Xinhua Hospital. Normal heart tissue was derived from donors who died of brain death. After obtaining the patient's left ventricular tissue, one part was used for protein extraction, and the other part was fixed for immunofluorescence staining.

### Animal Studies

Male Sprague-Dawley rats used for the study were purchased from Jihui Laboratory Animal Breeding Co., Ltd. (Shanghai, China). The myocardial infarction (MI) model was established in rats *via* permanent ligation of the left anterior descending branch of the coronary artery as previously described (17). After administrating anesthesia by intraperitoneal injection of 3% sodium pentobarbital at 50 mg/kg of rat body weight, the rats were fixed on a pad, and the chest was shaved and disinfected. The rats were then intubated and ventilated with a positive pressure artificial respirator (tidal volume 7–8 mL, respiratory rate 80 breaths/min). The thoracotomy was performed on the third and fourth intercostal spaces on the left side of the rat sternum. Then, the left anterior descending coronary artery was sutured with silk sutures 2–3 mm below the root of the left atrial appendage. The anterior wall of the heart immediately turned pale after ligation, proving that the myocardial infarction model was successfully established. Additionally, the pressure overload rat model was induced by TAC as described previously (18). Briefly, Sprague-Dawley rats were anesthetized with pentobarbital and placed on a ventilator. A suprasternal incision was made to expose the aortic root, and a tantalum clip with an ID of 0.58 mm was placed on the ascending aorta. Animals in the sham operation group went through a similar process without a clip insertion. Then, the supraclavicular incision was closed, and the rat was returned to the cage.

To determine whether ADAMTS8 promoted cardiac fibrosis after myocardial infarction, after the establishment of myocardial infarction model, the visibly ischemic zone of the rat myocardium was injected with adenovirus-mediated overexpression of ADAMTS8 or the corresponding control at 6.0 × 10^10^ vector genome per rat. Two weeks later, another injection was given. The adenovirus with ADAMTS8 overexpression was synthesized by Hanbio Biotechnology, China.

### Isolation and Culture of Primary Cardiac Fibroblasts and Cardiomyocytes

Isolation and culture of cardiac fibroblasts and cardiomyocytes were performed as described previously (19). The ventricles were excised from SD rats of 2-day old, washed, cut into pieces smaller than 1 mm^3^, and incubated overnight at 4°C in D-Hanks' balanced salt solution containing 0.5% trypsin. The small pieces were then collected and further digested by type-II collagenase (100 units/ml; Worthington, USA) for 40 min at 37°C. The digestion was terminated with DMEM (Gibco-Life) supplemented with 10% FBS, and the small pieces were re-suspended by pipetting. The resulting cell suspensions were filtered through a cellular sifter (200-mesh), incubated in DMEM with 10% FBS for 70 min. Cells in suspension were removed and the remaining cells attached to the bottom were collected, re-plated at a density of 3 to 5 × 105 cells/ml, and cultured in DMEM with 10% FBS, 100 units/ml penicillin, 100 μg/ml streptomycin, and 0.1 mM bromodeoxyuridine (BrdU).

### Overexpression of ADAMTS8 in Cardiac Fibroblasts and Cardiomyocytes

Cardiac fibroblasts and cardiomyocytes were transfected with adenovirus according to the manufacturer's adenovirus operating manual. The multiplicity of infection was about 70 pfu number/cell.

### Small Interfering RNA Transfection

According to the product instructions of RiboBio, the small interference targeting ADAMTS8 (sequence: TAGGAGCAAGAG-ATTTGTA) was transfected into cardiomyocytes and fibroblasts. For transfection, cardiac fibroblasts or cardiac myocytes were cultured to 60–80% confluence. 100 nM ADAMTS8-siRNA or scramble-siRNA was delivered into cells by using Lipofectamine RNAiMAX reagent (Life Technologies) according to the manufacturer's protocol (20).

### Co-culture of Cardiac Myocytes and Fibroblasts

One of the methods to co-culture cardiomyocytes was to plant fibroblasts in inserts (8 μm pore size), and cardiomyocytes were cultured in the bottom chamber of a 24-well culture plate and co-cultured together. Another method was placing in insert (0.4 μm pore size) in the wells to place the cardiomyocytes transfected with overexpressed ADAMTS8 adenovirus or siRNA. Cardiac fibroblasts were then cultured in the bottom chamber of a 6-well culture plate and co-cultured together.

### Transwell Migration, Scratch-Wound Assay, and CCK-8 Assay

As described in the previous study, fibroblast migration was observed by the transwell system and scratch-wound (21). Cardiac fibroblasts were prepared at a concentration of 2.0 × 10^5^/ml with serum-free medium. Cardiomyocytes transfected with overexpressed ADAMTS8 adenovirus or siRNA were then cultured in the bottom chamber of a 24-well culture plate. An insert (8 μm pore size) was placed in the wells to place the cardiac fibroblasts (100 μl). After cardiomyocytes and fibroblasts were co-cultured for 24 h, the insert was removed into a new 24-well culture plate containing PBS to remove unattached cells. Cardiac fibroblasts were then fixed by 10% formalin for 15 min, stained with 0.25% crystal violet for 15 min, rinsed again with sterile water, and allowed to dry.

An *in vitro* scratch-wound assay was performed to evaluate cell motility. Thus, cardiac fibroblasts were plated at a density of 5 × 10^5^/well in six-well culture plates. Then, a single scratch was made by a sterile 200 μl micropipette tip when cells reached 90% confluence and an insert (0.4 μm pore size) was placed in the wells to place the cardiomyocytes transfected with overexpressed ADAMTS8 adenovirus or siRNA. After cardiomyocytes and fibroblasts were co-cultured for 24 h, images of the bright field were obtained separately.

For the CCK-8 method, Cardiac fibroblasts were plated in 96-well plates at a density of 5 × 10^3^ cells/well. The cells were exposed to conditioned medium (CM) for up to 48 h. Then, each well was supplemented with 10 μl CCK-8 and incubated at 37°C for 2 h. The optical density was measured at 450 nm and the proliferation was calculated.

### Preparation of Conditioned Medium

Cardiomyocytes were transfected with ADAMTS8 overexpressing adenovirus or ADAMTS8-siRNA. After the incubation period, the medium was collected as conditioned medium (CM).

### Reverse-Transcription Quantitative PCR (qPCR)

Total RNA was extracted from cells using TRIzol (Takara). cDNA was synthesized using with the PrimeScript^TM^ RT Reagent Kit (Takara) and quantitative PCR was performed using SYBR Green (Takara). GAPDH was used as an internal control. The primer sequences are shown in Table 1.

**Table 1:** Primers sequences used for qRT-PCR.

	**Gene**	**Forward primer**	**Reverse primer**
Norway rat	ADAMTS8	CAGCGGCGGACTGTGGAATG	GGCTTGGCATCCTCAGGTTTCAG
Norway rat	Mfn1	CGTGGCAGCAGCAGAGAAGAG	CCTCCTCCGTGACCTCCTTGATC
Norway rat	Mfn2	TCCACAGCCATTGCCAGTTCAC	CCGCACAGACACAGGAAGAAGG
Norway rat	NOX4	ACTGCCTCCATCAAGCCAAGATTC	CCAATGCCTCCAGCCACACAC

### Western Blot Analysis

Protein was extracted from isolated cells and ventricular tissue using RIPA lysis buffer (Beyotine, China) supplemented with the protease and phosphatase inhibitor cocktail (MCE). Proteins were separated on an 8–12% sodium dodecyl sulfate–polyacrylamide gel electrophoresis (SDS-PAGE) and transferred to polyvinylidene difluoride (PVDF) membranes. After being blocked with 5% milk for 1 h, the membranes were incubated with primary antibodies at 4°C overnight. We used the following antibodies: ADAMTS8 (Santa, sc-514717), α-SMA (Abcam, ab32575), Collagen1 (Abclonal, A1352), CTGF (Abcam, ab6992), p-EGFR (Abcam, ab32578), EGFR (Abcam, ab52894), p-ERK (Abclonal, AP0974), ERK (Abclonal, A4782), p-JNK (Abclonal, AP0631), JNK (Abclonal, A4867), p-p38 (Abcam, ab178867), p38 (Abclonal, A4771), p-AKT (Proteintech, 66444-1-Ig), AKT (Proteintech, 10176-2-AP), p-YAP (Cell Signaling, #4911), YAP (Cell Signaling, #14074), TAZ (Cell Signaling, #4883), proliferating cell nuclear antigen (Santa, sc-56), p27kip1 (Abclonal, A19095), dynamin-related protein 1 (DRP-1, Cell Signaling, #8570, 1:500), phosphorylated DRP-1 at Ser616 (Cell Signaling, #4494), phosphorylated DRP-1 at Ser637 (Cell Signaling, #4467), GAPDH (Abcam, ab181602). Then membranes were washed thoroughly and incubated with horseradish peroxidase (HRP)-conjugated secondary antibodies at room temperature for 1 h. Signals were revealed by enhanced chemiluminescence ECL (Thermo Scientific) on an image-capturer (Tanon 5200) and quantified by densitometry software (Image-Pro Plus).

### Histological Analysis and Immunofluorescence Staining

Four weeks after MI or 8 weeks after TAC, rats were euthanized, left ventricle tissues were fixed with phosphate-buffered 10% formalin for 24 h, and then the fixed tissues were paraffin embedded and sliced into 4 μM sections. The extent of interstitial fibrosis was determined by PSR staining according to the instructions. Immunofluorescence staining was performed using antibodies against ADAMTS8 and vimentin according to previously described methods (20). Quantification of the immunopositive area was carried out by means of Image-Pro Plus 6.0.

### Dihydroethidium Staining

The effect of ADAMTS8 on ROS generation was detected by Dihydroethidium (DHE, BestBio, China). After 24 h of virus transfection, cardiac fibroblasts were preloaded with DHE for 20 min. After washing with PBS for three times, DHE fluorescence images were obtained using a fluorescence microscopy (LSM780).

### Mebendazole Treatment *in vitro*

CFs were seeded in 10 cm dish or 6-well plate in DMEM with 10% FBS. CFs were allowed to adhere for 24 h, washed two times, and starved in serum-free medium for 24 h. These quiescent cells were then stimulated with mebendazole (5 mol/L, 0.5% DMSO) or vehicle for 24 h (15).

### Mebendazole Treatment *in vivo*

After 1 week of MI or TAC model, adult male Sprague-Dawley rats (6 weeks of age, 150–170 g) male rats were randomized to be treated with either mebendazole (1% DMSO, 25 mg/kg/day, i.p.) or vehicle for 3 weeks (15). Age-matched control rats received an equal volume of vehicle. Simple randomization method was applied to categorize rats to each group. After the proposed protocol, echocardiography was performed. All analyses were carried out in a blinded manner.

### Echocardiographic Measurements of the Left Ventricle

On the 28th day, echocardiography was performed with ultrasound instrument (Vivid7, GE Healthcare). Rats were anesthetized by intraperitoneal injection of pentobarbital and placed on a heating pad to maintain a body temperature of 37°C (22). M-mode tracing of the LV was obtained from the parasternal long-axis view to measure LV end-diastolic diameter (LVEDD) dimension and LV end-systolic diameter (LVESD) dimension. Left ventricle fractional shortening (FS) was calculated as (LVEDD – LVESD)/LVEDD × 100 and expressed as percentage. Left ventricle ejection fraction was calculated as following: LVEF (%) = [(LVEDD3 – LVESD3)/LVEDD3] × 100.

### Data Analysis

All values are presented as mean ± SEM of independent experiments. Results were analyzed by an unpaired, two-tailed Student *t*-test (two groups) or ANOVA (three or more groups) followed by Bonferroni's correction if needed. All of the statistical tests were performed with the GraphPad Prism software version 5.0, and *P* < 0.05 was considered to be statistically significant.

## Results

### ADAMTS8 Expression Was Increased in DCM Patients With Severe Cardiac Fibrosis

**Figure 1:**
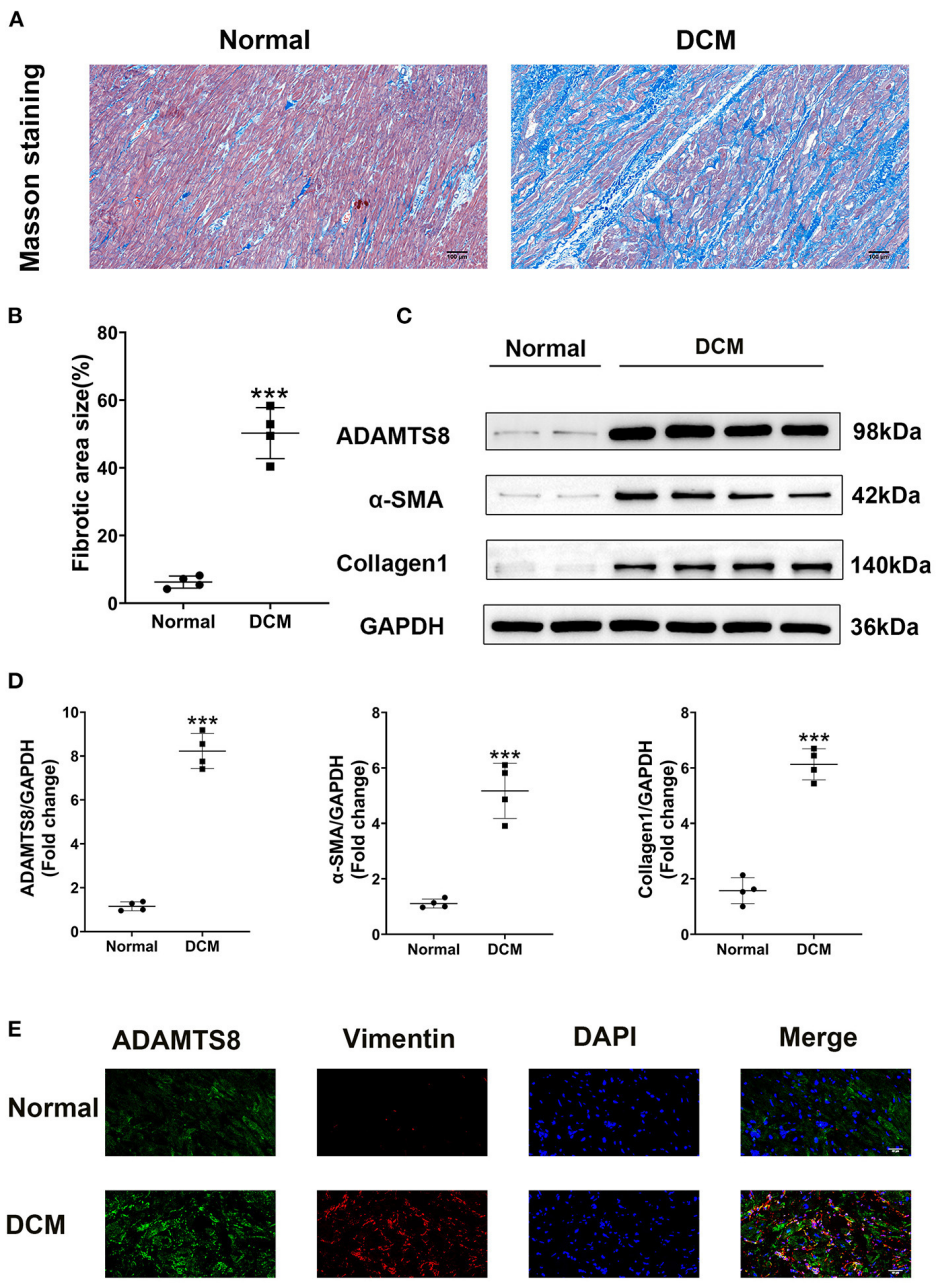
ADAMTS8 expression was increased in DCM patients with severe cardiac fibrosis. **(A,B)** Representative Masson staining (scale bar = 100 μm) and quantification of fibrosis. **(C,D)** Representative western blotting and quantification of ADAMTS8, α-SMA, and Collagen1 expression in the ventricular septum from hearts obtained from patients with DCM and normal hearts. **(E)** Immunofluorescence staining of ADAMTS8 and Vimentin in the ventricular septum from hearts from patients with DCM and normal hearts (scale bar = 40 μm). Data were presented as mean ± SEM. ***P < 0.001 vs. Normal group.

We compared ADAMTS8 expression in normal hearts obtained from healthy donors in traffic accidents and failing hearts obtained from patients with dilated cardiomyopathy (DCM) undergoing surgery. The characteristics of healthy controls and patients with DCM were listed in the Supplementary Table 1. Masson staining revealed severe cardiac fibrosis in the failing hearts compared with normal hearts (Figures 1A,B). Western blotting confirmed that the expression of ADAMTS8 was increased in patients with heart failure, and the production of α-SMA and type I collagen was also increased (Figures 1C,D). Immunofluorescence showed that ADAMTS8 and vimentin were significantly increased in patients with heart failure (Figure 1E).

### Increased ADAMTS8 Expression Was Associated With Cardiac Fibrosis in the MI Rat Model

**Figure 2:**
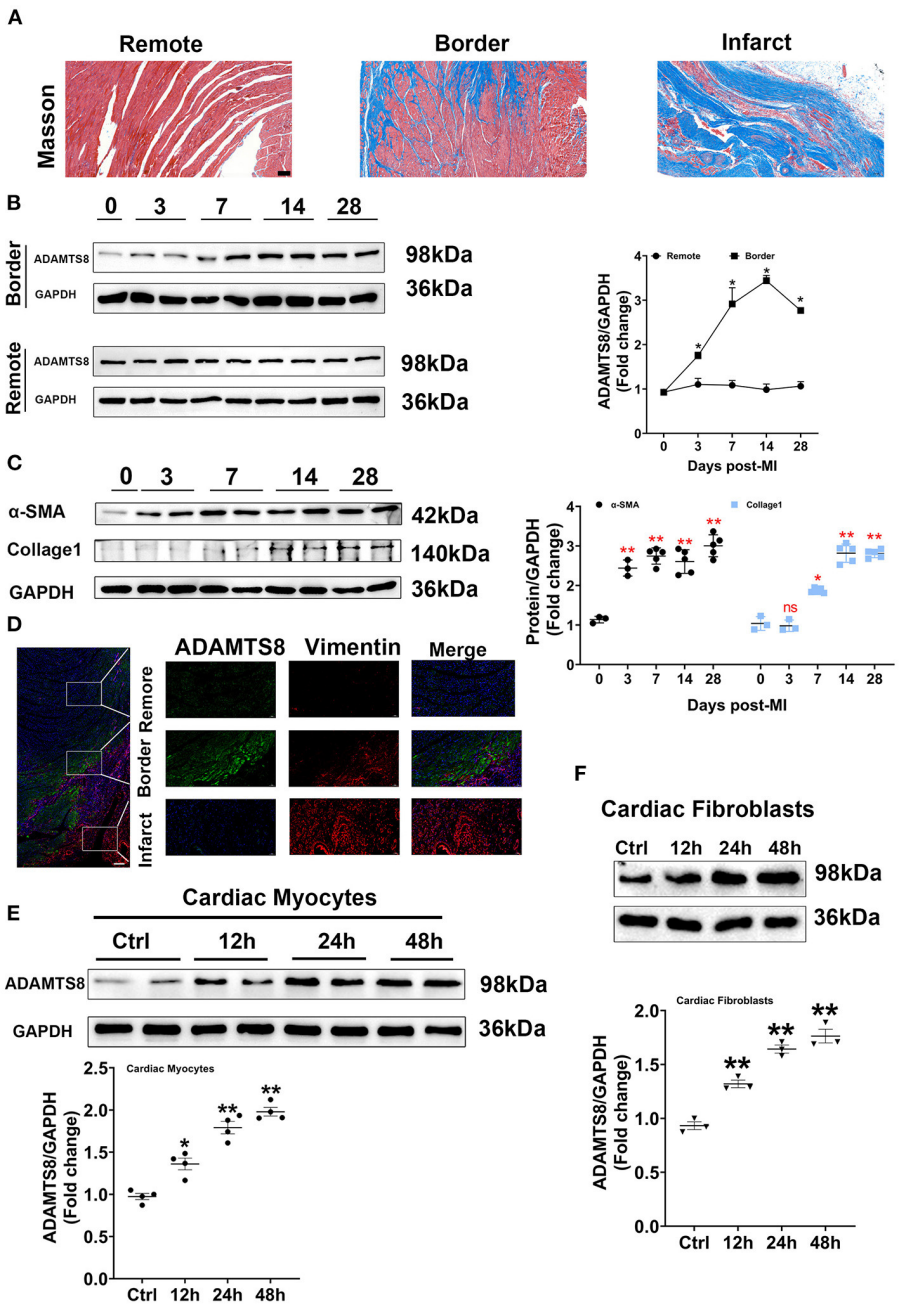
Increased ADAMTS8 expression was associated with cardiac fibrosis in the MI rat model. **(A)** Representative Masson staining images from the heart of the MI rat model (scale bar = 100 μm). **(B)** Changes of ADAMTS8 protein expressions in the border zone and the remote area at the indicated time points post-MI. **(C)** Western blotting and quantification of the expressions of α-SMA and Collagen I in infarcted and border zones of rat hearts. **(D)** Representative images of double immunofluorescence staining of ADAMTS8 (green) and vimentin (red) (scale bar = 200 μm). **(E,F)** The ADAMTS8 protein level in cardiac myocytes and cardiac fibroblasts under hypoxia conditions for 12, 24, and 48 h assessed *via* western blotting. Data were presented as mean ± SEM. *P < 0.05 vs. 0 day (Sham). **P < 0.01 vs. 0 day (Sham) or ctrl group.

To explore whether the expression of ADAMTS8 was related to cardiac fibrosis after myocardial infarction injury and pressure overload, the MI and TAC-induced heart failure rat models were used to represent the above two different cardiac fibrosis responses. First, the successful modeling was confirmed by Masson staining (Figure 2A). In the MI rat models, we detected the spatial expression of ADAMTS8 in the border zone of the infarct and the remote area from the infarct by western blot. The level of ADAMTS8 increased in the border zone on the 3rd day after MI, significantly increased on the 7th day, peaked on the 14th day, and then decreased slightly on the 28th day. However, ADAMTS8 levels in the remote area from the infarct did not change significantly throughout 28 days post-MI (Figure 2B). The expression of α-SMA and collage1 in the border zone gradually increased after myocardial infarction (Figure 2C). The weak staining of ADAMTS8 in the remote area was examined by immunofluorescence analysis and vimentin-positive fibroblasts were not detected in the remote area (Figure 2D). However, the ADAMTS8 staining was strongly positive in the border zone in rats 28 days post-MI (Figure 2D). Furthermore, we investigated the expression of ADAMTS8 in cardiac fibroblasts and cardiomyocytes at different time points after hypoxia exposure. ADAMTS8 expression was increased after 24 h of hypoxia, remained elevated after 48 h, and was increased more significantly in cardiomyocytes (Figures 2E,F). Collectively, the above results suggested that ADAMTS8 cuold be involved in myocardial infarction.

### ADAMTS8 Expression Was Increased in the TAC-Induced Cardiac Fibrosis Rat Models

**Figure 3:**
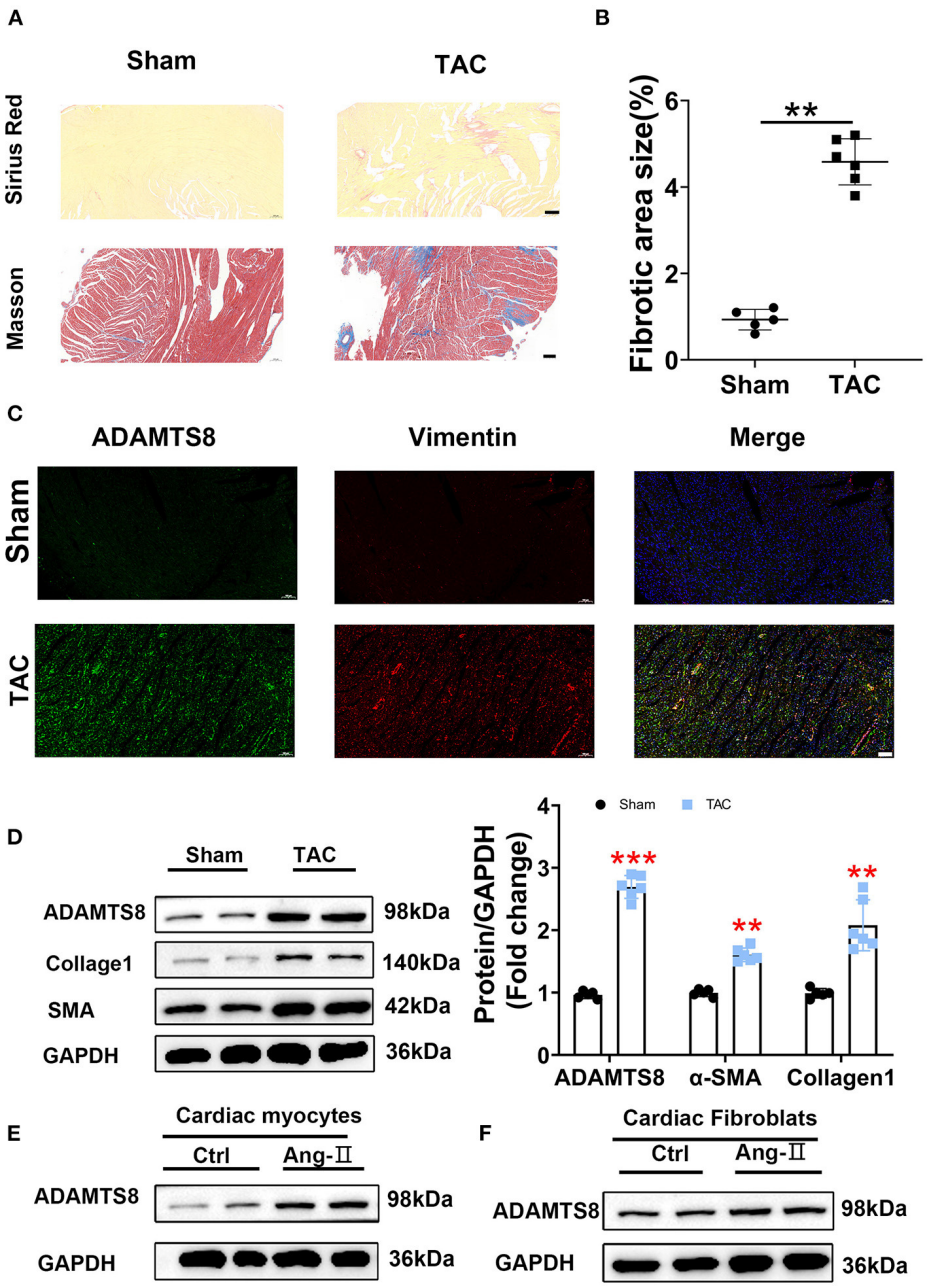
ADAMTS8 expression was increased in the TAC-induced cardiac fibrosis rat model. **(A,B)** Representative Sirius Red and Masson staining images from a sham heart and a TAC-induced heart (scale bar = 200 μm). **(C)** Immunofluorescence staining of ADAMTS8 (green) and vimentin (red) in normal and TAC-induced rats (scale bar = 100 μm). **(D)** ADAMTS8, α-SMA, and Collagen1 expressions in the sham group and TAC model group *via* western blotting and quantified according to the immunoblots. **(E,F)** The ADAMTS8 protein level in cardiac myocytes and cardiac fibroblasts treated with PBS or Ang-II. Data were presented as mean ± SEM. **P < 0.01, ***P < 0.001 vs. sham.

Similar results to the MI rat model were obtained in the TAC-induced cardiac fibrosis. Successful modeling was confirmed by Sirius red and Masson staining (Figures 3A,B). The increased expression of ADAMTS8 was examined by immunofluorescence staining in hypertrophic myocardial tissues (Figure 3C). Similarly, the western blot results showed increased expressions of ADAMTS8, α-SMA, and collagen1 in fibrotic hearts (Figure 3D). At the same time, we detected the expression of ADAMTS8 in cardiomyocytes and cardiac fibroblasts stimulated with Ang-II (1 μM); the protein expression of ADAMTS8 was upregulated in these cells after Ang-II stimulation (Figures 3E,F). Collectively, these results demonstrated that increased ADAMTS8 expression was related to cardiac fibrosis in rats with cardiac injury or pressure overload.

### ADAMTS8 Promoted Myofibroblast Formation *in vitro*

**Figure 4:**
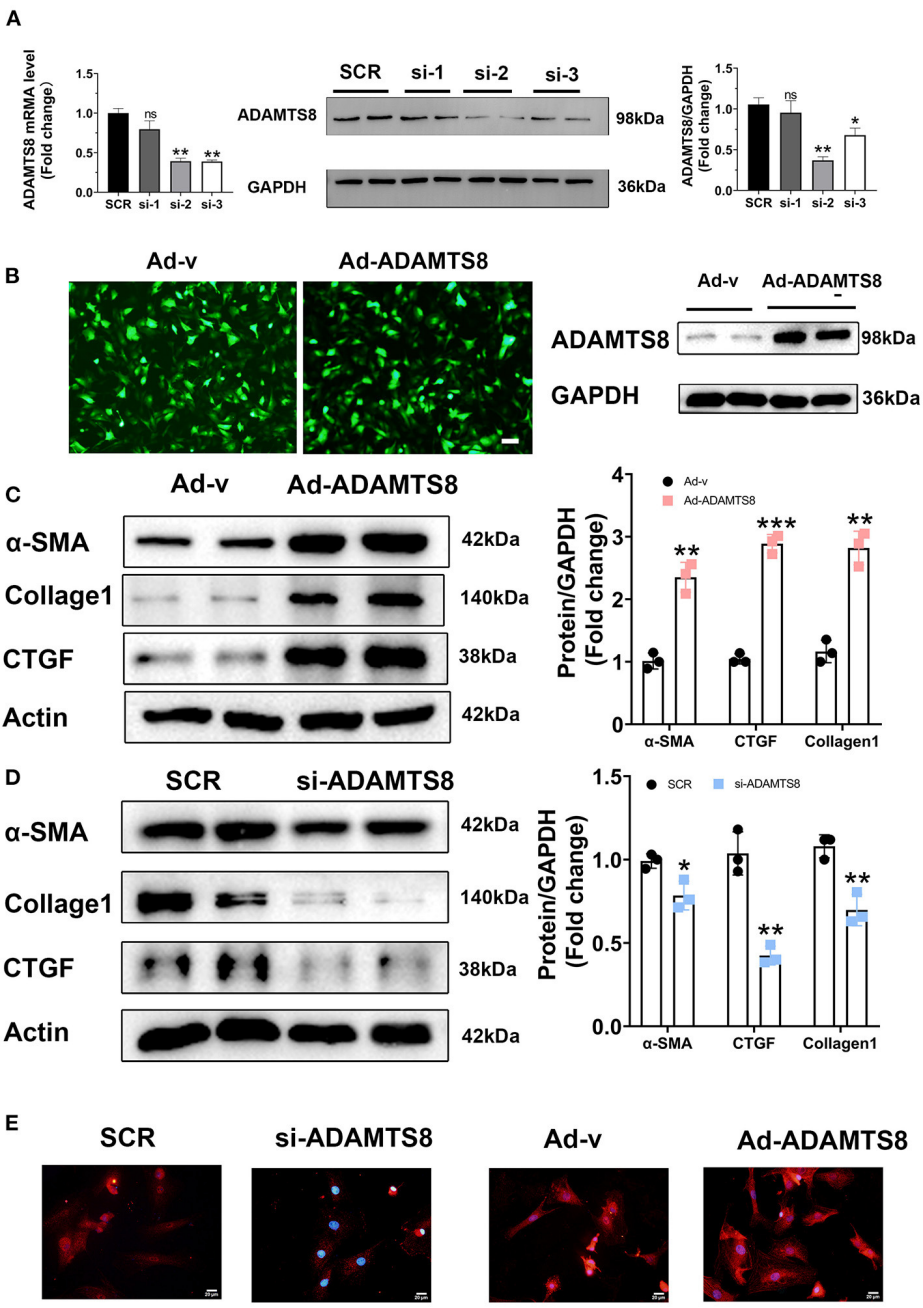
ADAMTS8 promoted myofibroblast formation *in vitro*. **(A)** Knockdown efficiency of three different interference sequences of ADAMTS8 detected by qPCR and western blotting. **(B)** The overexpression efficiency of ADAMTS8 verified by fluorescence and Western blotting. **(C,D)** α-SMA, Collagen I, and CTGF levels in cultured cardiac fibroblasts infected with ADAMTS8-overexpressing adenovirus or si-ADAMTS8 detected *via* western blotting. **(E)** Immunofluorescence for the expression of α-SMA (red) and nuclei (DAPI: blue) (scale bar = 20 μm). Data were presented as mean ± SEM. *P < 0.05, **P < 0.01, ***P < 0.001, and NS indicates no significance vs. Ctrl or between the two indicated groups.

In a previous study on the chronic hypoxia-induced pulmonary hypertension model, the ADAMTS8-specific knockout mice of cardiomyocytes had significantly less right ventricular fibrosis than control mice (15). Our findings showed that the expression changes of ADAMTS8 in cardiac myocytes were more obvious than in cardiac fibroblasts under hypoxia and angiotensin II stimulation. Considering that cardiac fibrosis is mainly mediated by the activation of resident cardiac fibroblasts and the main function of ADAMTS8 is to degrade the key components of the extracellular matrix (23), we speculated that ADAMTS8 could be secreted by cardiomyocytes and then activated cardiac fibroblasts by paracrine, thereby affecting cardiac fibrosis. To confirm this hypothesis, we first explored whether ADAMTS8 affects fibroblasts. Three ADAMTS8-specific siRNAs were used to transfect cardiac fibroblasts. After 48 h of transfection, it was confirmed that the si2 knockdown efficiency was the highest by qPCR and western blot (Figure 4A). We also constructed an adenovirus with overexpressed ADAMTS8 (Ad-ADAMTS8) and a positive control adenovirus (Ad-v). Virus transfection efficiency was verified by immunofluorescence and WB (Figure 4B). Cultured cardiac fibroblasts from neonatal rats were transfected with Ad-ADAMTS8 or si-ADAMTS8 to further explore the role of ADAMTS8 in cardiac fibrosis and the underlying mechanisms. ADAMTS8 overexpression promoted the differentiation of fibroblasts into myofibroblasts, which was manifested by the increased expression of α-SMA, collagen1 and CTGF (Figure 4C). Conversely, ADAMTS8 knockdown inhibited myofibroblast activation (Figure 4D) and the expression of α-SMA in the ADAMTS8-siRNA group was significantly reduced compared with the scramble-siRNA group by immunofluorescence staining. At the same time, treatment with Ad-ADAMTS8 induced a remarkable elevation in α-SMA expression compared with the Ad-v group, suggesting that ADAMTS8 could promote fibroblast to differentiate into myofibroblasts (Figure 4E). These results provided evidence that ADAMTS8 exacerbated cardiac fibrosis *in vitro*.

### ADAMTS8 Mediated the Interaction Between Cardiomyocytes and Cardiac Fibroblasts

**Figure 5:**
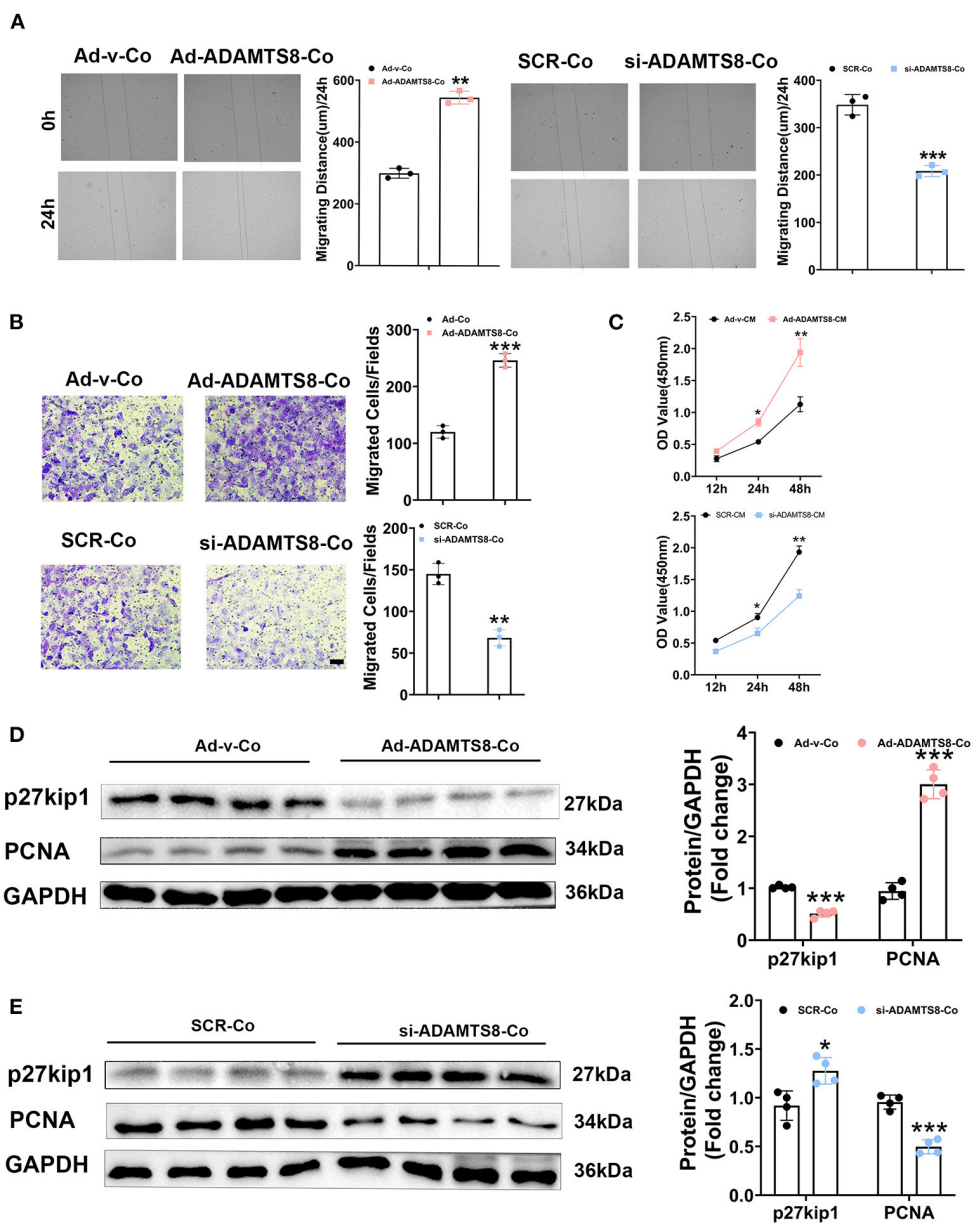
ADAMTS8 mediated the interaction between cardiomyocytes and cardiac fibroblasts. **(A)** Wound repair assays to assess the migration ability of cardiac fibroblasts co-cultured with cardiomyocytes transfected with ADAMTS8 overexpressing adenovirus (Ad-ADAMTS8-Co) or siRNA (si-ADAMTS8-Co). **(B)** Transwell migration assay and quantification of migrated cardiac fibroblasts (scale bar = 50 μm). **(C)** Quantification by CCK-8 assay (*n* = 4 in each group). **(D,E)** p27kip1 and PCNA levels in cardiac fibroblasts co-cultured with cardiomyocytes transfected with ADAMTS8 overexpressing adenovirus (Ad-ADAMTS8-Co) or siRNA (si-ADAMTS8-Co) detected *via* western blotting. Data were presented as mean ± SEM. *P < 0.05, **P < 0.01, and ***P < 0.001 between the two indicated groups.

Cardiomyocytes and fibroblasts are key cell types in the heart that collaborate to regulate normal cardiac function and the heart's response to pathogenic stimuli (24). ADAMTS8, as a paracrine mediator, has been reported to play a crucial role in the interaction between pulmonary artery smooth muscle cells (PASMCs) and pulmonary artery endothelial cells (PAECs) in the development of pulmonary hypertension (PH) (15). The expression of ADAMTS8 in cardiomyocytes increased more obviously after hypoxia or by Ang-II or stimulation. The previous study has shown that the ADAMTS8-specific knockout of mice cardiomyocytes had significantly less right ventricular fibrosis than the control mice. Thus, we next assessed the effects of ADAMTS8 cardiomyocyte-derived as a paracrine signaling on cardiac fibroblasts. We co-cultured cardiac fibroblasts with cardiomyocytes transfected with overexpressed ADAMTS8 adenovirus (Ad-ADAMTS8-Co) or siRNA (si-ADAMTS8-Co). As expected, the co-culture of cardiomyocytes transfected with overexpressed ADAMTS8 adenovirus enhanced the migration capability of the cardiac fibroblasts isolated from SD rats, but si-ADAMTS8-Co inhibited fibroblasts migration (Figures 5A,B). To confirm our findings, we prepared a conditioned medium (CM) from cardiomyocytes transfected with Ad-ADAMTS8 (Ad-ADAMTS8-CM) or si-ADAMTS8 (si-ADAMTS8-CM) for a CCK8 assay. The results showed that cell proliferation was also decreased in cardiac fibroblasts cultured with si-ADAMTS8-CM compared with those cultured with control CM (Figure 5C). Moreover, cardiac fibroblasts co-cultured with cardiomyocytes transfected with overexpressed ADAMTS8 adenovirus increased the expression of proliferating cell nuclear antigen (PCNA) but decreased the expression of kip27 (an inhibitor of a cyclin-dependent kinase). In contrast, the co-culture with si-ADAMTS8-Co decreased the expression of PCNA and increased the expression of p27Kip1 of CFs (Figures 5D,E). Hence, these data suggested that the secretion of ADAMTS8 from cardiomyocytes might be associated with the interaction between cardiomyocytes and cardiac fibroblasts in the development of cardiac fibrosis.

### ADAMTS8-Mediated Mitochondrial Dysfunction in Cardiac Fibroblasts and Activated Ros-Sensitive Pathways to Regulate the Fibrotic Response

**Figure 6:**
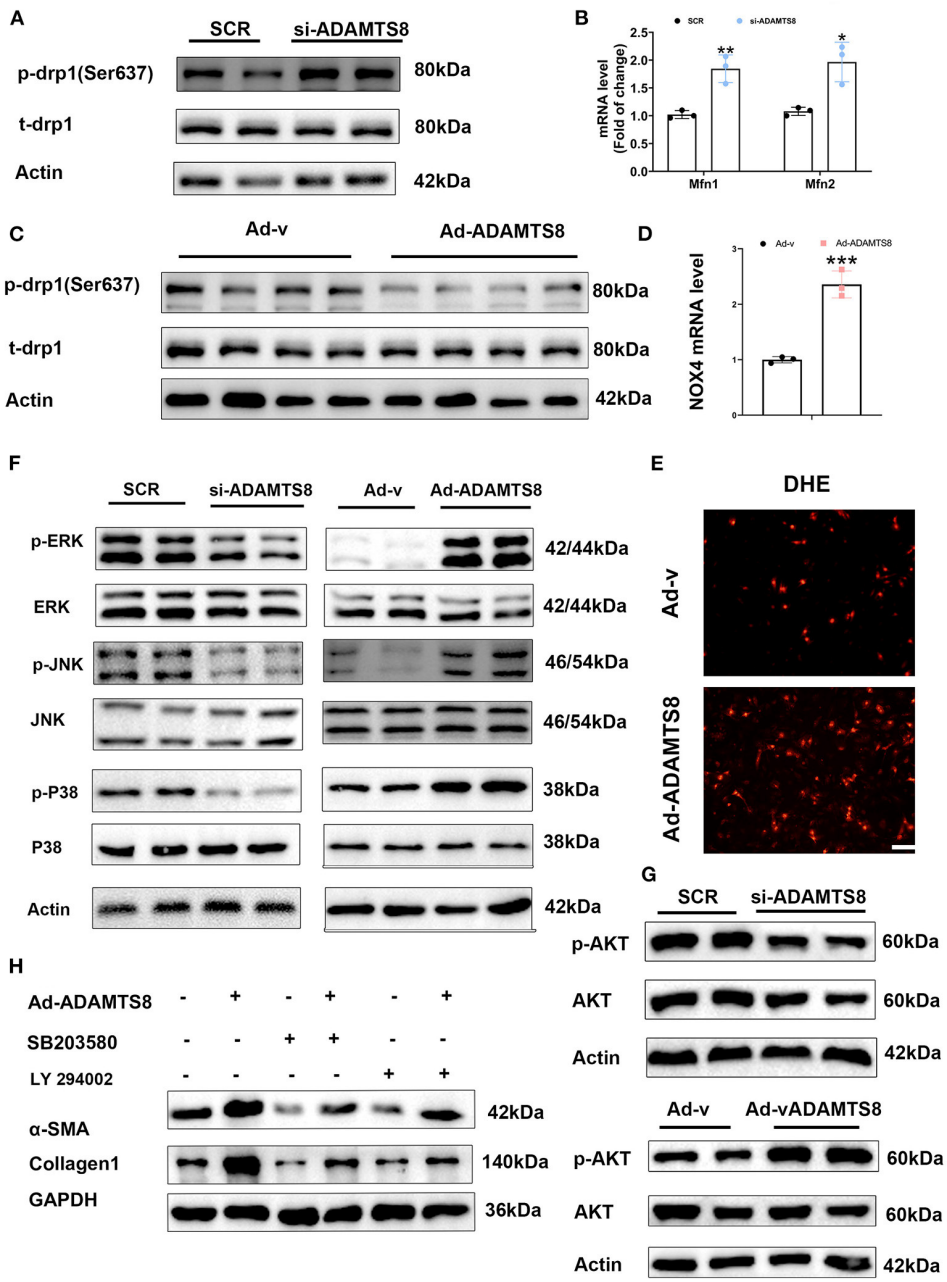
ADAMTS8-mediated mitochondrial dysfunction in cardiac fibroblasts and activated ROS-sensitive pathways to regulate the fibrotic response. **(A,C)** Representative Western blot of DRP-1 (p-drp1) phosphorylated at Ser637 and total drp1 (t-drp1) in cultured cardiac fibroblasts infected with si-ADAMTS8 or ADAMTS8-overexpressing adenovirus. **(B)** Real-time polymerase chain reaction (RT-PCR) analyses of Mfn1 and Mfn2 mRNA in cultured cardiac fibroblasts infected with si-ADAMTS8. Average expression values were normalized to GAPDH mRNA. **(D)** RT-PCR analyses of NOX4 mRNA in cultured cardiac fibroblasts infected with ADAMTS8-overexpressing adenovirus average expression values were normalized to GAPDH mRNA. **(E)** Representative images of dihydroethidium (DHE) staining of CFs infected with ADAMTS8-overexpressing adenovirus. **(F,G)** Activation of MAPK and AKT in cultured cardiac fibroblasts overexpressing ADAMTS8 or downregulating ADAMTS8 detected *via* western blotting. **(H)** Cultured cardiac fibroblasts infected with ADAMTS8 overexpressing adenovirus treated with the MAPK pathway inhibitor SB203580 or AKT inhibitor LY294002 (25 uM) after quiescence, followed by analysis of the cell lysates with indicated antibodies. Data were presented as mean ± SEM. *P < 0.05, **P < 0.01, and ***P < 0.001 between the two indicated groups.

ADAMTS8 promotes mitochondrion division in pulmonary artery smooth muscle cells, resulting in increased ROS production and cell proliferation (15). Recent studies have shown an emerging role of reactive oxygen species (ROS) and mitochondrial function in CF proliferation and activation. Meanwhile, ROS production induced by mitochondrial fission activates the phosphorylation of p38-MAPK to increase proliferation and collagen production in rat cardiac fibroblasts (25, 26). In lung fibroblasts, transcriptionally TGFβ-induced mitochondrial ROS generation resulted in increased NOX4 expression, which was considered to be a way to sustain the elevated intracellular ROS levels to activate the PI3K-AKT and MAPK signaling pathways (27). Therefore, we explored whether the effect of ADAMTS8 on cardiac fibroblasts was related to the balance between mitochondrial fission and fusion in cells, thereby activating the PI3K-AKT and MAPK signaling pathways. It is known that drp1 is phosphorylated at Ser637, which inhibits the division of mitochondria (28). We found that the ADAMTS8-siRNA treatment activated drp1 phosphorylation (Ser637) (Figure 6A). RT-PCR showed that pro-fusion genes (Mfn1 and Mfn2) were significantly up-regulated in the si-ADAMTS8 group compared with the SCR group (Figure 6B). Consistently with these findings, the phosphorylation level of drp1 at Ser637 was significantly decreased by the overexpression of ADAMTS8 (Figure 6C). Furthermore, ADAMTS8 overexpression increased NOX4 expression and ROS level (Figures 6D,E). To elucidate the potential molecular mechanism of ADAMTS8 on fibroblasts, we analyzed whether ADAMTS8 affected the PI3K-AKT and MAPK signaling pathways which were mentioned above. Consistent with previous studies (29, 30), the phosphorylation levels of ERK1/2, JNK, and p38 were decreased in the si-ADAMTS8 group, whereas all of them were significantly increased in the ADAMTS8 overexpression group (Figure 6F). At the same time, the AKT signaling pathway was inhibited in the si-ADAMTS8 group, while it was significantly activated in the ADAMTS8 overexpression group (Figure 6G). Finally, the effect of ADAMTS8 on fibroblast activation was mostly abolished by the MAPK pathway inhibitor SB203580 and the AKT inhibitor LY294002 (Figure 6H). Collectively, these results indicated that ADAMTS8 is one possible mechanism of action for mitochondrial dysfunction, which results in ROS-dependent activation of the p38-MAPK and AKT pathways to increase proliferation and collagen production in CFs.

### ADAMTS8 Activated YAP, and Inhibiting the EGFR Expression Blocked the ADAMTS8-Induced YAP Activation in Cultured Cardiac Fibroblasts

**Figure 7:**
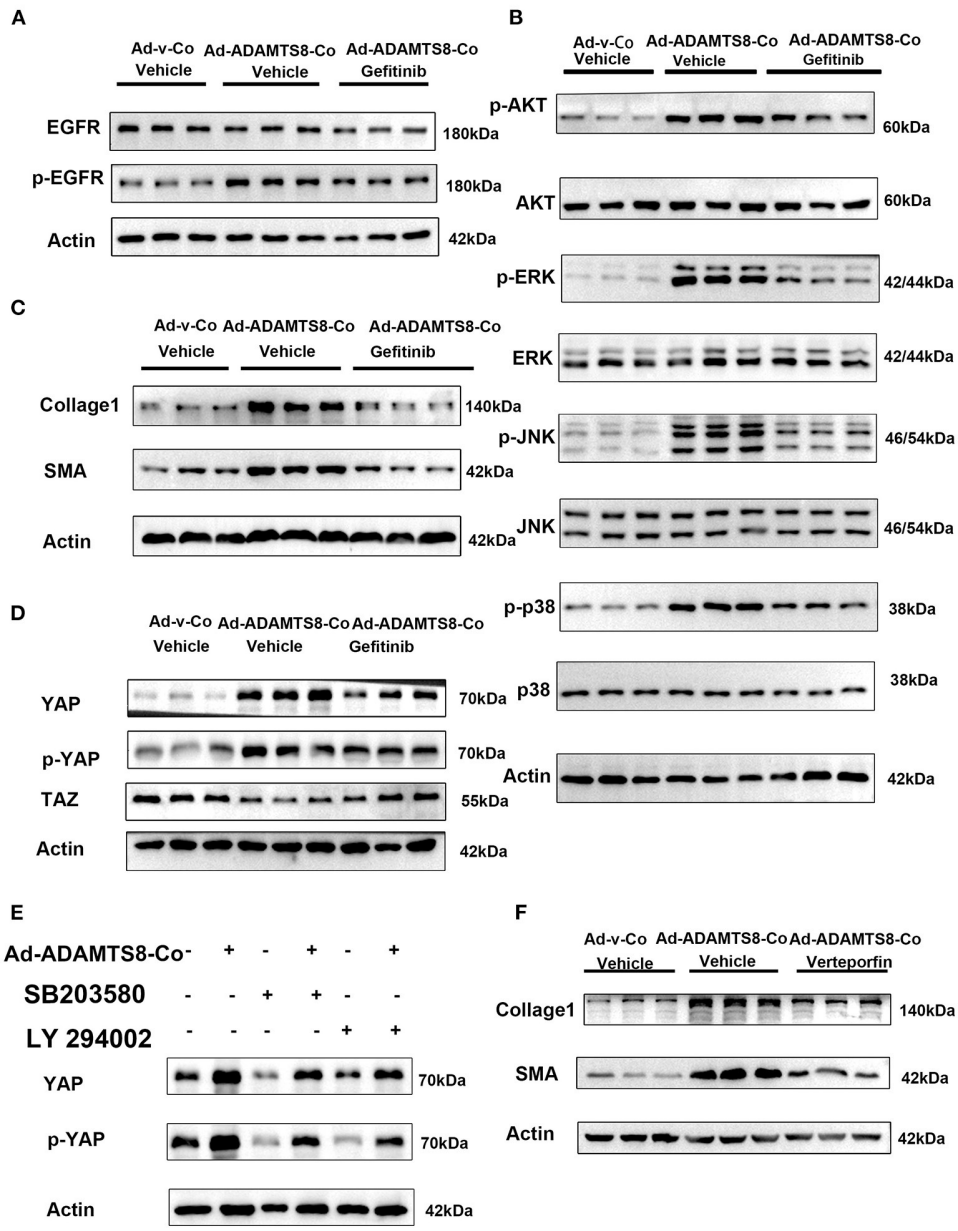
ADAMTS8 activated YAP, and inhibiting the EGFR expression blocked the ADAMTS8-induced YAP activation in cultured cardiac fibroblasts. **(A,B)** The phosphorylation levels of EGFR and its downstream MAPK and AKT were all attenuated in the gefitinib group. **(C)** EGFR inhibitor abated ADAMTS8-induced cardiac fibroblast activation. **(D)** Cardiac fibroblasts co-cultured with cardiomyocytes transfected with ADAMTS8 overexpressing adenovirus (Ad-ADAMTS8-Co) were treated with or without gefitinib, followed by analysis of the cell lysates with indicated antibodies. **(E)** The protein levels of collagen1 and α-SMA were all partly declined in the verteporfin group. **(F)** Cardiac fibroblasts co-cultured with cardiomyocytes infected with ADAMTS8 overexpressing adenovirus were treated with the MAPK pathway inhibitor SB203580 or AKT inhibitor LY294002 after quiescence, followed by analysis of the cell lysates with indicated antibodies.

It is known that proteoglycans, which are crucial components of ECM can be degraded by ADAMTS8 (23). ECM affects cellular behavior in physiological and pathological processes and provides structural support. ECM can also release growth factors locally, such as epidermal growth factor (EGF), which can act as soluble ligands binding to EGFR. Thus, ECM remodeling through proteolytic degradation can release these growth factors, affecting cell proliferation and migration (31). Furthermore, the ERK and PI3K-AKT pathways activated by ADAMTS8 are distinct effector pathways activated by EGFR (32). Thus, we hypothesized that the fragments of ECM proteins released by ADAMTS8 could act as soluble ligands to interact with EGFR and then activate the ERK and PI3K signaling pathways. Therefore, we co-cultured cardiac fibroblasts with cardiomyocytes transfected with overexpressed ADAMTS8 adenovirus (Ad-ADAMTS8-Co). The result showed that the phosphorylation level of EGFR in fibroblasts was increased. At the same time, pretreatment with EGFR tyrosine kinase inhibitor gefitinib partly downregulated the ADAMTS8-induced p-Akt/Akt, p-ERK/ERK, p-JNK/JNK, and p-p38/p38 ratios (Figures 7A,B). Increased α-SMA and collage1 expressions induced by ADAMTS8 were also significantly attenuated by gefitinib pretreatment (Figure 7C). These findings indicated that the differentiation of fibroblasts into myofibroblasts by ADAMTS8 was at least partially mediated by activating the EGFR signaling pathway. Several studies in Drosophila and diabetic renal interstitial fibrogenesis have suggested that activating the PI-3 kinase-Akt and MAPK pathways leads to YAP activation (33, 34), and EGFR is the upstream of the two pathways. At the same time, a study showed that cardiac fibrosis was attenuated by blockade of fibroblast YAP post-MI (35). Thus, we hypothesized that the activation of ERK and PI3K-Akt signals depended on EGFR, and their interaction was upstream of YAP activation after ADAMTS8 overexpression. As we expected, YAP expression and YAP phosphorylation at serine 127 (s127) were upregulated and TAZ (another hippo signaling effector) was downregulated in CFs co-cultured with cardiomyocytes transfected with overexpressed ADAMTS8 adenovirus (Ad-ADAMTS8-Co). Treatment with gefitinib (an EGFR tyrosine kinase inhibitor) reversed the increased YAP expression and YAP phosphorylation and decreased TAZ expression in CFs (Figure 7D). Furthermore, we found that the upregulation of collagen I and a-SMA in CFs co-cultured with cardiomyocytes transfected with overexpressed ADAMTS8 adenovirus (Ad-ADAMTS8-Co) was significantly inhibited by treatment with a YAP inhibitor, verteporfin, suggesting that YAP was downstream of the pathway mentioned above (Figure 7E). At the same time, inhibiting MAPK with SB203580 or inhibiting PI3K with LY294002 also inhibited the increased YAP expression and YAP phosphorylation induced in cardiac fibroblasts co-cultured with cardiomyocytes transfected with overexpressed ADAMTS8 adenovirus (Ad-ADAMTS8-Co) (Figure 7F). These results indicated that ADAMTS8 mediated YAP expression, and EGFR was upstream.

### ADAMTS8 Overexpression Promoted Cardiac Fibrosis and Impaired Cardiac Function *in vivo*

**Figure 8:**
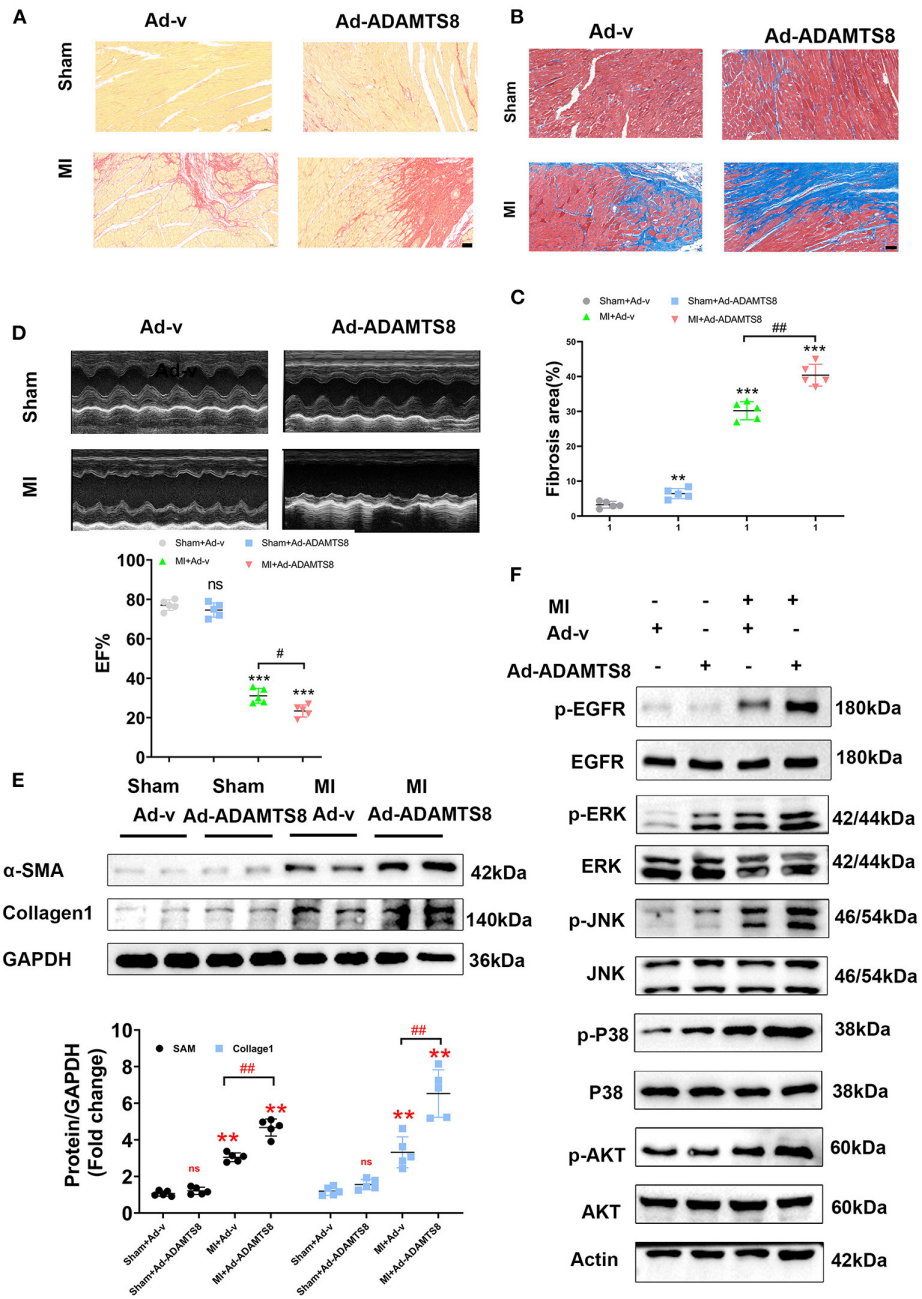
ADAMTS8 overexpression promoted cardiac fibrosis and impaired cardiac function *in vivo*. **(A–C)** Representative pictures from Sirius red and Masson staining and quantitative analysis of interstitial fibrotic area in the infarct border zone of Ad-v Sham, Ad-ADAMTS8 Sham, Ad-v MI, and Ad-ADAMTS8 MI rats (scale bar = 50 μm). **(D)** Representative M-mode images of Ad-v Sham, Ad-ADAMTS8 Sham, Ad-v MI, and Ad-ADAMTS8 MI groups; EF was quantified *via* echocardiography. **(E)** Collage1 and α-SMA expression levels were quantified by western blotting. **(F)** Activation of the EGFR, MAPK, and AKT signaling pathways of a rat model of MI following adenovirus injection was detected *via* western blotting *in vivo*. Data were presented as mean ± SEM. **P < 0.01, ***P < 0.001, ^#^*P* < 0.05, ^*##*^*P* < 0.01, and NS indicates no significance between the two indicated groups.

To explore the role of ADAMTS8 in cardiac fibrosis following injury, after rats were subjected to LAD artery ligation, we injected adenovirus carrying overexpressed ADAMTS8 and positive control adenovirus into the myocardium at multiple locations around the infarct border zone (Supplementary Figure 1A). The expression level of ADAMTS8 was detected by western blot 7 days after MI. The results showed that ADAMTS8 expression was significantly higher in the MI-Ad-ADAMTS8 group than that in the MI-Ad-v group (Supplementary Figure 1B). The results of Sirius red staining and Masson staining showed that overexpression of ADAMTS8 aggravated the degree of cardiac fibrosis (Figures 8A–C). Echocardiography was performed to assess whether the cardiac function was affected by ADAMTS8 overexpression. Compared with the rats injected with positive control virus, the cardiac function of the rats injected with overexpressed ADAMTS8 adenovirus was significantly decreased after myocardial infarction (Figure 8D). Consistent with the above results, compared with the rats injected with control virus, the expression levels of Collage1 and α-SMA were higher in rats injected with overexpressed ADAMTS8 adenovirus than rats injected with control virus, indicating that ADAMTS8 played a key role in activating cardiac fibroblasts *in vivo* (Figure 8E). Additionally, we examined the EGFR, MAPK, and AKT pathways in the rat MI models and found that the phosphorylation levels of EGFR, ERK, JNK, p-38, and AKT were significantly increased in the ADAMTS8 overexpression MI group compared with the scramble MI group (Figure 8F). In summary, the results above suggested that overexpression of ADAMTS8 promoted cardiac fibrosis and impaired cardiac function *in vivo*.

### Mebendazole Down-Regulates ADAMTS8 and Ameliorates Cardiac Fibrosis and Heart Failure

**Figure 9:**
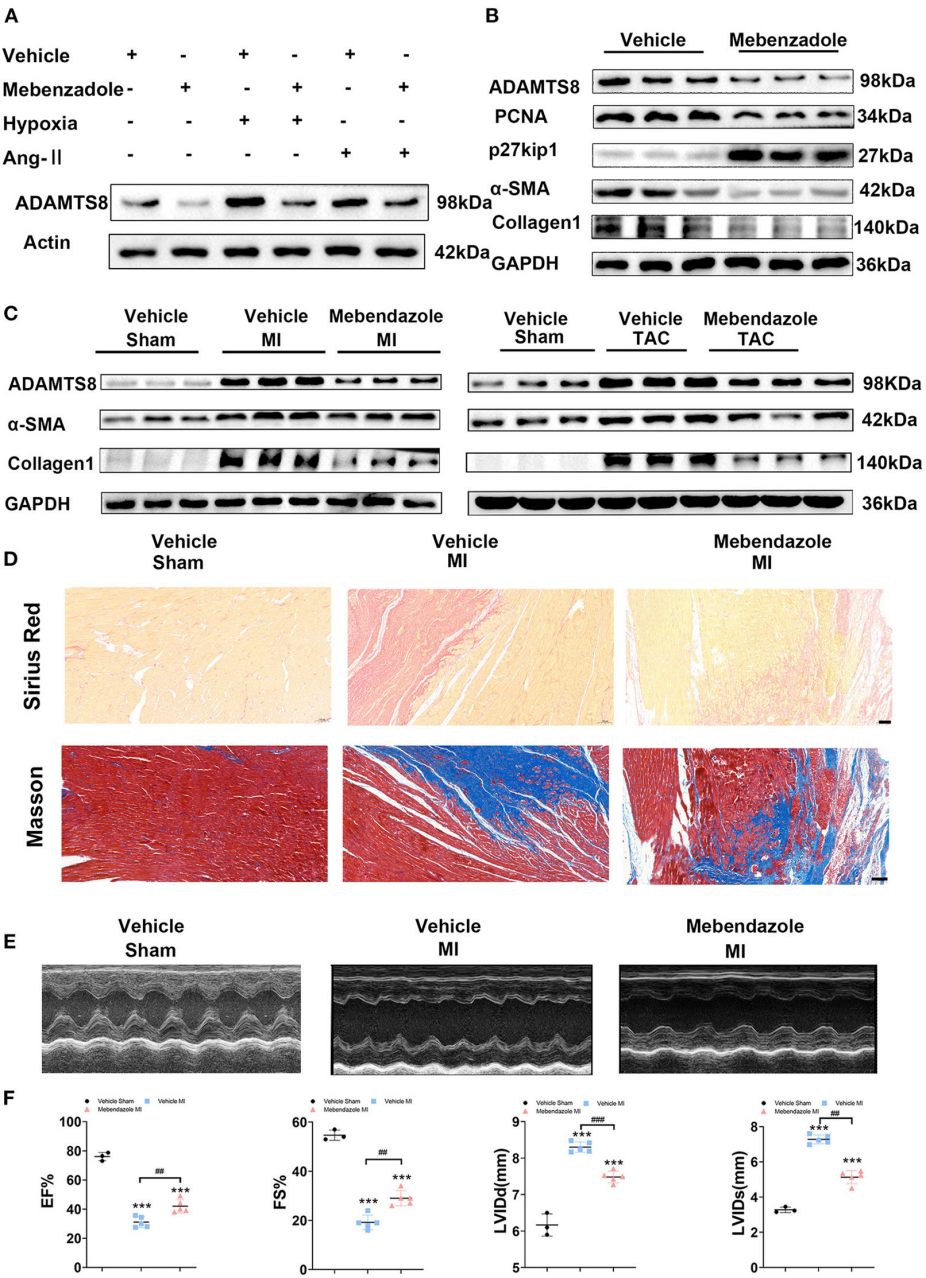
Mebendazole down-regulated ADAMTS8 and ameliorated cardiac fibrosis and heart failure. **(A)** Representative Western blots and quantification of ADAMTS8 in cardiac fibroblasts treated with mebendazole (5 mol/L) for 48 h (*n* = 3 each). **(B)** Representative Western blots and quantification of ADAMTS8, PCNA, p27kip1, α-SMA, and Collage1 expression in CFs treated with or without Mebendazole (*n* = 3 each). **(C)** Representative Western blotting of ADAMTS8, α-SMA, and Collagen1 protein levels in the LVs of rats followed by treatment with vehicle or mebendazole for 14 days after MI or TAC (*n* = 5 each). **(D)** Representative pictures from Sirius red and Masson staining in the infarct border zone of rats followed by treatment with vehicle or mebendazole for 21 days after MI(scale bar = 100 μm). **(E,F)** Representative M-mode echocardiograms obtained on 28th day post-MI. Cardiac function was measured by detecting EF%, FS%, LVDD, and LVSD post-MI. Data were presented as mean ± SEM. ***P < 0.001, ^##^*P* < 0.01 and ^###^*P* < 0.001 between the two indicated groups.

It has been reported that mebendazole could suppress ADAMTS8 expression in the lung and right ventricle and ameliorates pulmonary hypertension (15). Therefore, we explored whether mebendazole could also inhibit cardiac fibrosis by inhibiting ADAMTS8 expression. We found that mebendazole treatment suppressed *in vitro* ADAMTS8 expression in cardiac fibroblasts under normoxic and hypoxic conditions. Mebendazole also suppressed ADAMTS8 expression induced by Ang-II (Figure 9A). Mebendazole treatment reduced the proliferation of fibroblasts while inhibiting the transformation of fibroblasts into myofibroblasts (Figure 9B). We then examined the effect of mebendazole administration in the MI and TAC rat models. The results showed that ADAMTS8 expression in the heart was significantly attenuated by mebendazole treatment in the MI and TAC rat models. In addition, the expression levels of α-SMA and Collage1 were greatly decreased concomitantly in the TAC or MI surgery group receiving mebendazole (Figure 9C). Mebendazole also significantly reduced interstitial fibrosis in the infarct border zone at 28 days after myocardial infarction (Figure 9D). Although LVEF and FS were both decreased at 28 days post-MI, the mebendazole treatment group showed a less-dilated LV dimensions (LVEDD, LVESD) and significantly less-deteriorated LV systolic function compared with the vehicle treatment group (Figures 9E,F). These results demonstrate that mebendazole suppressed ADAMTS8 expression in the heart and ameliorated cardiac fibrosis and heart failure.

**Figure 10:**
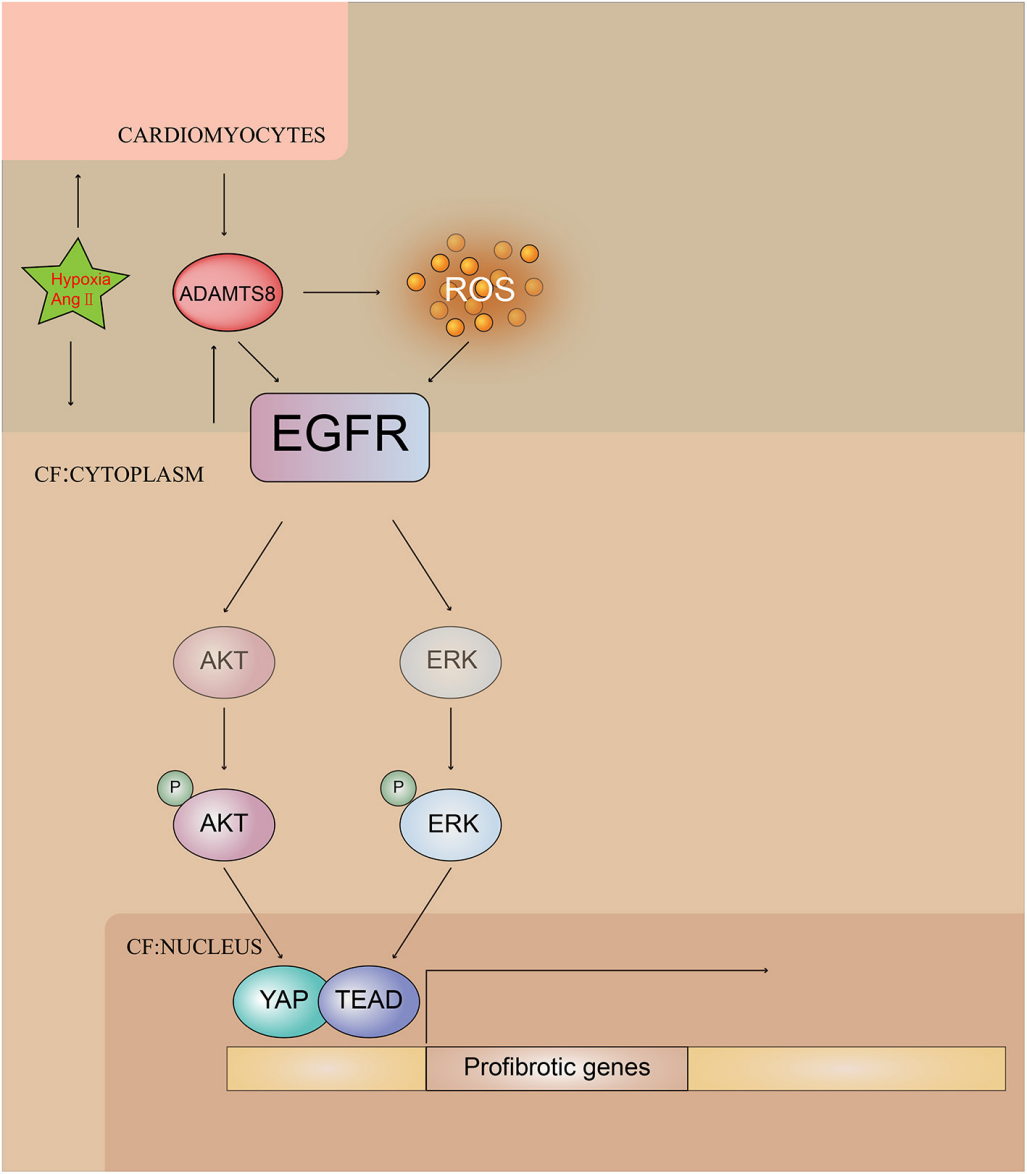
Illustrating ADAMTS8-mediated activation of EGFR signaling pathway by hypoxia and Ang-II stimulation.

Collectively, these results indicated the pivotal involvement of ADAMTS8 in the pathogenesis of cardiac fibrosis and revealed that ADAMTS8 could be a useful target for preventing and treating cardiac fibrosis (Figure 10).

## Discussion

The present study has made some major findings that can be outlined as the following: (1) ADAMTS8 expression was increased in the MI rat model, cardiac fibrosis induced by transverse aortic constriction (TAC), and patients with heart failure, which were all accompanied by myofibroblast activation and collagen fiber deposition in these diseases and models. (2) EGFR and related downstream signaling pathways were partially activated by increased ADAMTS8 secretion, thereby promoting fibroblast activation *in vitro*. (3) ADAMTS8 overexpression i*n vivo* impaired cardiac function and promoted myocardial fibrosis in the MI rat model. (4) Mebendazole treatment reduced ADAMTS8 expression in the cardiac myocytes and fibroblasts and ameliorated heart fibrosis. In conclusion, this study was the first to systematically explore the role of ADAMTS8 and reveal a novel mechanism in cardiac fibrosis.

ADAMTSs are secreted proteins characterized by the presence of an MMP domain and a variable number of TSP-1. It has been reported that various ADAMTSs can regulate cell proliferation, migration, adhesion and intracellular signal transduction (10, 11). For instance, ADAMTS1 deficiency induces thoracic aortic aneurysms and dissections in mice, while it is downregulated in the aorta of patients with Marfan syndrome (36). Moreover, it has been reported that ADAMTS7 deficiency, a novel locus for coronary artery disease in humans, suppressed neointimal formation after wire injury in mice and downregulated the migration of vascular smooth muscle cells (36). It is also known that ADAMTS8 plays a crucial role in antiangiogenic responses. Unlike ADAMTS1 and 7, both ubiquitously expressed in various tissues, ADAMTS8 is specifically expressed in the lung and the heart (14). Presently, only a few reports describe the important role of ADAMTSs in cardiac fibrosis and cardiac hypertrophy. In the transverse aortic constriction (TAC) model, the expression of ADAMTS16 was enhanced, and its overexpression *in vitro* could activate cardiac fibroblasts (12). Another *in vivo* pressure overload mouse model showed that ADAMTS2 was upregulated during cardiac hypertrophy (13). ADAMTS8 is associated with fibroblast activation and organ fibrosis promotion in some diseases. For example, ADAMTS8 expression was significantly enhanced in pulmonary arterial hypertension and linear morphoea, a connective tissue disease. Many studies have reported that ADAMTS8 overexpression induced normal fibroblasts to transform into myofibroblasts (15, 16). In the pulmonary hypertension rat model induced by the Sugen/hypoxia model, the levels of ADAMTS8 in the lung and RV were significantly elevated, and cardiomyocyte-specific ADAMTS8 knockout mice showed significantly less RV fibrosis than the control mice after chronic hypoxia (15). Considering all the findings above, we hypothesized that the ADAMTS8 expression might also be associated with the development of cardiac fibrosis. Here, our results provided evidence that ADAMTS8 expression was increased in the MI rat model, cardiac fibrosis induced by transverse aortic constriction (TAC), and DCM patients with severe cardiac fibrosis.

ADAMTS8 overexpression in MI rats impaired cardiac function and promoted myocardial fibrosis at 28 days post-MI *in vivo*. *In vitro* studies revealed that overexpression of ADAMTS8 promoted cardiac fibroblast transition into myofibroblasts and induced cardiac fibroblast proliferation, migration and collagen synthesis. Therefore, the combined findings *in vivo* and *in vitro* indicated that the ADAMTS8 overexpression in the infarct boundary zone after myocardial infarction was one of the reasons for CFs enhancement, which lead to cardiac dysfunction and remodeling after myocardial infarction. Thus, targeting ADAMTS8 might be a potential treatment for cardiac fibrosis.

Changes in the mitochondrial ROS (mtROS) generation and oxidative phosphorylation are essential for myofibroblast formation (37). In lung fibroblasts, TGFβ-induced mitochondrial ROS generation transcriptionally upregulated NOX4, potentially as a mean to sustain elevated intracellular ROS levels to activate the AKT and MAPK pathways (27). A study has reported that the knockdown of NOX4 inhibited the induction of SMA and FN-EDA *via* ERK1/2 signaling in the kidney (38). Previous studies have shown that canonical Samd signaling pathways and non-canonical signaling pathways such as MAPK and PI3K/Akt are associated with cardiac fibrosis (39–41). ADAMTS8 knockdown in pulmonary artery smooth muscle cells (PASMCs) showed significantly lower NADPH oxidase activity and ROS levels after hypoxia. ADAMTS8 cell-specific knockout in the pulmonary artery smooth muscle of mice had significantly lower pulmonary artery ROS levels than control mice. ADAMTS8 also regulated the balance between mitochondrial fission and fusion in PASMCs (15). *In vitro* studies demonstrated that ADAMTS8 enhanced NOX4-mediated ROS production and CFs proliferation. Furthermore, ADAMTS8 knockdown in CFs significantly increased the phosphorylation level of mitochondrial division-related protein, DRP-1, at Ser637. Overexpression of ADAMTS8 in fibroblasts activated AKT and MAPK signaling pathways, and the activation of these pathways was inhibited by ADAMTS8 knockdown; the same results were also proved *in vivo*.

Proteoglycans which are crucial components of ECM, can be degraded by ADAMTS8 (23). ECM can sequester and locally release growth factors, such as epidermal growth factor (EGF), which can act as soluble ligands binding to EGFR, affecting cell proliferation and migration (31). It is known that G-protein coupled receptors induces EGFR transactivation, which works through intracellular kinases such as disintegrin and metalloproteases (ADAMs), cleaving EGFR ligands into soluble active moieties that activate EGFR. This sequence leads to the subsequent activation of downstream signaling pathways, including the MAPK and Akt (32). In addition, the MAPK and Akt signaling pathways dependent on EGFR have been revealed to be involved in pulmonary and hepatic fibrosis, among other organs fibrosis (42–44). Our study results indicated that activating cardiac fibroblasts induced by ADAMTS8 is mediated *via* activating the EGFR signaling pathway.

Several studies in Drosophila and diabetic renal interstitial fibrogenesis have suggested that activation of the MAPK and AKT pathways led to YAP activation (33, 34), and EGFR was the upstream of these two pathways. Cardiac fibrosis was attenuated by blockade of fibroblast YAP after myocardial infarction (35). We found that YAP activation and subsequent phosphorylation of YAP induced by ADAMTS8 overexpression were related to cardiac fibrosis characterized by myofibroblast activation. Inhibition of YAP significantly reversed ADAMTS8-induced Collage1 and α-SMA expression levels, revealing that ADAMTS8 regulates the cardiac fibrotic process by activating the EGFR-MAPK/AKT-YAP signaling pathway. Additionally, we found that mebendazole, which is used to treat parasite infections, suppressed the expression of ADAMTS8 and ameliorated cardiac fibrosis induced by MI and TAC. Thus, mebendazole may be a promising agent, and the possibility of drug repositioning for use in patients with heart failure needs to be further explored.

## Data Availability Statement

The raw data supporting the conclusions of this article will be made available by the authors, without undue reservation.

## Ethics Statement

The studies involving human participants were reviewed and approved by Medical Ethics Committee of Xinhua Hospital Affiliated to Shanghai Jiaotong University School of Medicine. The patients/participants provided their written informed consent to participate in this study. The animal study was reviewed and approved by Medical Ethics Committee of Xinhua Hospital Affiliated to Shanghai Jiaotong University School of Medicine.

## Author Contributions

All authors listed have made a substantial, direct, and intellectual contribution to the work and approved it for publication.

## Funding

This study was supported by the Nature Science Foundation of China (grant no: 81974295).

## Conflict of Interest

The authors declare that the research was conducted in the absence of any commercial or financial relationships that could be construed as a potential conflict of interest.

## Publisher's Note

All claims expressed in this article are solely those of the authors and do not necessarily represent those of their affiliated organizations, or those of the publisher, the editors and the reviewers. Any product that may be evaluated in this article, or claim that may be made by its manufacturer, is not guaranteed or endorsed by the publisher.

## Abbreviations

ADAMTS8: A disintegrin and metalloproteinase with thrombospondin motifs 8
CFs: Cardiac fibroblasts
TAC: Transverse aortic constriction
ECM: Extracellular matrix
MI: Myocardial infarction
DCM: Dilated cardiomyopathy
LAD: Left anterior descending
PASMC: Pulmonary artery smooth muscle cell
EF: Ejection fraction
α-SMA: Alpha smooth muscle actin
EGFR: Epidermal growth factor
CM: Conditioned Medium.

## References

[1] Su SA Yang D Wu Y Xie Y Zhu W Cai Z. EphrinB2 regulates cardiac fibrosis through modulating the interaction of stat3 and TGF-β/Smad3 signaling. Circ Res. (2017) 121:617–27. 10.1161/CIRCRESAHA.117.311045 28743805

[2] Bonnans C Chou J Werb Z. Remodelling the extracellular matrix in development and disease. Nat Rev Mol Cell Biol. (2014) 15:786–801. 10.1038/nrm3904 25415508 PMC4316204

[3] Rai V Sharma P Agrawal S Agrawal DK. Relevance of mouse models of cardiac fibrosis and hypertrophy in cardiac research. Mol Cell Biochem. (2017) 424:123–45. 10.1007/s11010-016-2849-0 27766529 PMC5219849

[4] Frangogiannis NG. Cardiac fibrosis: cell biological mechanisms, molecular pathways and therapeutic opportunities. Mol Aspects Med. (2019) 65:70–99. 10.1016/j.mam.2018.07.001 30056242

[5] Pellman J Zhang J Sheikh F. Myocyte-fibroblast communication in cardiac fibrosis and arrhythmias: mechanisms and model systems. J Mol Cell Cardiol. (2016) 94:22–31. 10.1016/j.yjmcc.2016.03.005 26996756 PMC4861678

[6] van den Borne SW Diez J Blankesteijn WM Verjans J Hofstra L Narula J. Myocardial remodeling after infarction: the role of myofibroblasts. Nat Rev Cardiol. (2010) 7:30–7. 10.1038/nrcardio.2009.199 19949426

[7] Weber KT Brilla CG. Pathological hypertrophy and cardiac interstitium. Fibrosis and renin-angiotensin-aldosterone system. Circulation. (1991) 83:1849–65. 10.1161/01.CIR.83.6.1849 1828192

[8] Weber KT Sun Y Bhattacharya SK Ahokas RA Gerling IC. Myofibroblast-mediated mechanisms of pathological remodelling of the heart. Nat Rev Cardiol. (2013) 10:15–26. 10.1038/nrcardio.2012.158 23207731

[9] Kanisicak O Khalil H Ivey MJ Karch J Maliken BD Correll RN. Genetic lineage tracing defines myofibroblast origin and function in the injured heart. Nat Commun. (2016) 7:12260. 10.1038/ncomms12260 27447449 PMC5512625

[10] Rodríguez-Manzaneque JC Fernández-Rodríguez R Rodríguez-Baena FJ Iruela-Arispe ML. ADAMTS proteases in vascular biology. Matrix Biol. (2015) 44–6:38–45. 10.1016/j.matbio.2015.02.004 25698314 PMC8086761

[11] Rocks N Paulissen G El Hour M Quesada F Crahay C Gueders M. Emerging roles of ADAM and ADAMTS metalloproteinases in cancer. Biochimie. (2008) 90:369–79. 10.1016/j.biochi.2007.08.008 17920749

[12] Yao Y Hu C Song Q Li Y Da X Yu Y. ADAMTS16 activates latent TGF-β, accentuating fibrosis and dysfunction of the pressure-overloaded heart. Cardiovasc Res. (2020) 116:956–69. 10.1093/cvr/cvz187 31297506 PMC7868664

[13] Wang X Chen W Zhang J Khan A Li L Huang F. Critical role of ADAMTS2 (a disintegrin and metalloproteinase with thrombospondin motifs 2) in cardiac hypertrophy induced by pressure overload. Hypertension. (2017) 69:1060–9. 10.1161/HYPERTENSIONAHA.116.08581 28373586

[14] Vázquez F Hastings G Ortega MA Lane TF Oikemus S Lombardo M. METH-1, a human ortholog of ADAMTS-1, and METH-2 are members of a new family of proteins with angio-inhibitory activity. J Biol Chem. (1999) 274:23349–57. 10.1074/jbc.274.33.23349 10438512

[15] Omura J Satoh K Kikuchi N Satoh T Kurosawa R Nogi M. ADAMTS8 promotes the development of pulmonary arterial hypertension and right ventricular failure: a possible novel therapeutic target. Circ Res. (2019) 125:884–906. 10.1161/CIRCRESAHA.119.315398 31556812

[16] Badshah II Brown S Weibel L Rose A Way B Sebire N. Differential expression of secreted factors SOSTDC1 and ADAMTS8 cause profibrotic changes in linear morphoea fibroblasts. Br J Dermatol. (2019) 180:1135–49. 10.1111/bjd.17352 30367460

[17] Zhang L Gan ZK Han LN Wang H Bai J Tan GJ Li XX. Protective effect of heme oxygenase-1 on Wistar rats with heart failure through the inhibition of inflammation and amelioration of intestinal microcirculation. J Geriatr Cardiol. (2015) 12:353–65. 10.11909/j.issn.1671-5411.2015.04.001 26346675 PMC4554778

[18] Sorokina N O'Donnell JM McKinney RD Pound KM Woldegiorgis G LaNoue KF. Recruitment of compensatory pathways to sustain oxidative flux with reduced carnitine palmitoyltransferase I activity characterizes inefficiency in energy metabolism in hypertrophied hearts. Circulation. (2007) 115:2033–41. 10.1161/CIRCULATIONAHA.106.668665 17404155

[19] Hong T Wei Y Xue X Li Y Dong H Guo X. Novel anti-coagulative nanocomplex in delivering miRNA-1 inhibitor against microvascular obstruction of myocardial infarction. Adv Healthc Mater. (2020) 9:e1901783. 10.1002/adhm.201901783 32338452

[20] Ge Z Chen Y Wang B Zhang X Yan Y Zhou L. MFGE8 attenuates Ang-II-induced atrial fibrosis and vulnerability to atrial fibrillation through inhibition of TGF-β1/Smad2/3 pathway. J Mol Cell Cardiol. (2020) 139:164–75. 10.1016/j.yjmcc.2020.01.001 31958465

[21] Wang Q Yu Y Zhang P Chen Y Li C Chen J. The crucial role of activin A/ALK4 pathway in the pathogenesis of Ang-II-induced atrial fibrosis and vulnerability to atrial fibrillation. Basic Res Cardiol. (2017) 112:47. 10.1007/s00395-017-0634-1 28639003

[22] Yang LC Zhang PP Chen XM Li CY Sun J Hou JW Chen RH. Semaphorin 3a transfection into the left stellate ganglion reduces susceptibility to ventricular arrhythmias after myocardial infarction in rats. Europace. (2016) 18:1886–96. 10.1093/europace/euv276 26541708

[23] Collins-Racie LA Flannery CR Zeng W Corcoran C Annis-Freeman B Agostino MJ. ADAMTS-8 exhibits aggrecanase activity and is expressed in human articular cartilage. Matrix Biol. (2004) 23:219–30. 10.1016/j.matbio.2004.05.004 15296936

[24] Baudino TA Carver W Giles W Borg TK. Cardiac fibroblasts: friend or foe? Am J Physiol Heart Circ Physiol. (2006) 291:H1015–26. 10.1152/ajpheart.00023.2006 16617141

[25] Huang G Cong Z Wang X Yuan Y Xu R Lu Z. Targeting HSP90 attenuates angiotensin II-induced adventitial remodelling via suppression of mitochondrial fission. Cardiovasc Res. (2020) 116:1071–84. 10.1093/cvr/cvz194 31346611

[26] Lu H Tian A Wu J Yang C Xing R Jia P. Danshensu inhibits β-adrenergic receptors-mediated cardiac fibrosis by ROS/p38 MAPK axis. Biol Pharm Bull. (2014) 37:961–7. 10.1248/bpb.b13-00921 24882408

[27] Jain M Rivera S Monclus EA Synenki L Zirk A Eisenbart J. Mitochondrial reactive oxygen species regulate transforming growth factor-β signaling. J Biol Chem. (2013) 288:770–7. 10.1074/jbc.M112.431973 23204521 PMC3543026

[28] Chang CR Blackstone C. Cyclic AMP-dependent protein kinase phosphorylation of Drp1 regulates its GTPase activity and mitochondrial morphology. J Biol Chem. (2007) 282:21583–7. 10.1074/jbc.C700083200 17553808

[29] Liu RM Choi J Wu JH Gaston Pravia KA Lewis KM Brand JD. Oxidative modification of nuclear mitogen-activated protein kinase phosphatase 1 is involved in transforming growth factor beta1-induced expression of plasminogen activator inhibitor 1 in fibroblasts. J Biol Chem. (2010) 285:16239–47. 10.1074/jbc.M110.111732 20228065 PMC2871491

[30] Anilkumar N San Jose G Sawyer I Santos CX Sand C Brewer AC. 28-kDa splice variant of NADPH oxidase-4 is nuclear-localized and involved in redox signaling in vascular cells. Arterioscler Thromb Vasc Biol. (2013) 33:e104–12. 10.1161/ATVBAHA.112.300956 23393389

[31] Hynes RO. The extracellular matrix: not just pretty fibrils. Science. (2009) 326:1216–9. 10.1126/science.1176009 19965464 PMC3536535

[32] Tang J Liu N Zhuang S. Role of epidermal growth factor receptor in acute and chronic kidney injury. Kidney Int. (2013) 83:804–10. 10.1038/ki.2012.435 23325080 PMC4626012

[33] Reddy BV Irvine KD. Regulation of Hippo signaling by EGFR-MAPK signaling through Ajuba family proteins. Dev Cell. (2013) 24:459–71. 10.1016/j.devcel.2013.01.020 23484853 PMC3624988

[34] Chen J Harris RC. Interaction of the EGF receptor and the hippo pathway in the diabetic kidney. J Am Soc Nephrol. (2016) 27:1689–700. 10.1681/ASN.2015040415 26453611 PMC4884112

[35] Francisco J Zhang Y Jeong JI Mizushima W Ikeda S Ivessa A. Blockade of fibroblast YAP attenuates cardiac fibrosis and dysfunction through MRTF-A inhibition. JACC Basic Transl Sci. (2020) 5:931–45. 10.1016/j.jacbts.2020.07.009 33015415 PMC7524792

[36] Oller J Méndez-Barbero N Ruiz EJ Villahoz S Renard M Canelas LI. Nitric oxide mediates aortic disease in mice deficient in the metalloprotease Adamts1 and in a mouse model of Marfan syndrome. Nat Med. (2017) 23:200–12. 10.1038/nm.4266 28067899

[37] Gibb AA Lazaropoulos MP Elrod JW. Myofibroblasts and fibrosis: mitochondrial and metabolic control of cellular differentiation. Circ Res. (2020) 127:427–47. 10.1161/CIRCRESAHA.120.316958 32673537 PMC7982967

[38] Bondi CD Manickam N Lee DY Block K Gorin Y Abboud HE. NAD(P)H oxidase mediates TGF-beta1-induced activation of kidney myofibroblasts. J Am Soc Nephrol. (2010) 21:93–102. 10.1681/ASN.2009020146 19926889 PMC2799274

[39] Derynck R Zhang YE. Smad-dependent and Smad-independent pathways in TGF-beta family signalling. Nature. (2003) 425:577–84. 10.1038/nature02006 14534577

[40] Gruhle S Sauter M Szalay G Ettischer N Kandolf R Klingel K. The prostacyclin agonist iloprost aggravates fibrosis and enhances viral replication in enteroviral myocarditis by modulation of ERK signaling and increase of iNOS expression. Basic Res Cardiol. (2012) 107:287. 10.1007/s00395-012-0287-z 22836587

[41] Kang HR Cho SJ Lee CG Homer RJ Elias JA. Transforming growth factor (TGF)-beta1 stimulates pulmonary fibrosis and inflammation *via* a Bax-dependent, bid-activated pathway that involves matrix metalloproteinase-12. J Biol Chem. (2007) 282:7723–32. 10.1074/jbc.M610764200 17209037

[42] Ai W Zhang Y Tang QZ Yan L Bian ZY Liu C. Silibinin attenuates cardiac hypertrophy and fibrosis through blocking EGFR-dependent signaling. J Cell Biochem. (2010) 110:1111–22. 10.1002/jcb.22623 20564207

[43] Fuchs BC Hoshida Y Fujii T Wei L Yamada S Lauwers GY. Epidermal growth factor receptor inhibition attenuates liver fibrosis and development of hepatocellular carcinoma. Hepatology. (2014) 59:1577–90. 10.1002/hep.26898 24677197 PMC4086837

[44] Zhou Y Lee JY Lee CM Cho WK Kang MJ Koff JL. Amphiregulin, an epidermal growth factor receptor ligand, plays an essential role in the pathogenesis of transforming growth factor-β-induced pulmonary fibrosis. J Biol Chem. (2012) 287:41991–2000. 10.1074/jbc.M112.356824 23086930 PMC3516745

