# Supplementary figures and images for: ADAMTS8 Promotes Cardiac Fibrosis Partly Through Activating EGFR Dependent Pathway
Source: Front Cardiovasc Med. 2022 Feb 10;9:797137. doi: 10.3389/fcvm.2022.797137 (PMC8866452; doi:10.3389/fcvm.2022.797137)

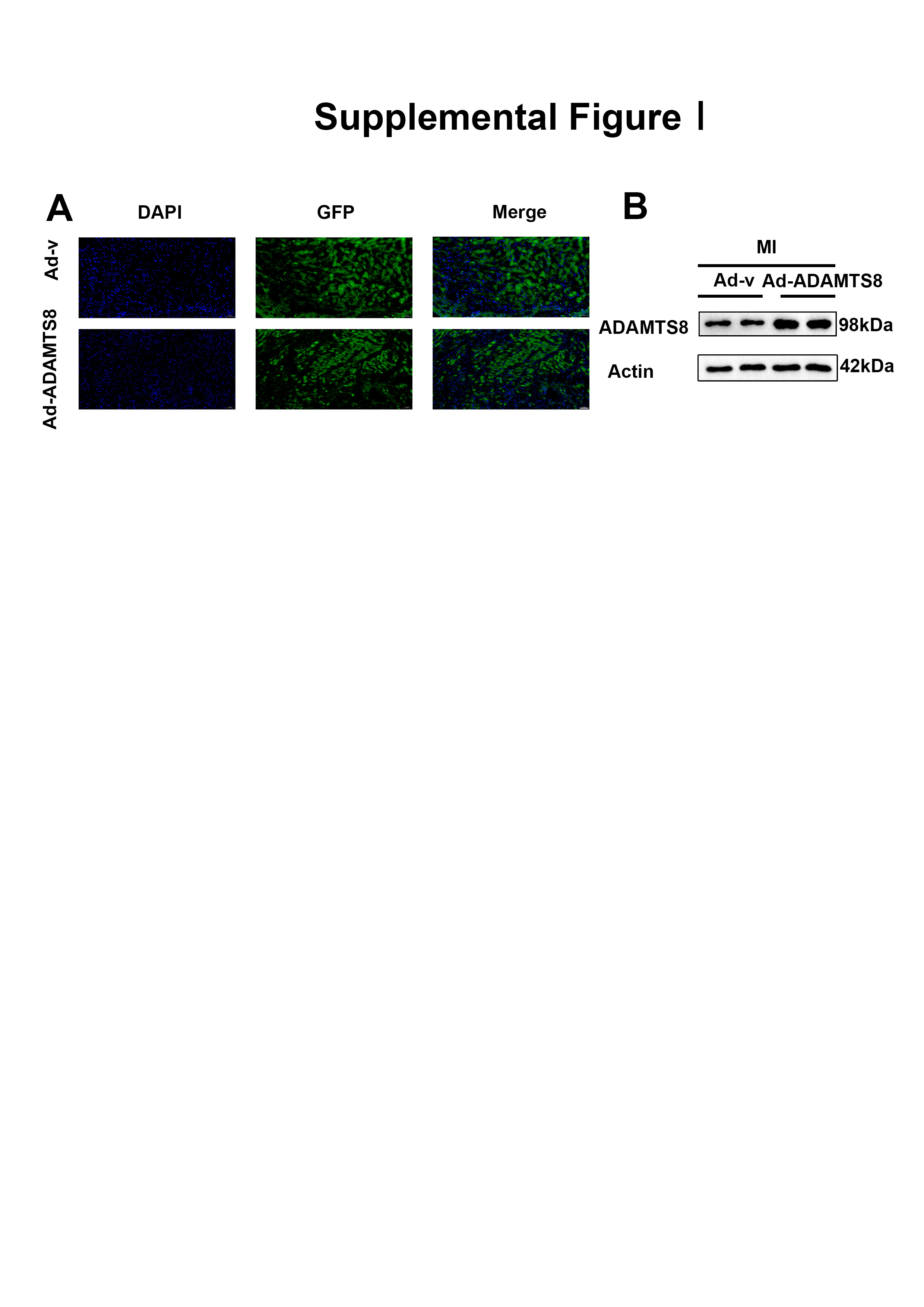

Supplement: Supplementary file 3 [file Image_1.jpg]

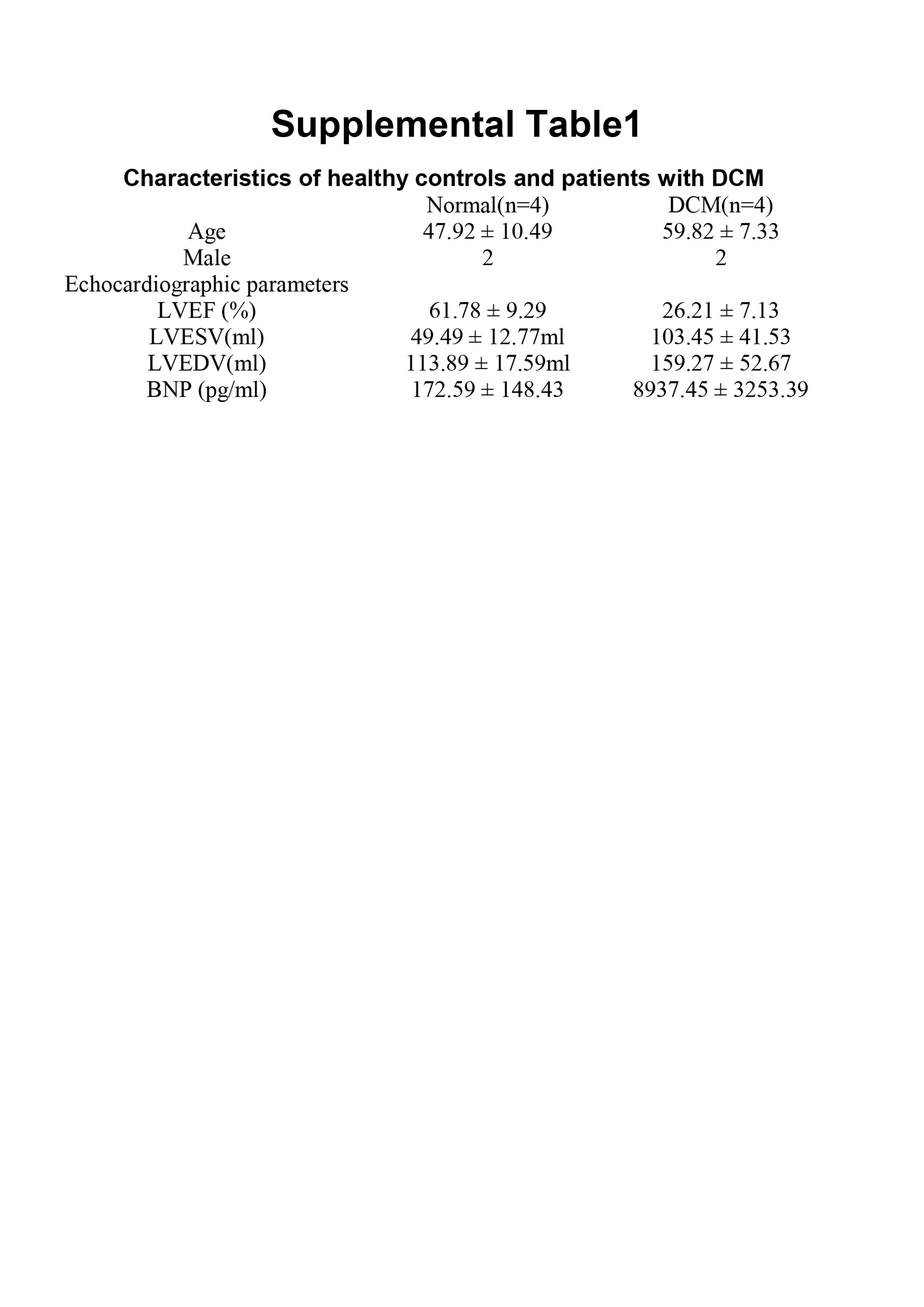

Supplement: Supplementary file 4 [file Image_2.jpg]

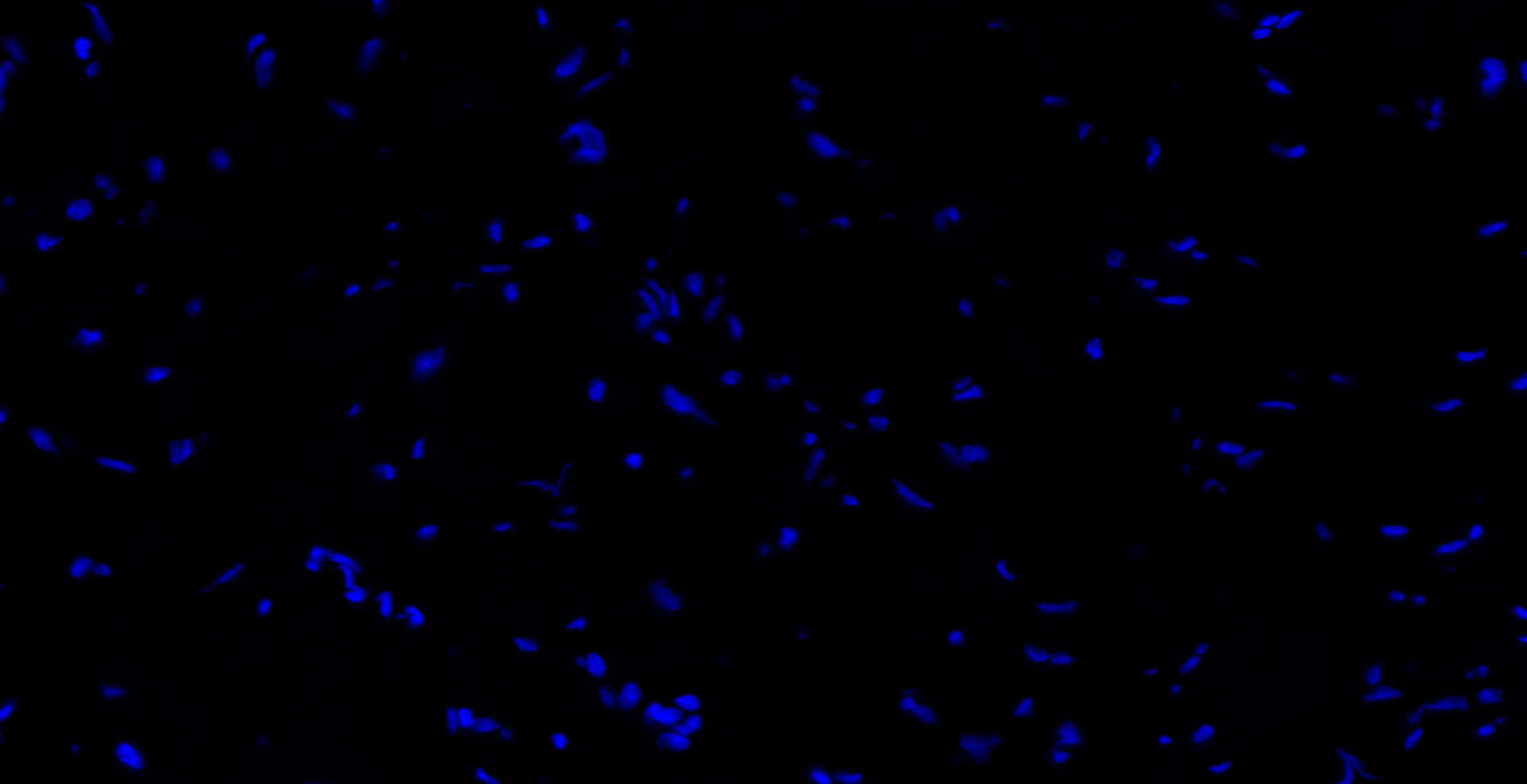

Supplement: Supplementary file 5 [file Data_Sheet_1.ZIP › Figure1 E DCM DAPI.jpg]

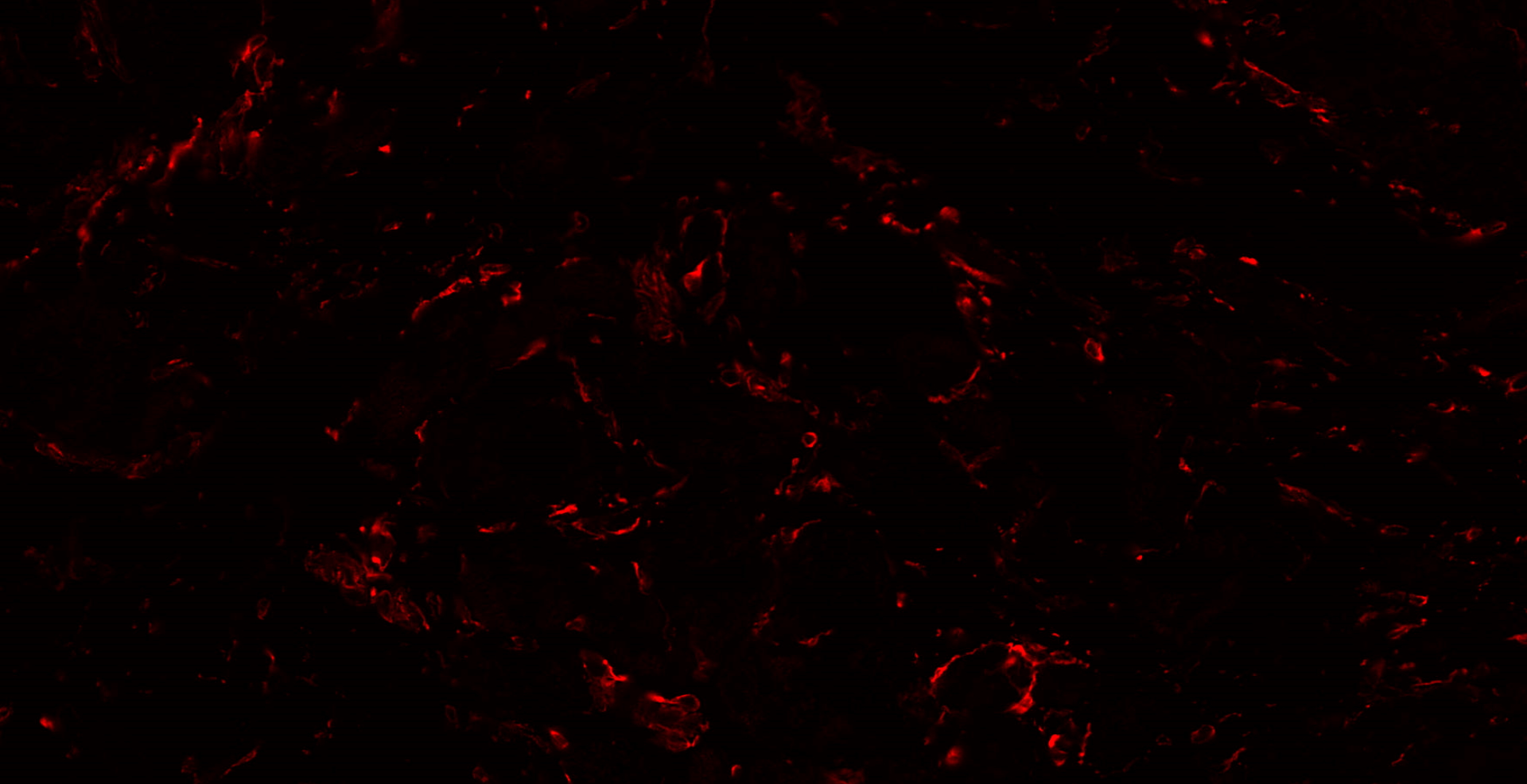

Supplement: Supplementary file 5 [file Data_Sheet_1.ZIP › Figure1 E DCM Vimentin.jpg]

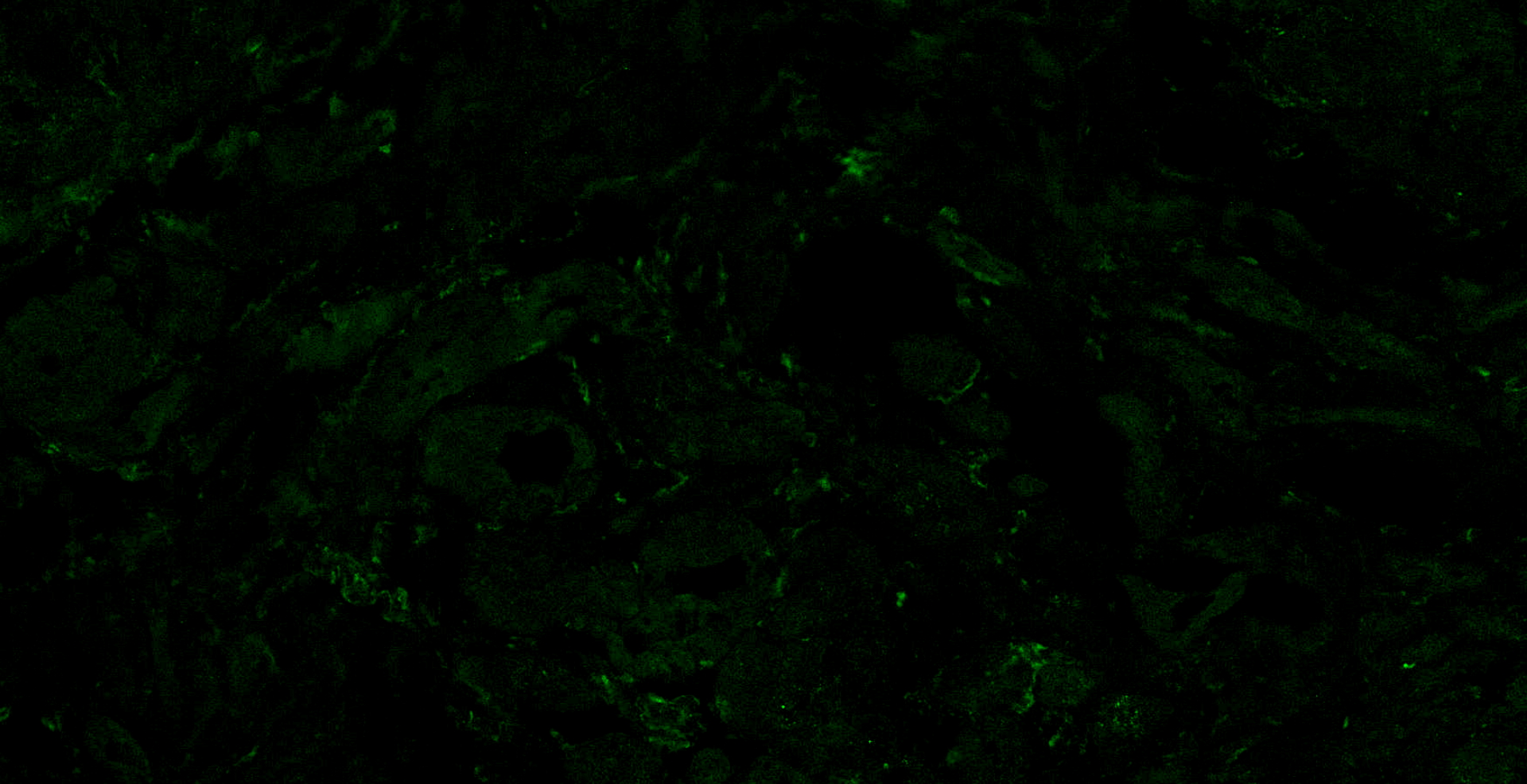

Supplement: Supplementary file 5 [file Data_Sheet_1.ZIP › Figure1 E DCM ADAMTS8.jpg]

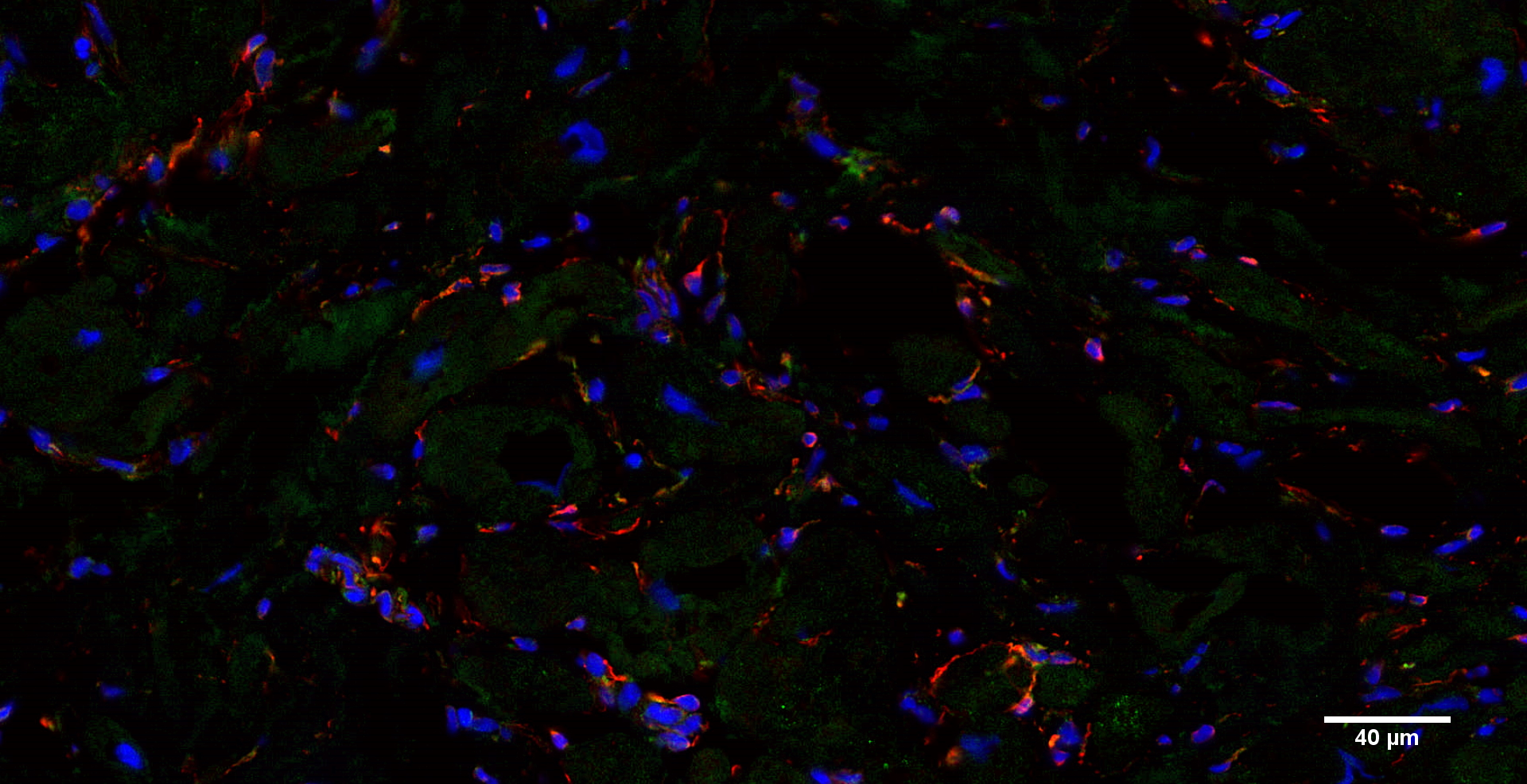

Supplement: Supplementary file 5 [file Data_Sheet_1.ZIP › Figure1 E DCM Merge.tif]

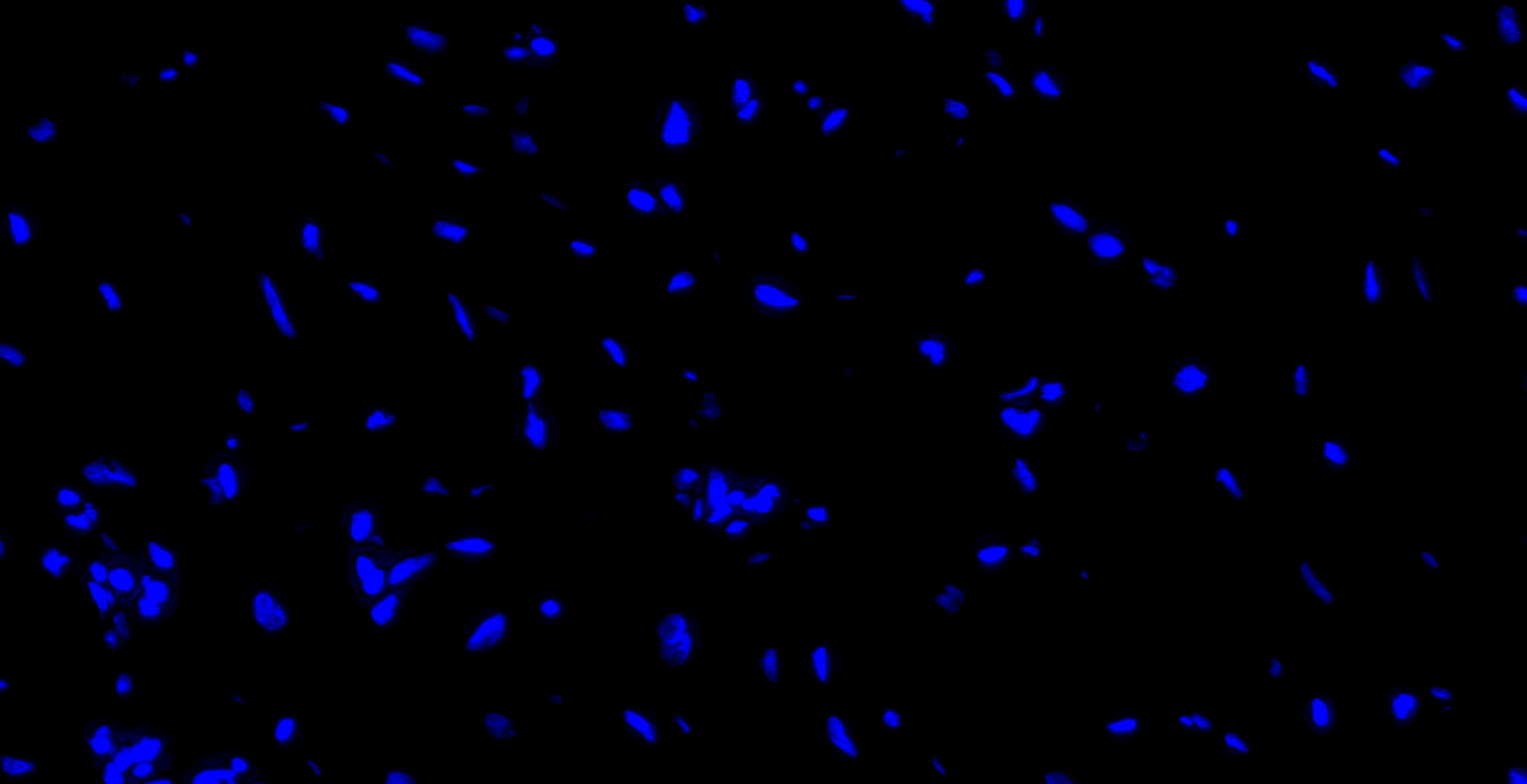

Supplement: Supplementary file 5 [file Data_Sheet_1.ZIP › Figure1 E normal DAPI.jpg]

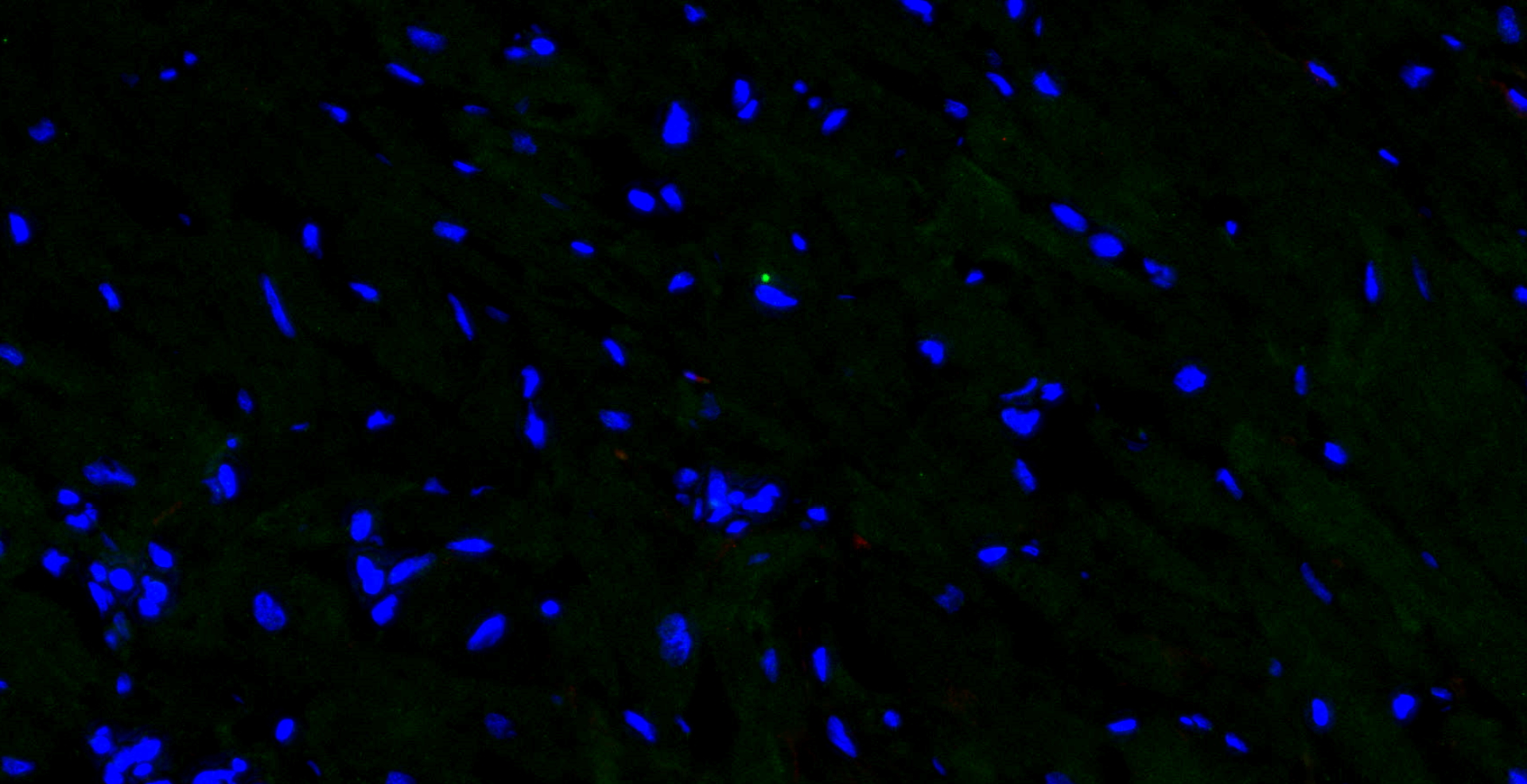

Supplement: Supplementary file 5 [file Data_Sheet_1.ZIP › Figure1 E normal MERGE.jpg]

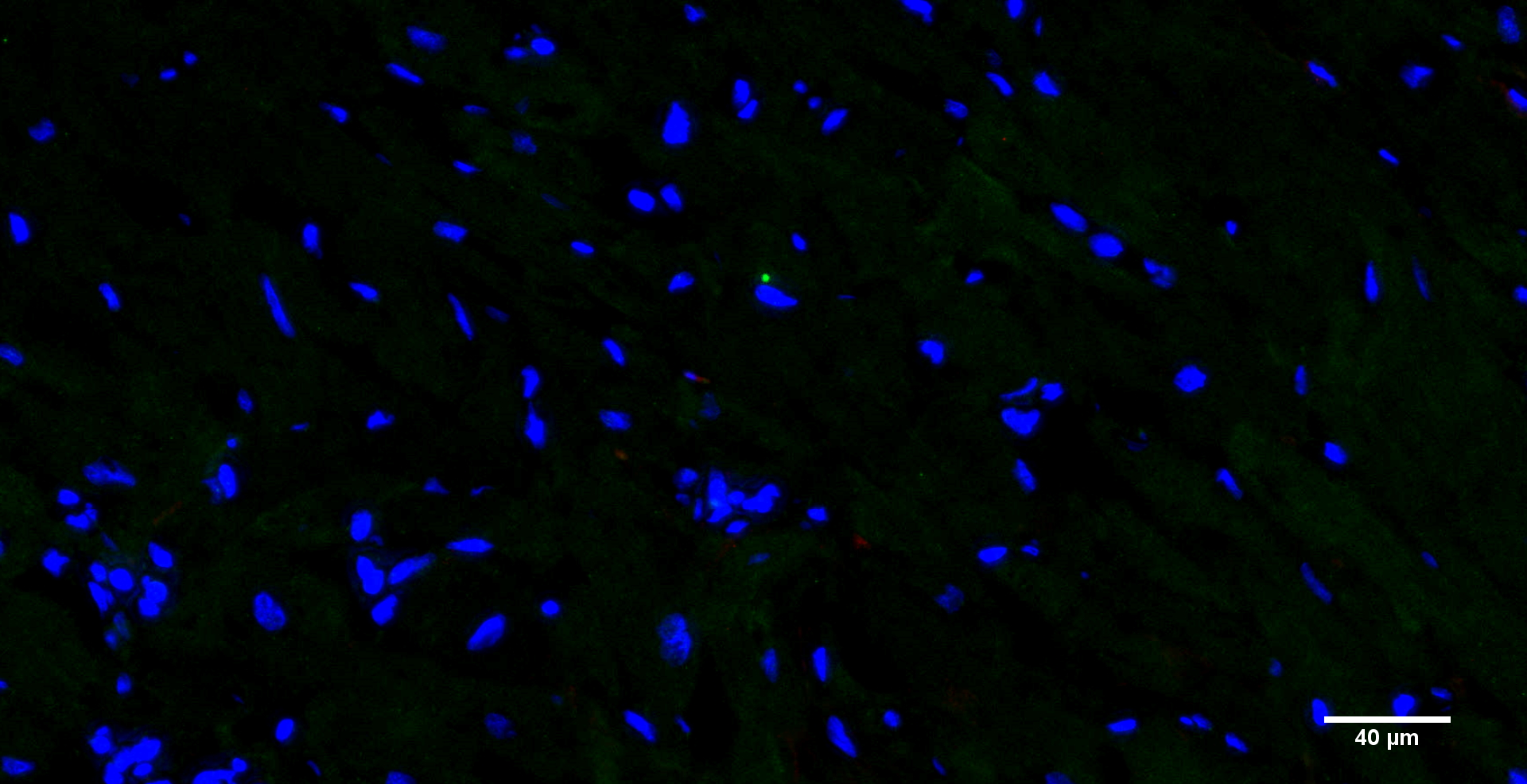

Supplement: Supplementary file 5 [file Data_Sheet_1.ZIP › Figure1 E normal MERGE.tif]

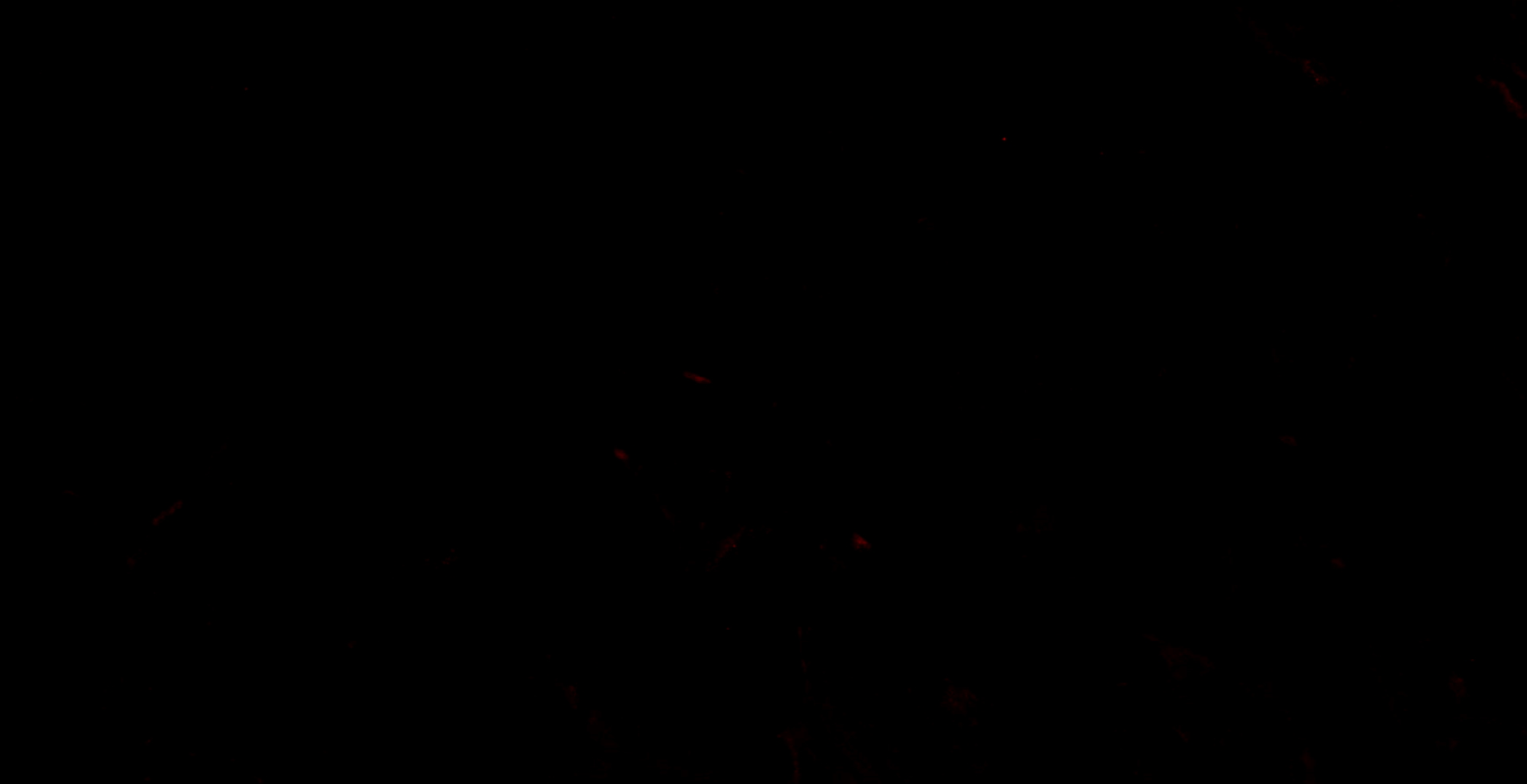

Supplement: Supplementary file 5 [file Data_Sheet_1.ZIP › Figure1 E normal Vimentin.jpg]

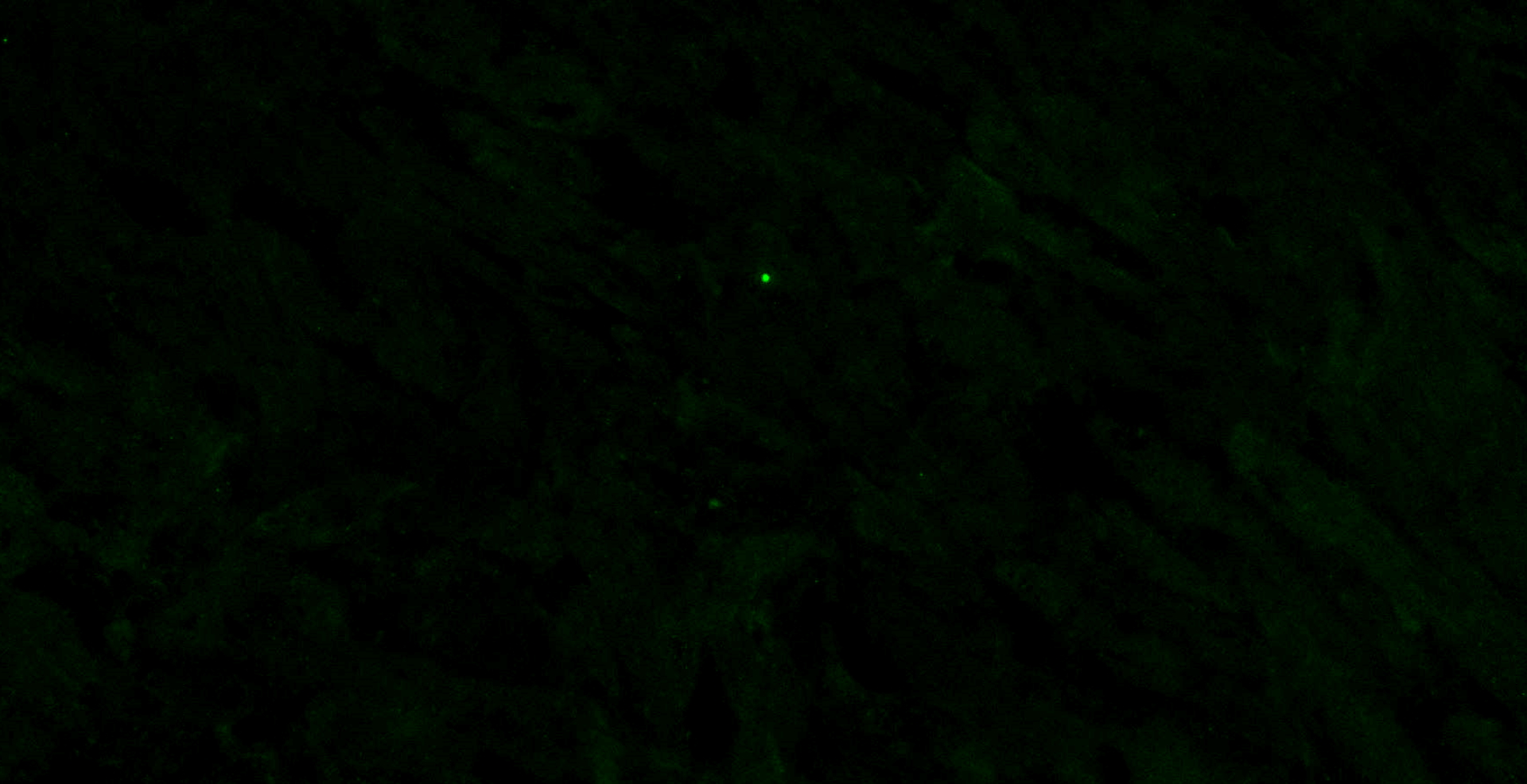

Supplement: Supplementary file 5 [file Data_Sheet_1.ZIP › Figure1 E normal ADAMTS8.jpg]

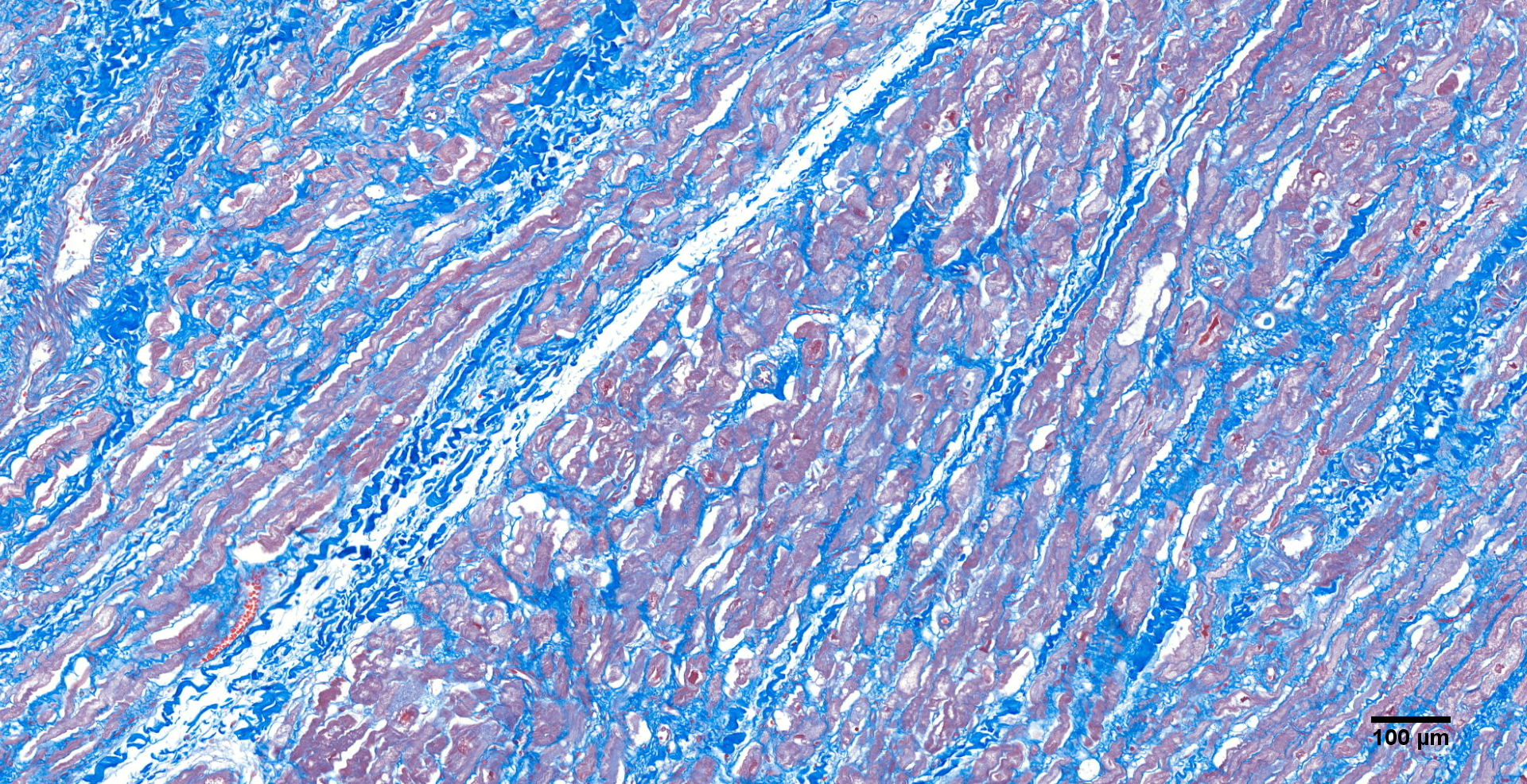

Supplement: Supplementary file 5 [file Data_Sheet_1.ZIP › Figure1A DCM.tif]

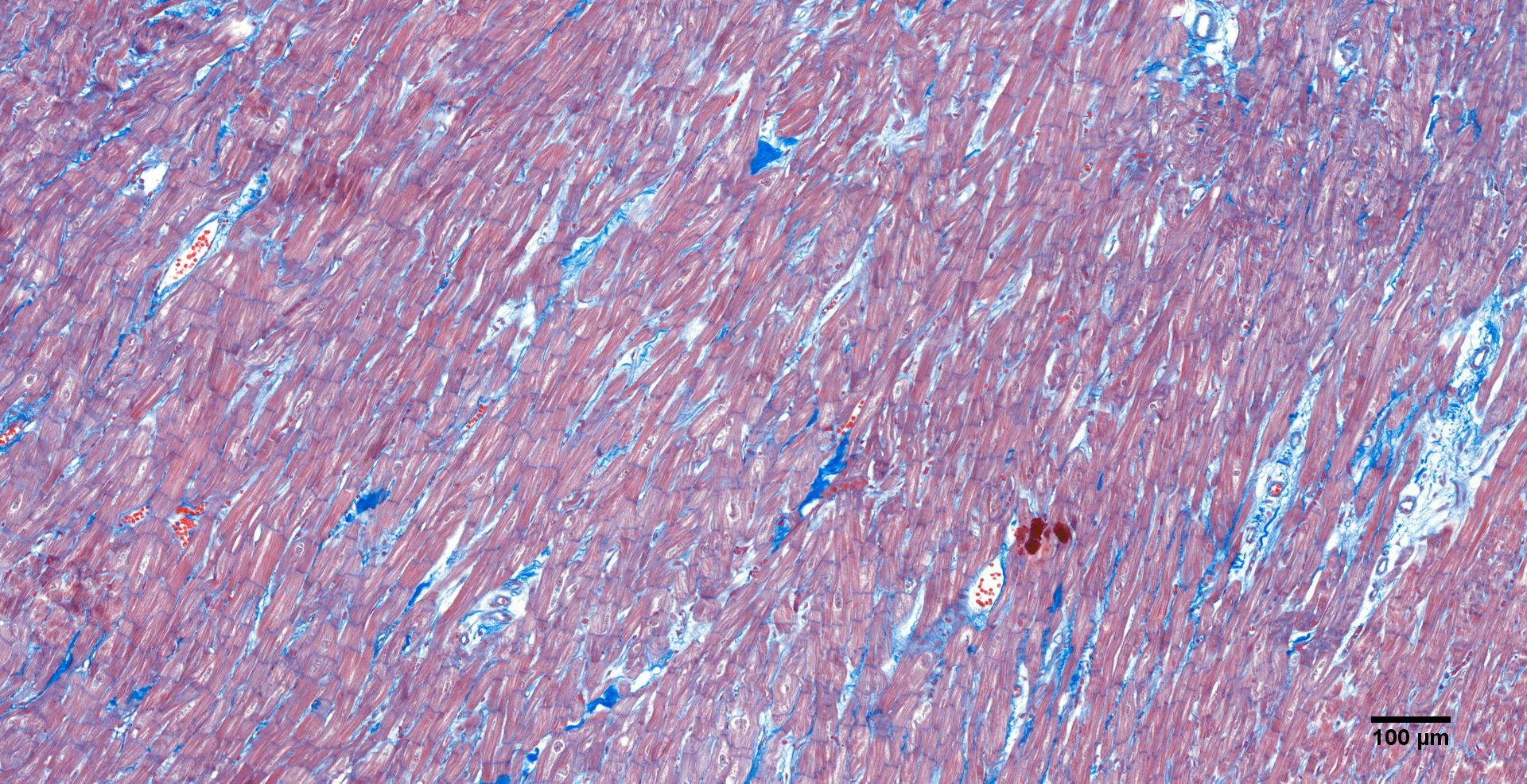

Supplement: Supplementary file 5 [file Data_Sheet_1.ZIP › Figure1A Normal.tif]

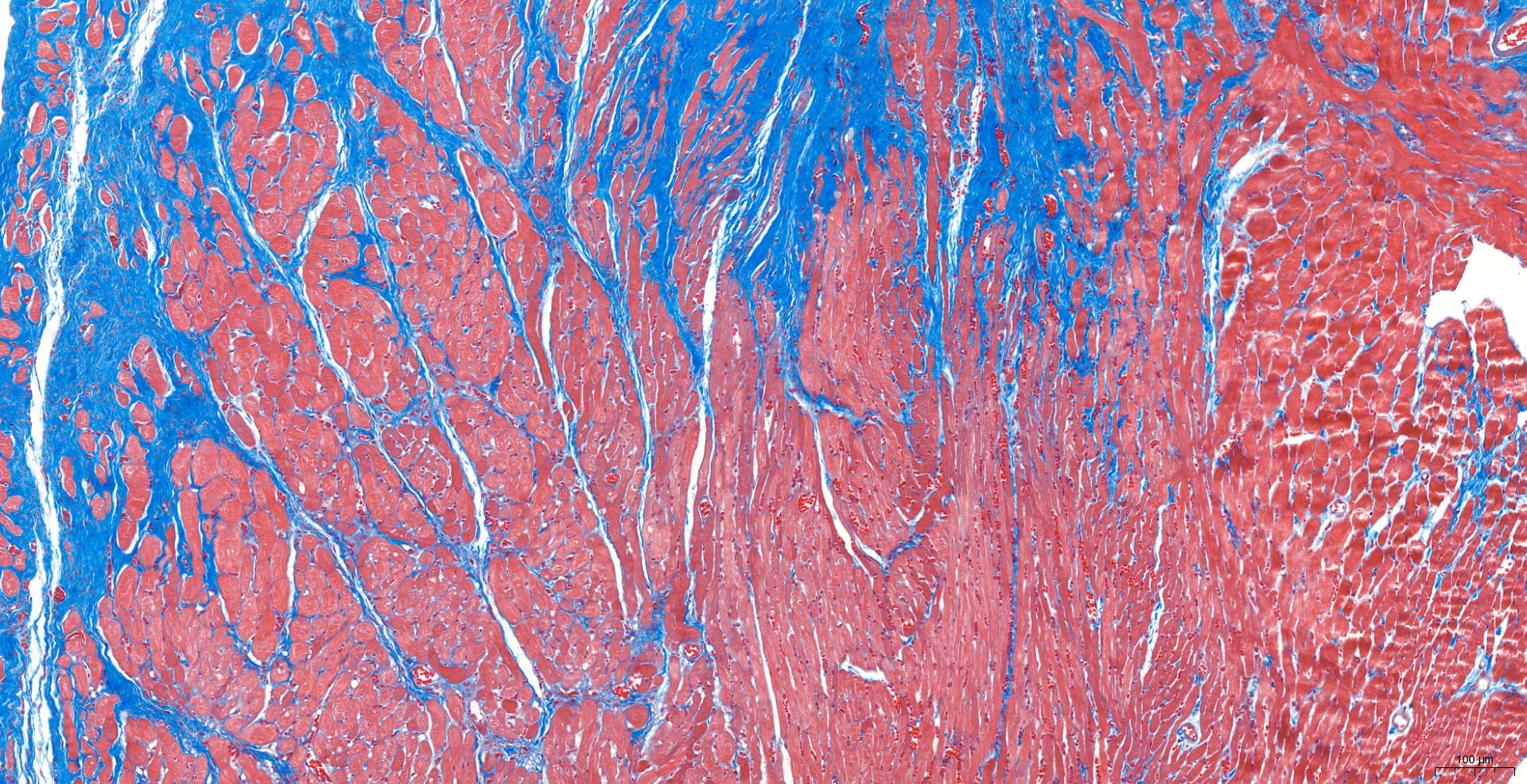

Supplement: Supplementary file 6 [file Data_Sheet_2.ZIP › Figure2 A boder.jpg]

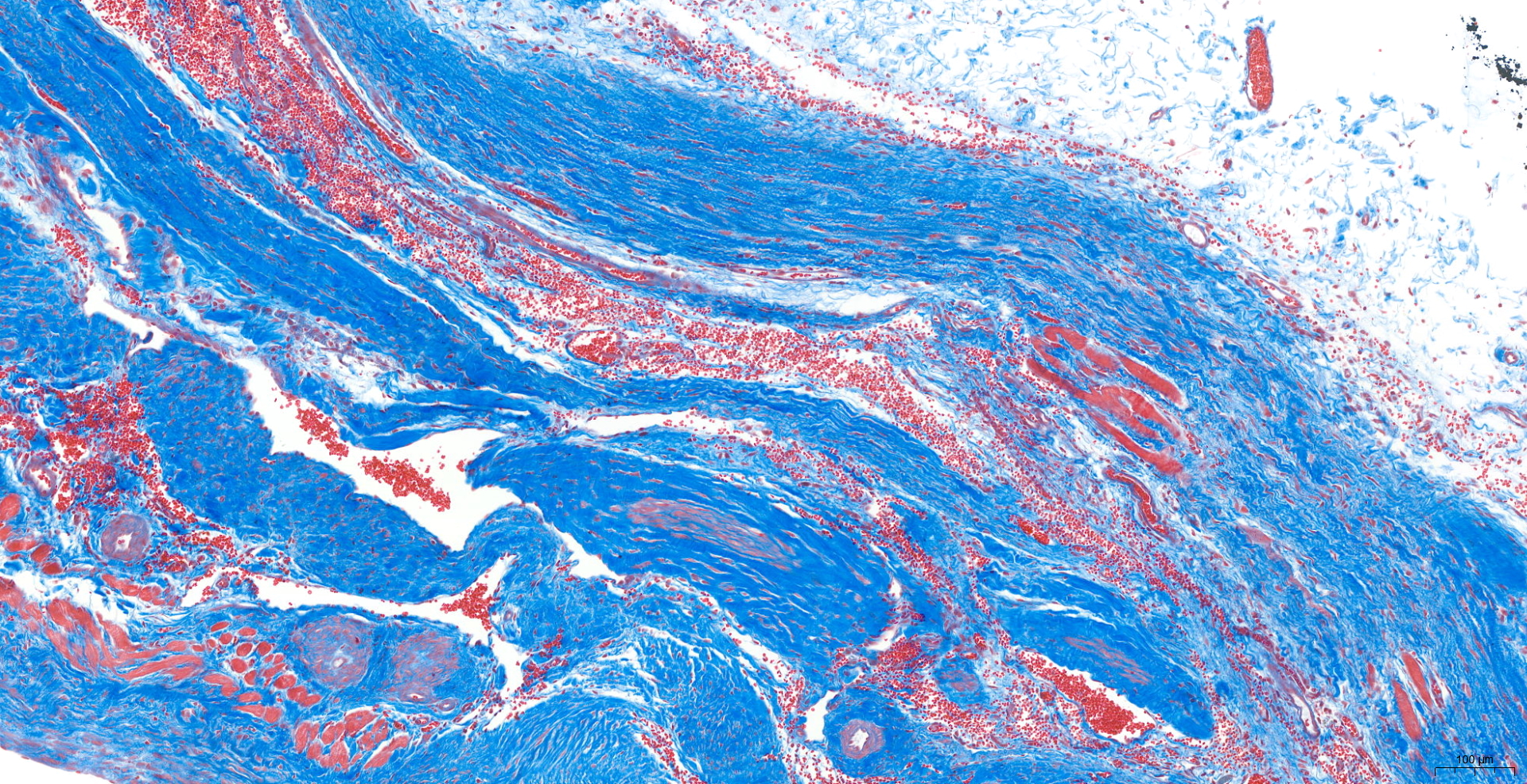

Supplement: Supplementary file 6 [file Data_Sheet_2.ZIP › Figure2 A Infarct.jpg]

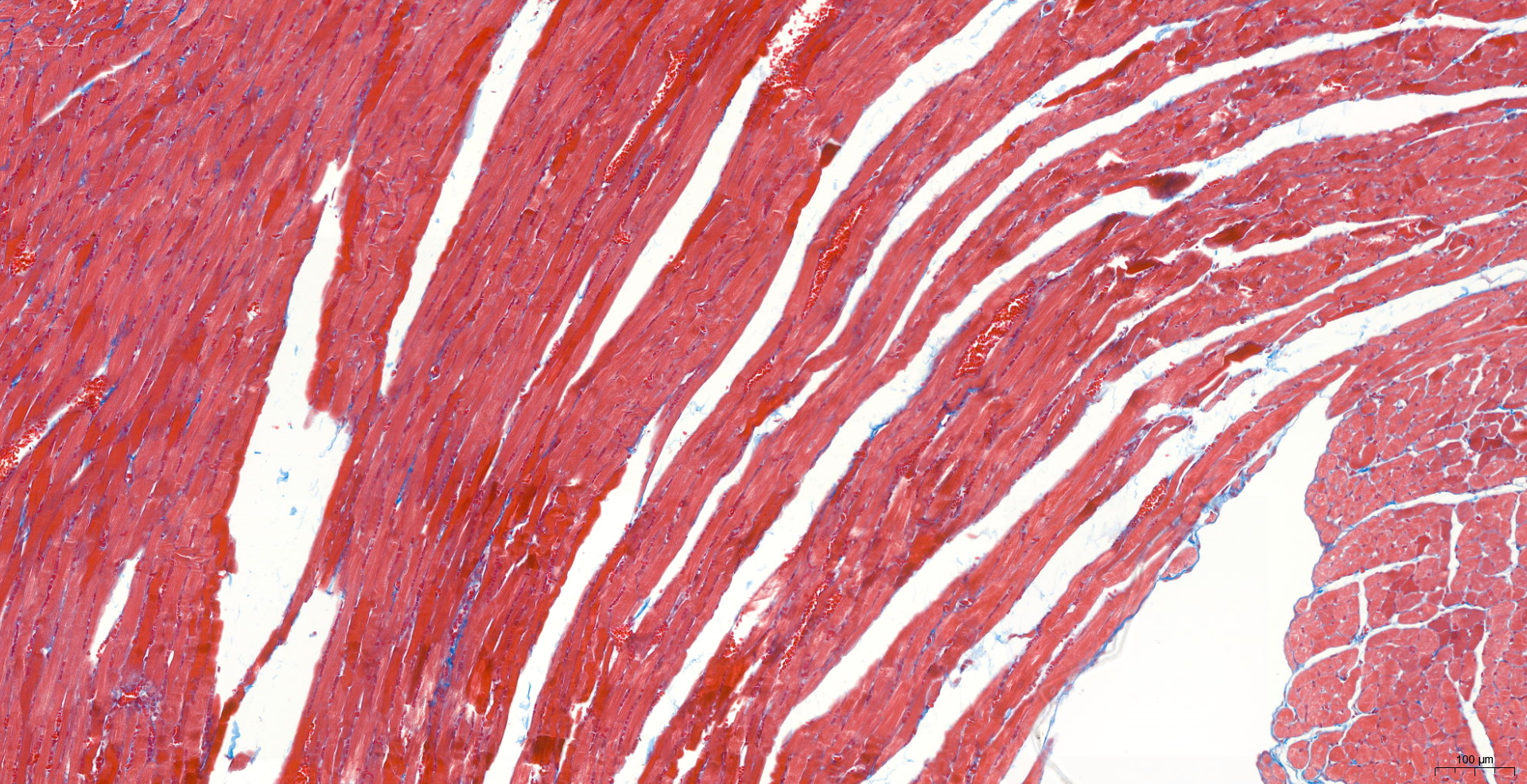

Supplement: Supplementary file 6 [file Data_Sheet_2.ZIP › Figure2 A remote.jpg]

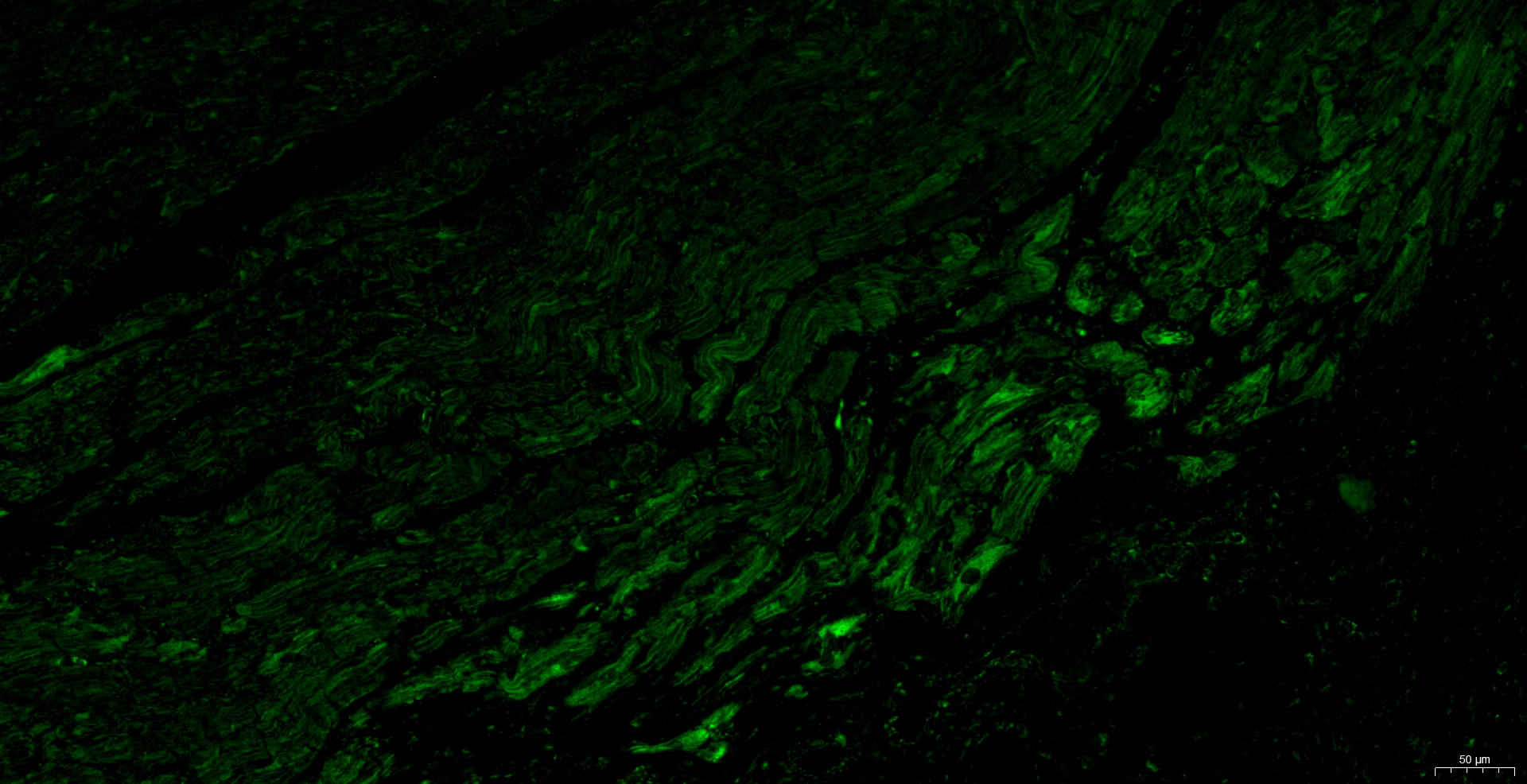

Supplement: Supplementary file 6 [file Data_Sheet_2.ZIP › Figure2 E Border ADAMTS8 20.0x.jpg]

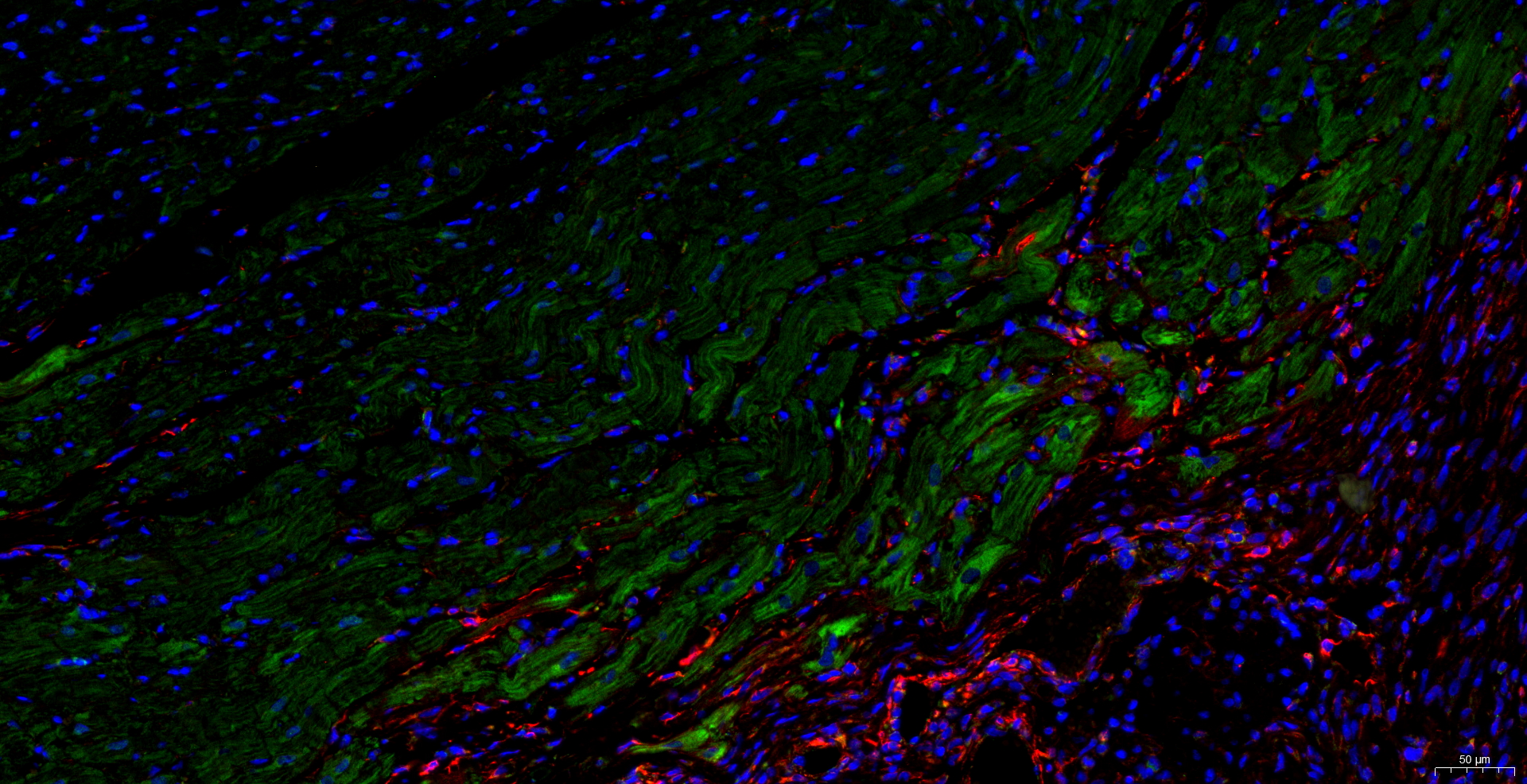

Supplement: Supplementary file 6 [file Data_Sheet_2.ZIP › Figure2 E Border merge 20.0x.jpg]

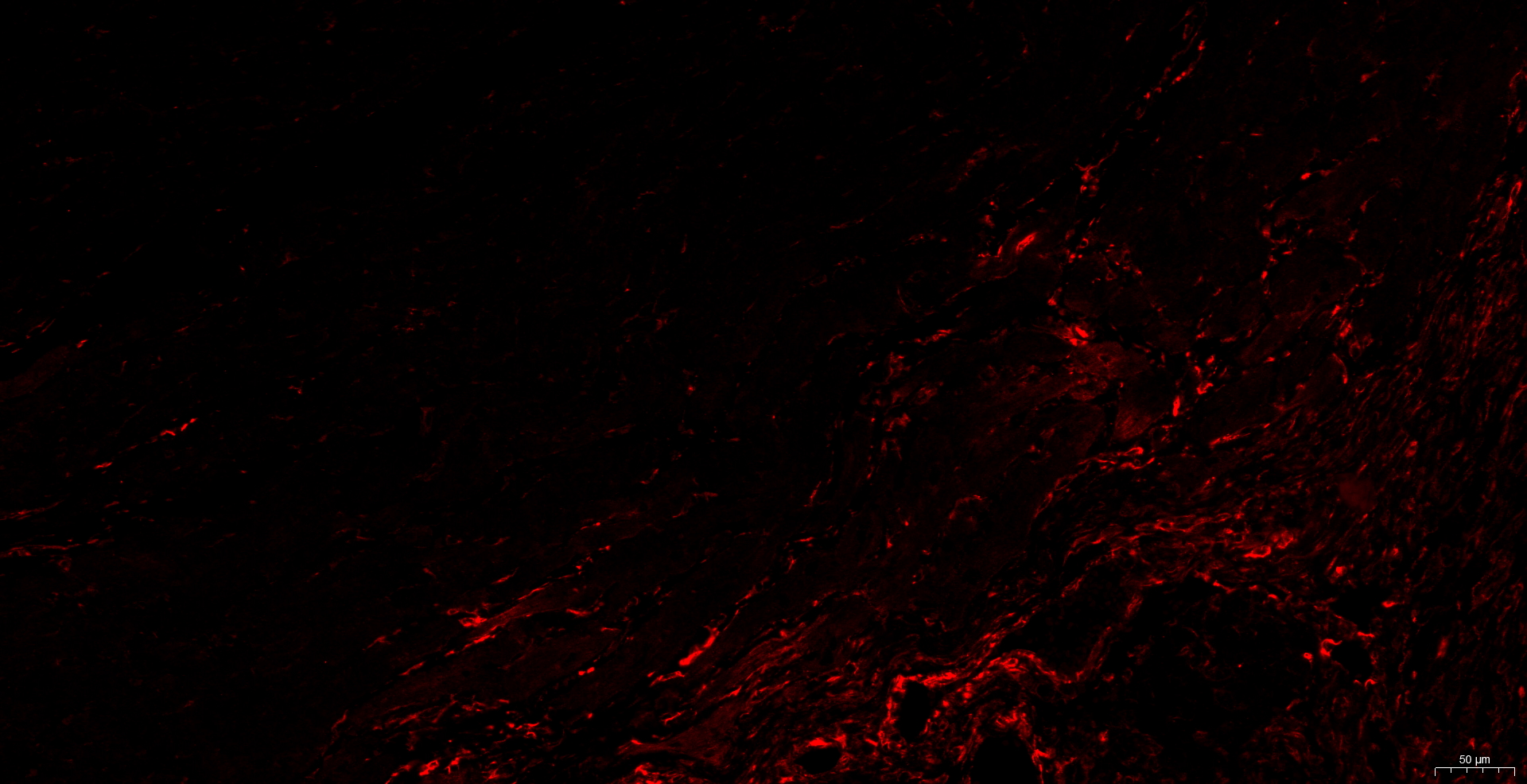

Supplement: Supplementary file 6 [file Data_Sheet_2.ZIP › Figure2 E border Vimentin 20.0x.jpg]

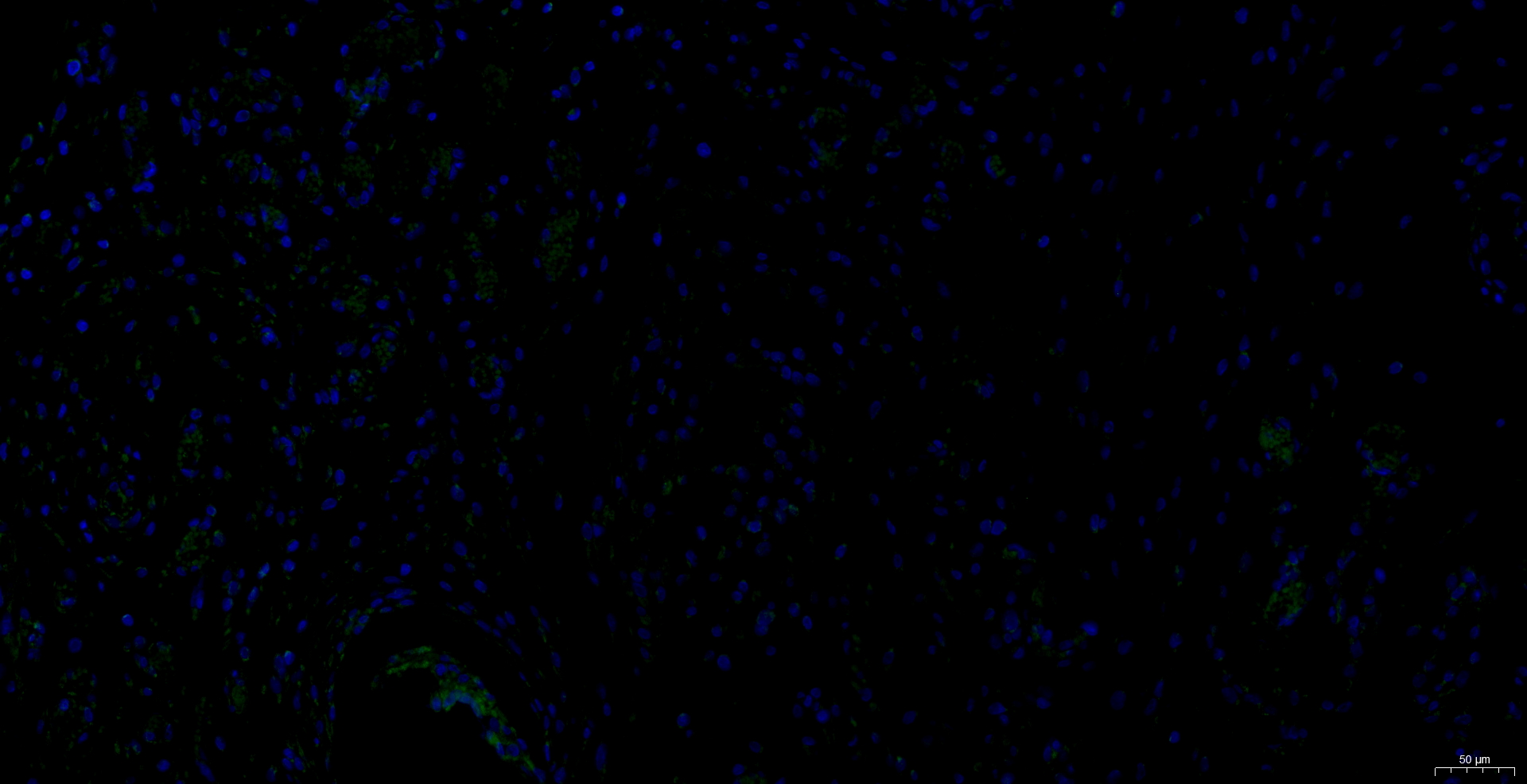

Supplement: Supplementary file 6 [file Data_Sheet_2.ZIP › Figure2 E Infarct ADAMTS8 20.0x.jpg]

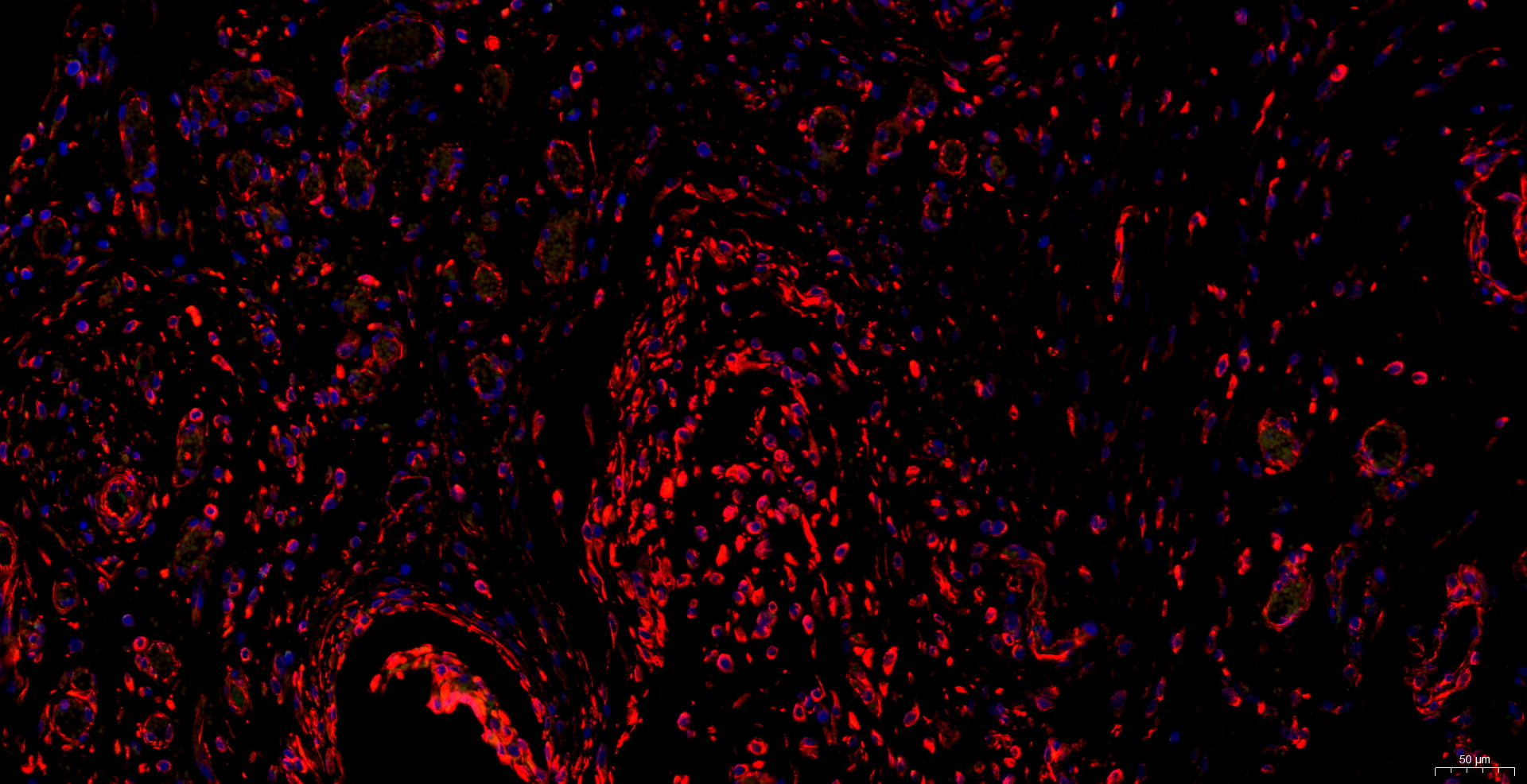

Supplement: Supplementary file 6 [file Data_Sheet_2.ZIP › Figure2 E infarct merge 20.0x.jpg]

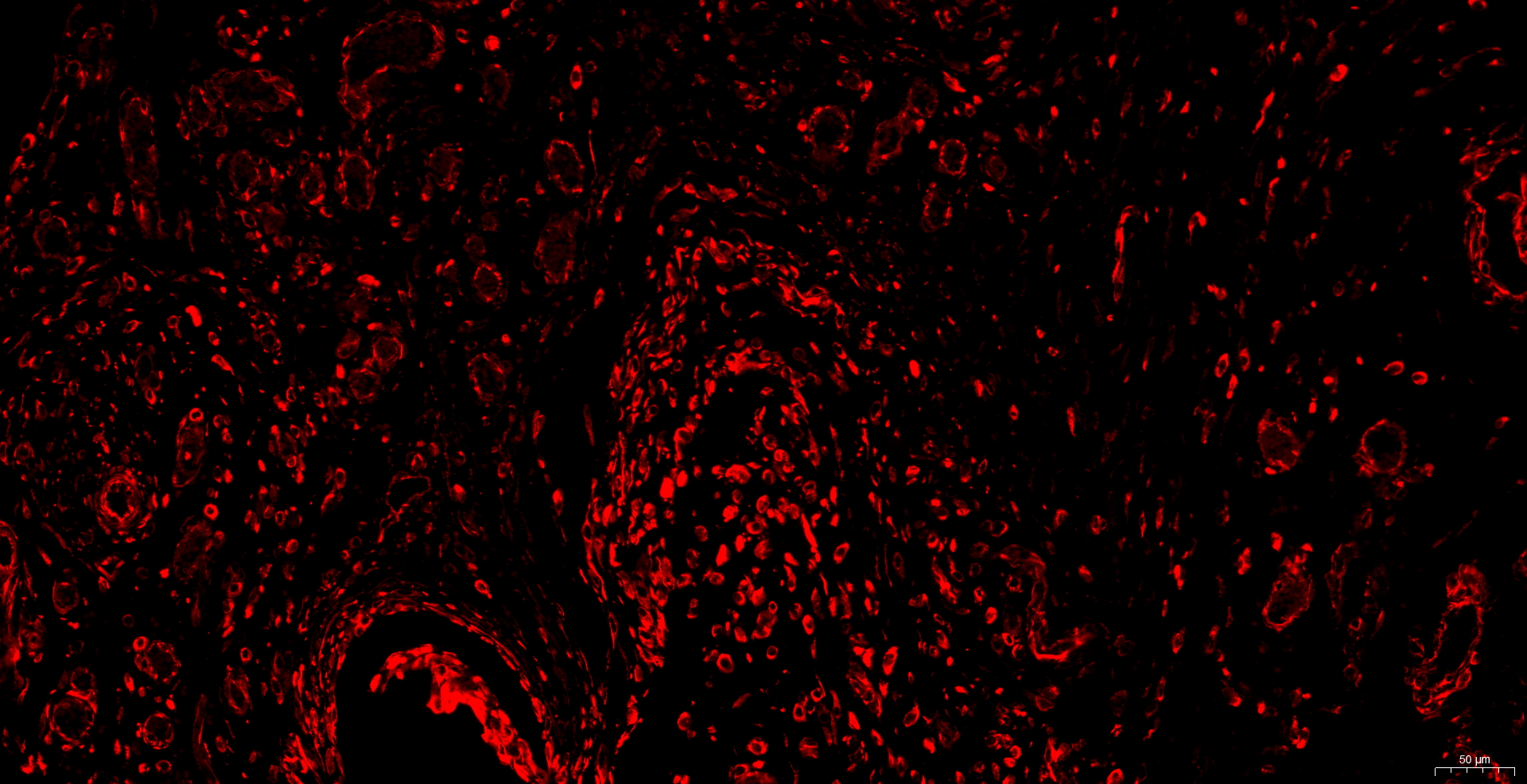

Supplement: Supplementary file 6 [file Data_Sheet_2.ZIP › Figure2 E infarct vimentin 20.0x.jpg]

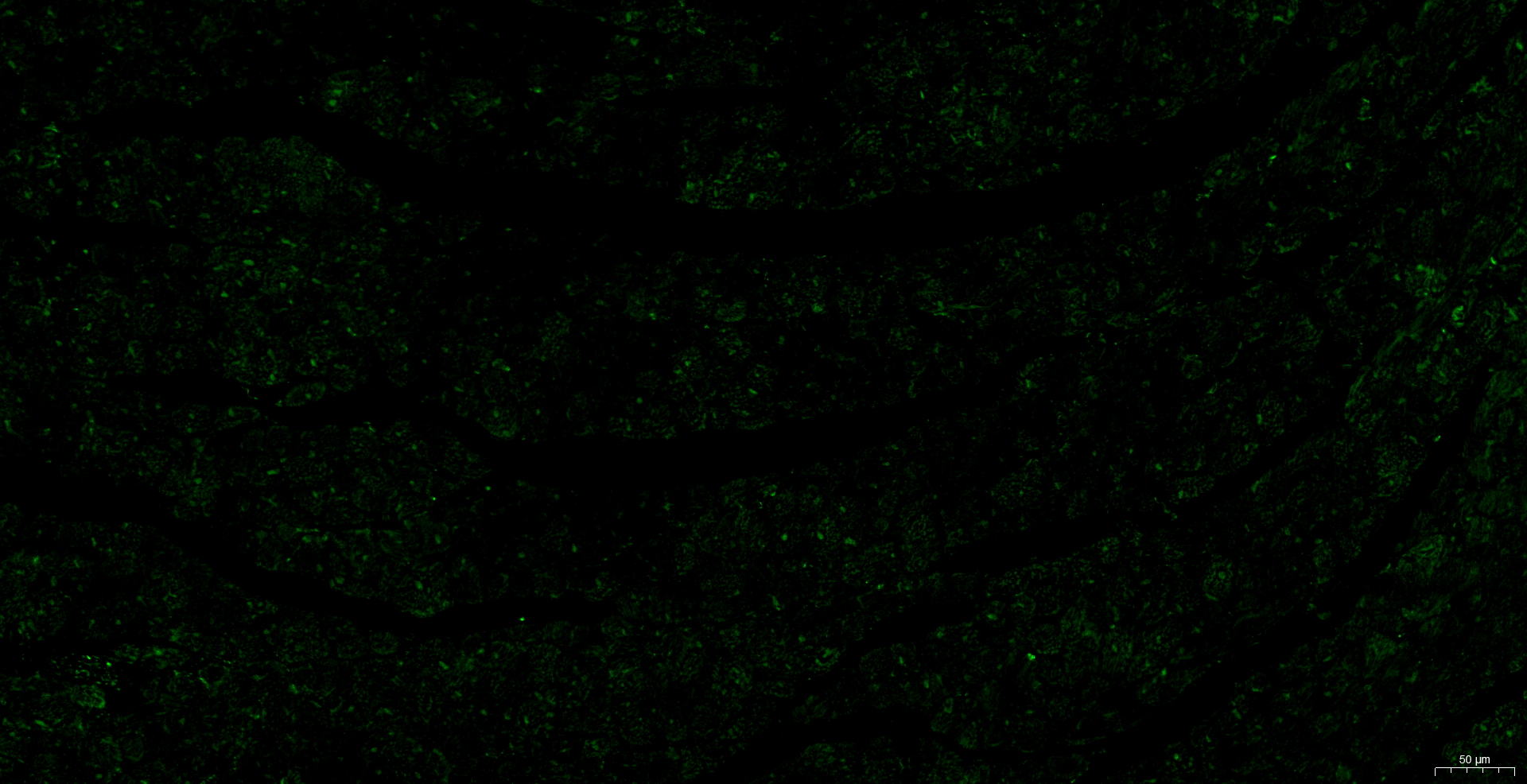

Supplement: Supplementary file 6 [file Data_Sheet_2.ZIP › Figure2 E Remote ADAMTS8 20.0x.jpg]

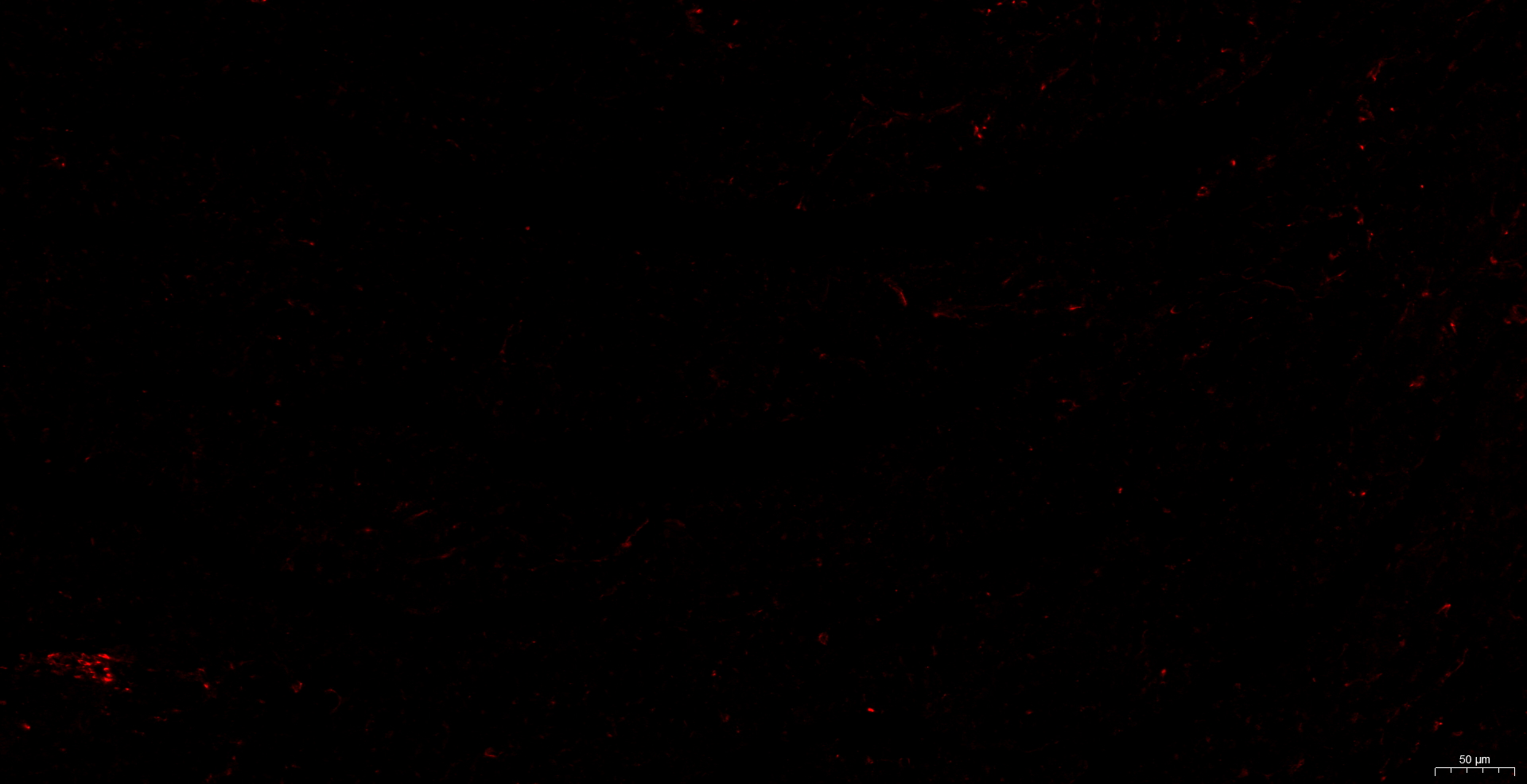

Supplement: Supplementary file 6 [file Data_Sheet_2.ZIP › Figure2 E Remote Vimentin 20.0x.jpg]

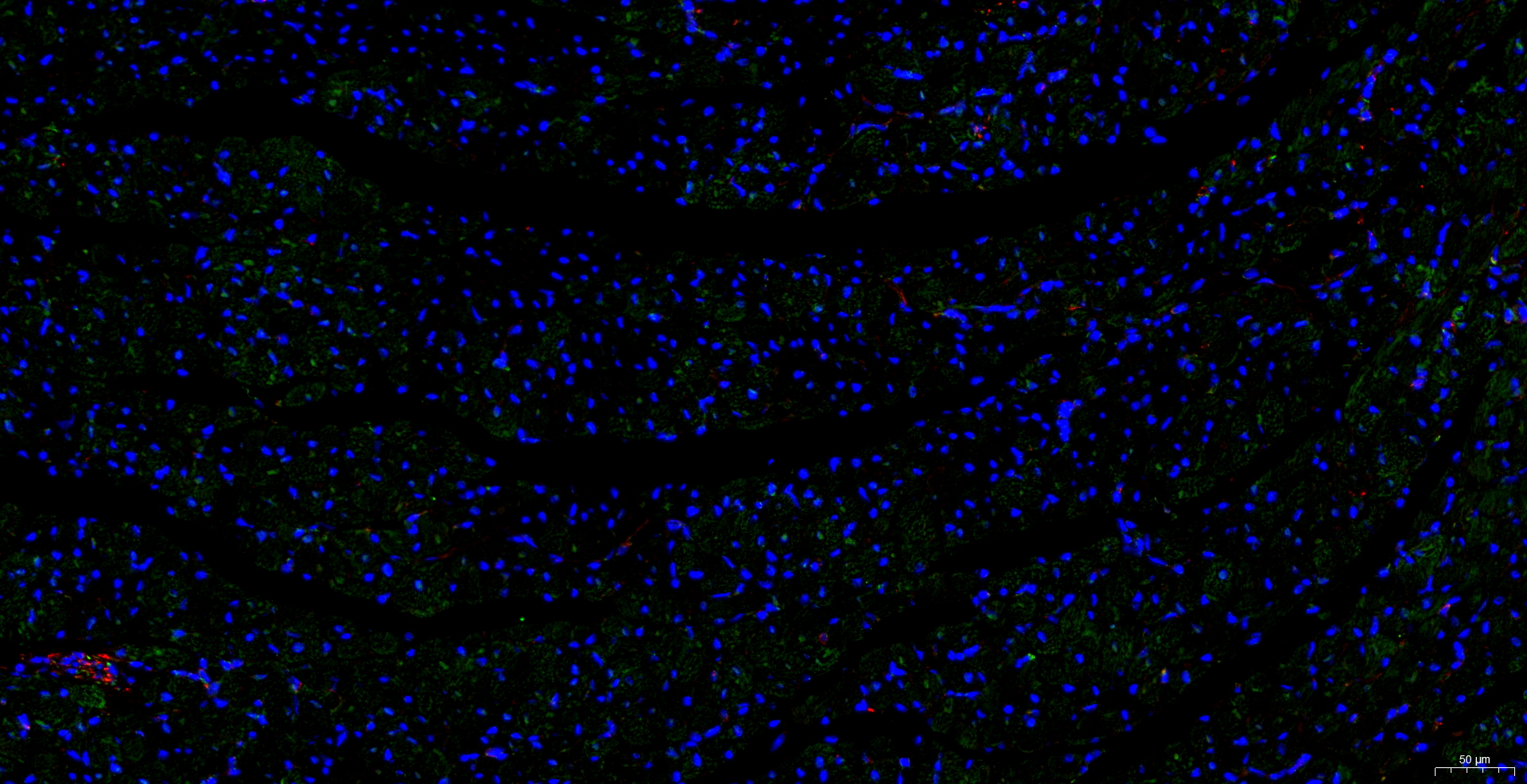

Supplement: Supplementary file 6 [file Data_Sheet_2.ZIP › Figure2 E Remote MERGE 20.0x.jpg]

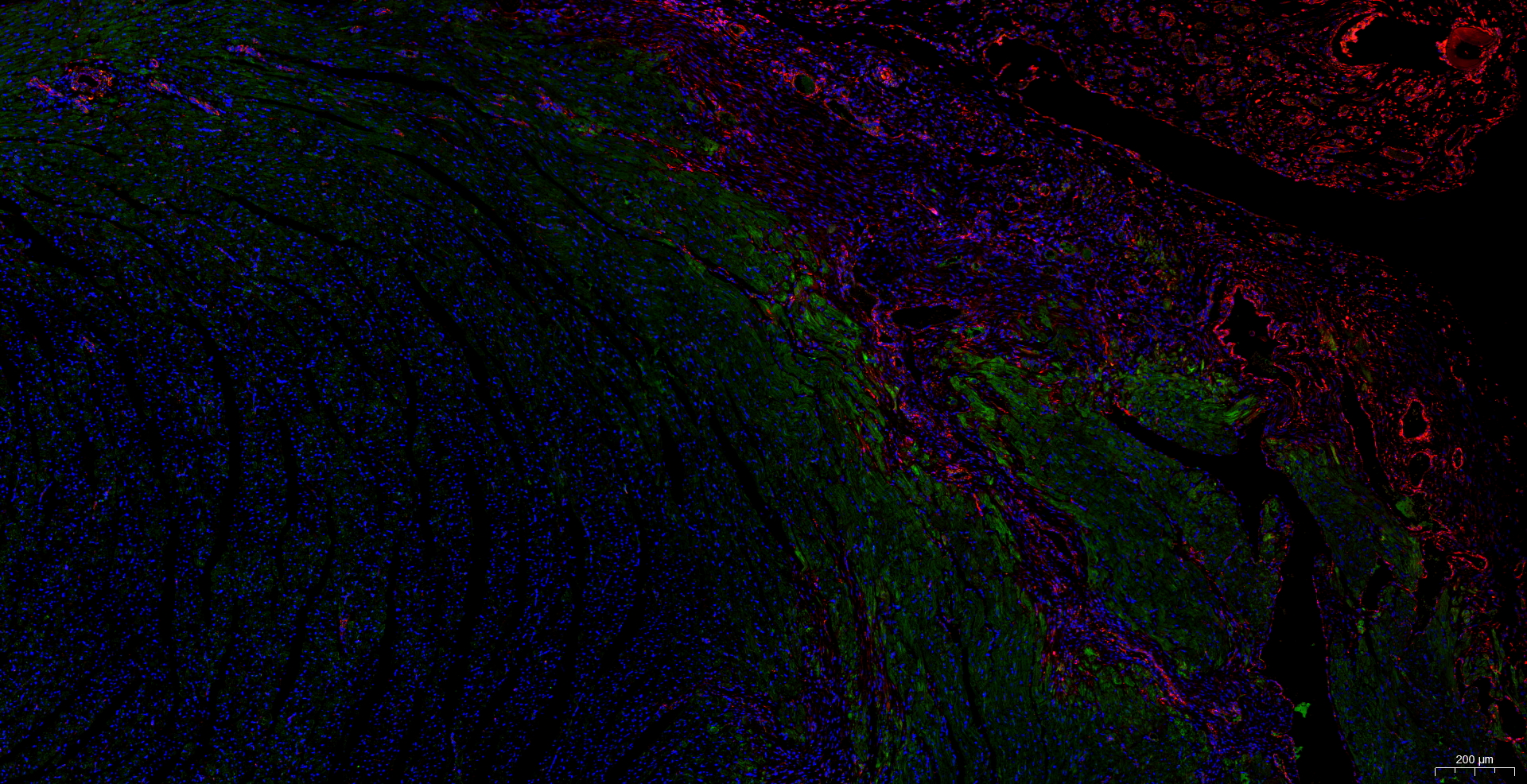

Supplement: Supplementary file 6 [file Data_Sheet_2.ZIP › Figure2 E.jpg]

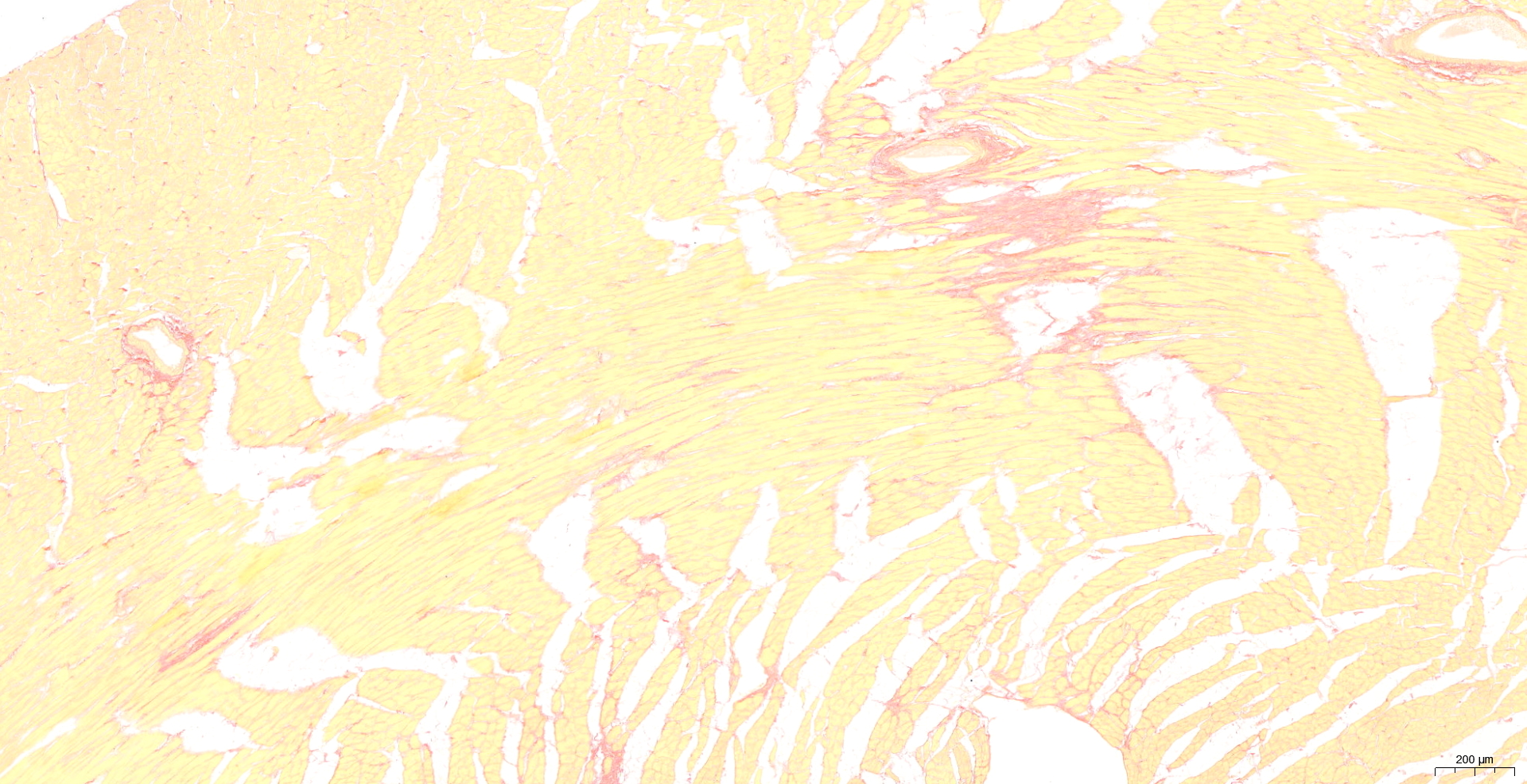

Supplement: Supplementary file 7 [file Data_Sheet_3.ZIP › Figure3 A TAC.jpg]

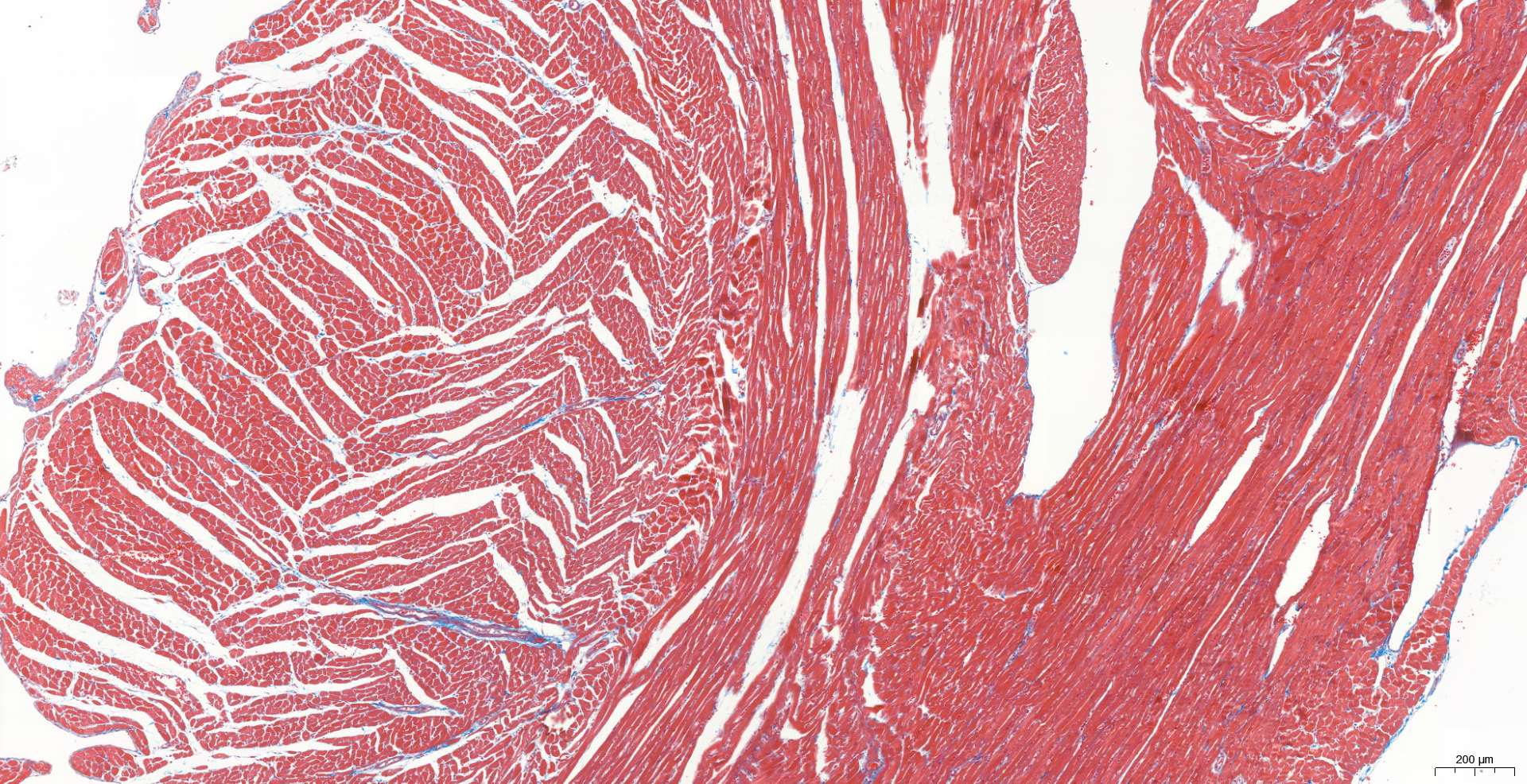

Supplement: Supplementary file 7 [file Data_Sheet_3.ZIP › Figure3 A Sham Masson.jpg]

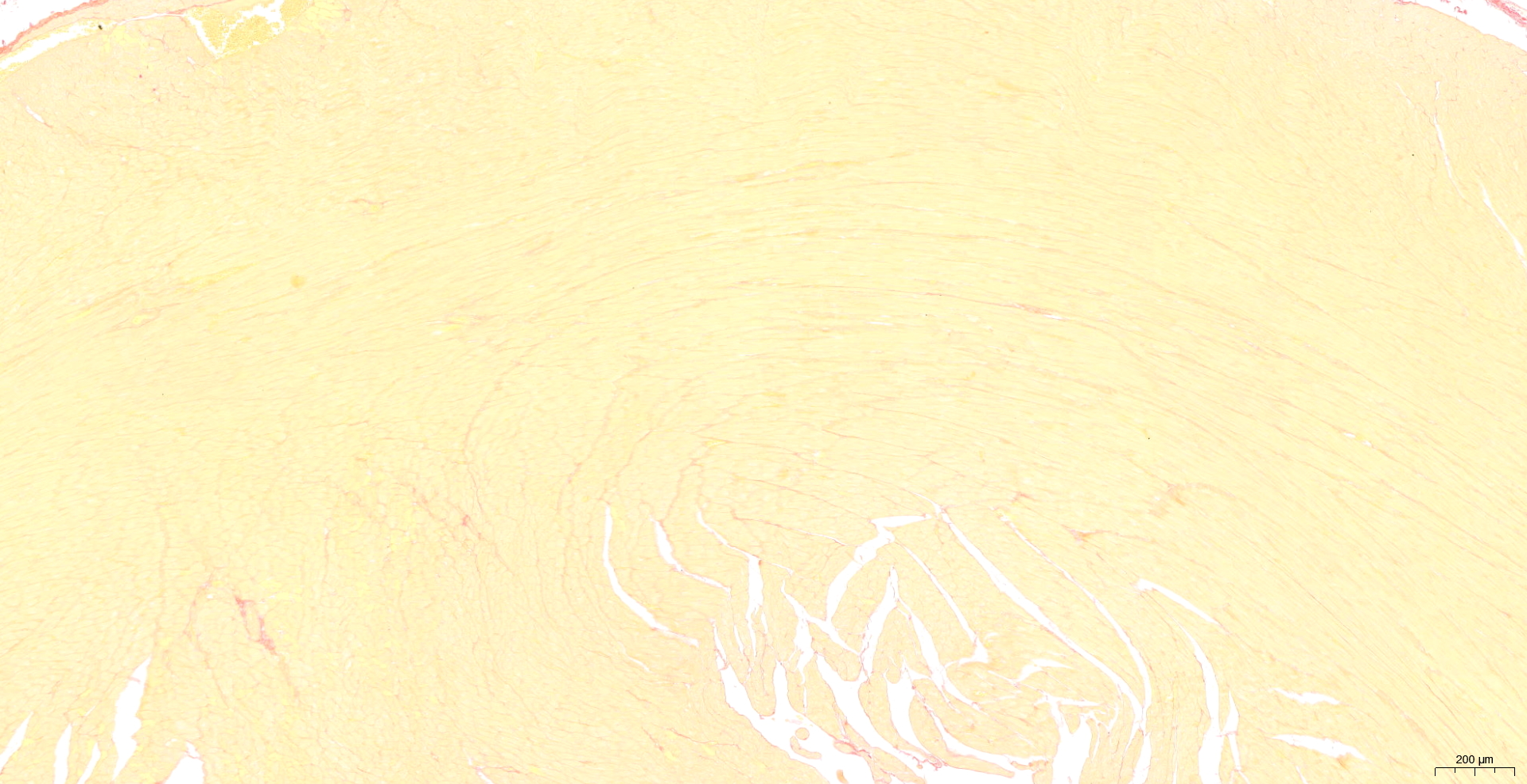

Supplement: Supplementary file 7 [file Data_Sheet_3.ZIP › Figure3 A sham.jpg]

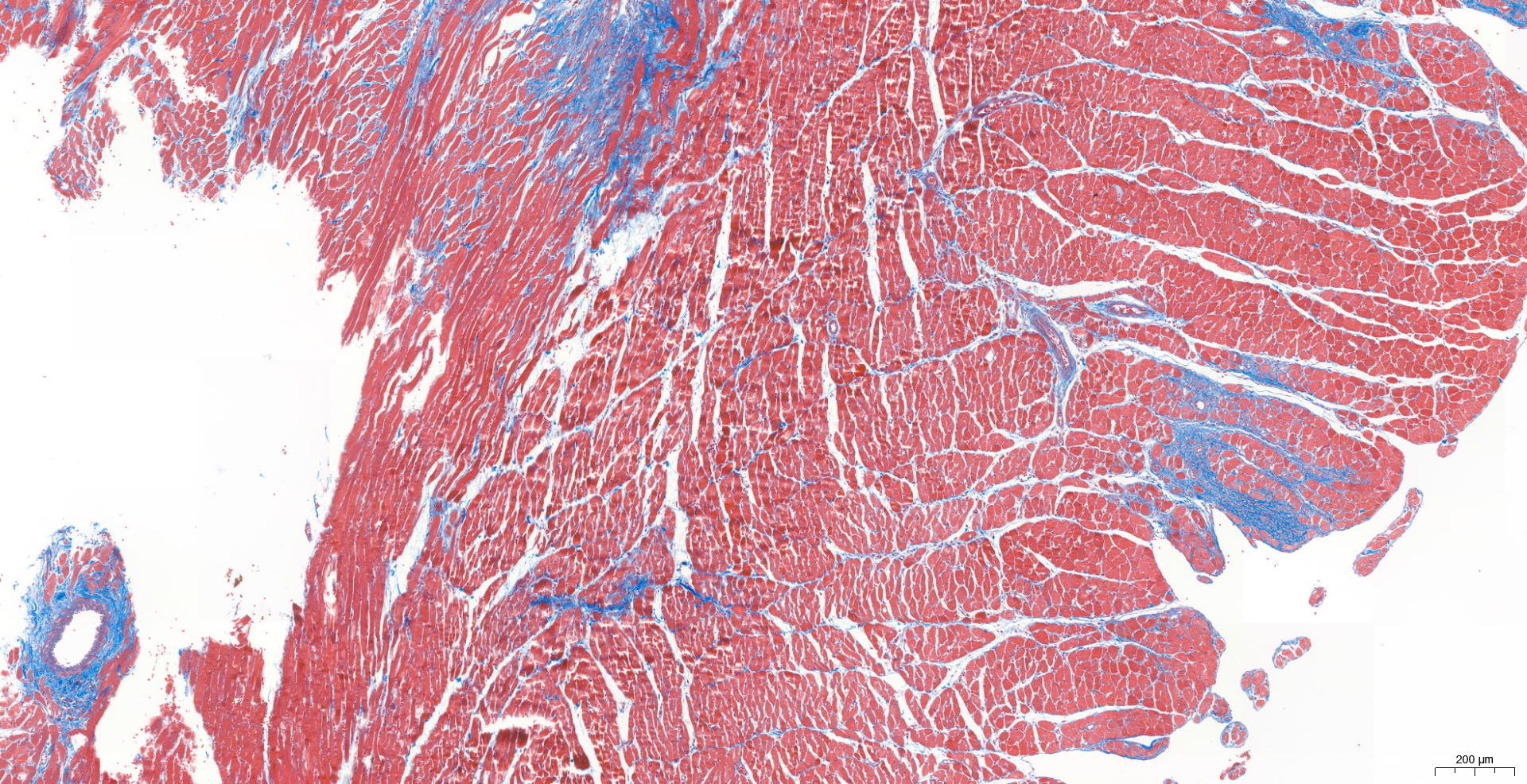

Supplement: Supplementary file 7 [file Data_Sheet_3.ZIP › Figure3 A TAC Masson.jpg]

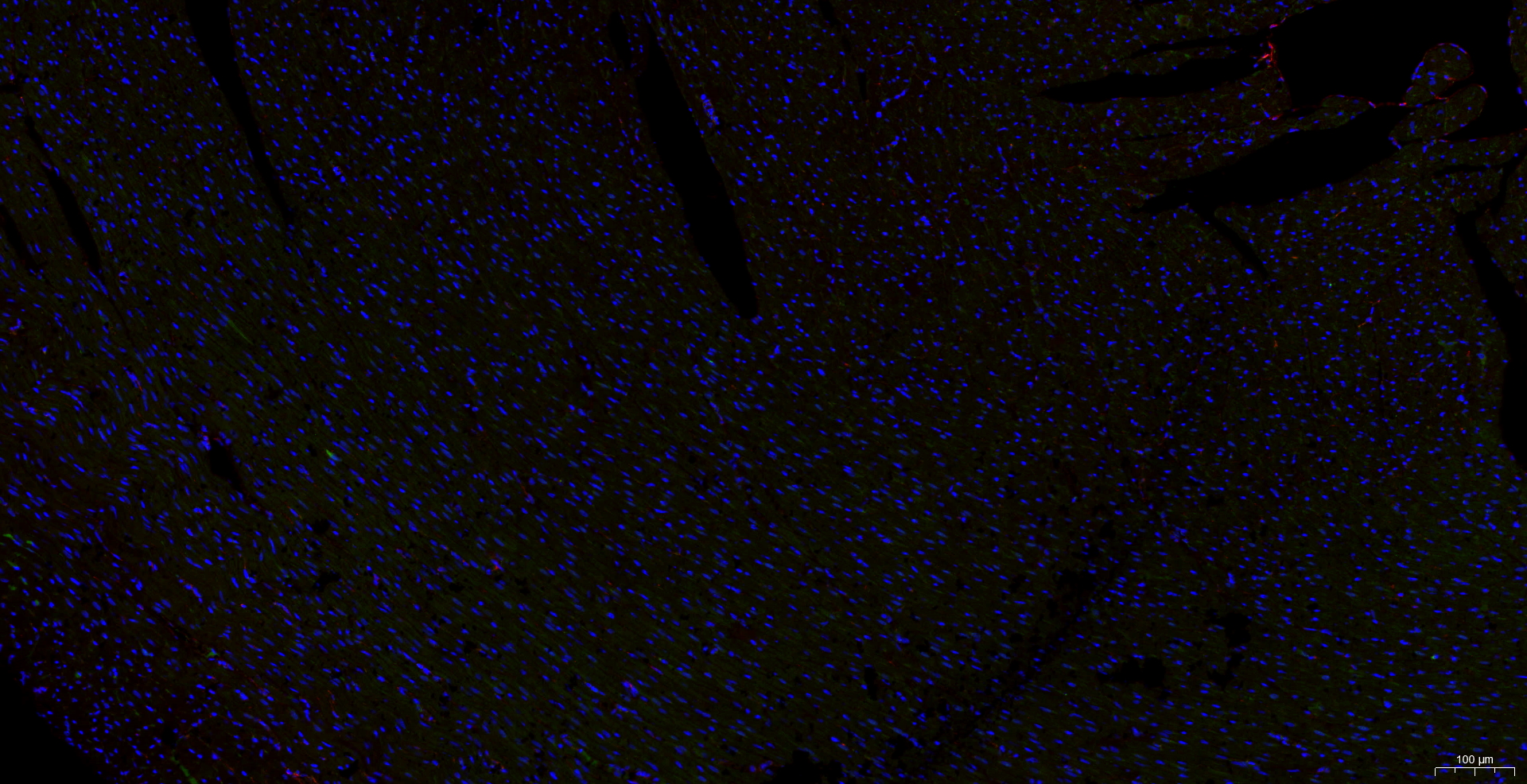

Supplement: Supplementary file 7 [file Data_Sheet_3.ZIP › Figure3 C sham merge.jpg]

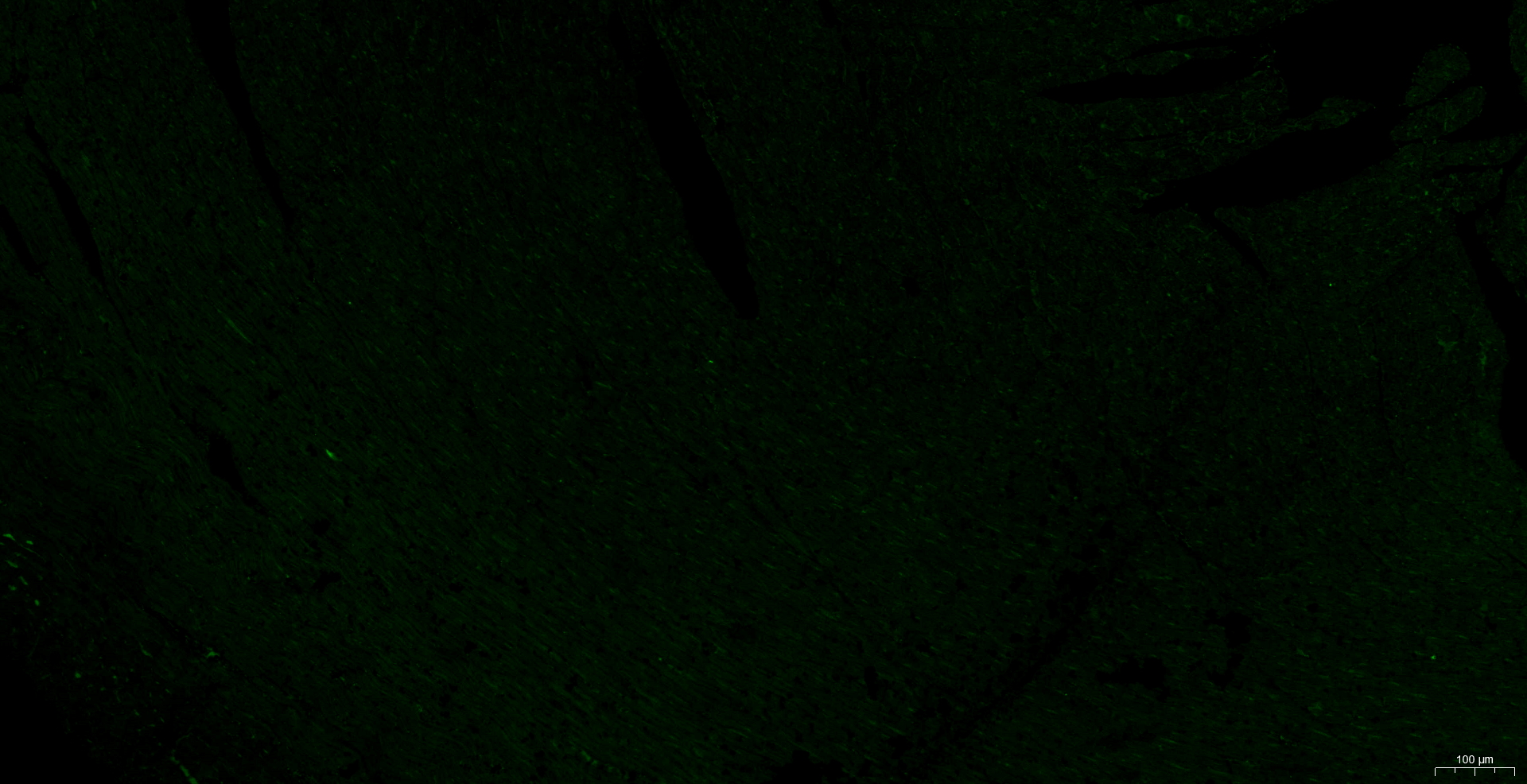

Supplement: Supplementary file 7 [file Data_Sheet_3.ZIP › Figure3 C sham ADAMTS8.jpg]

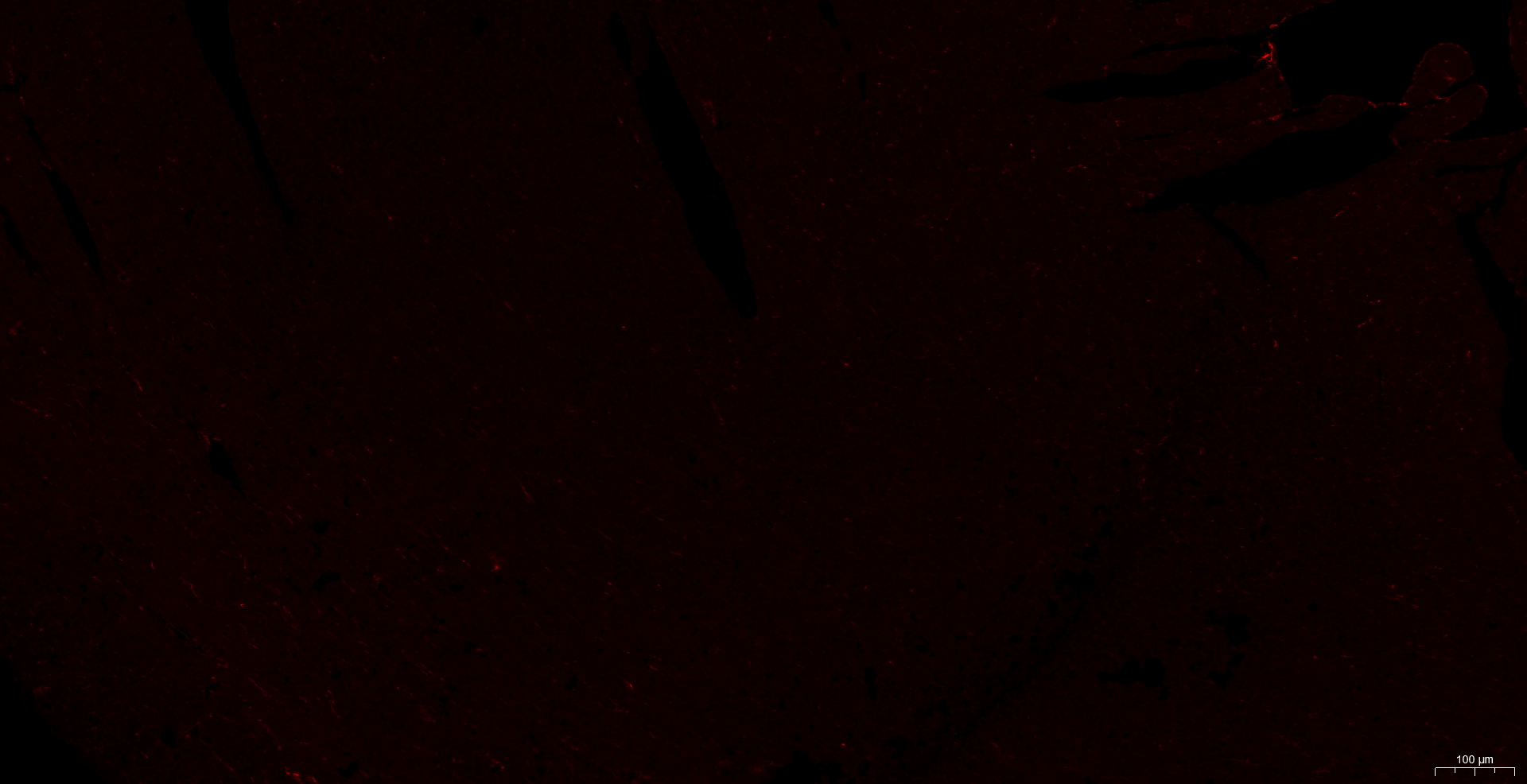

Supplement: Supplementary file 7 [file Data_Sheet_3.ZIP › Figure3 C sham Vimentin.jpg]

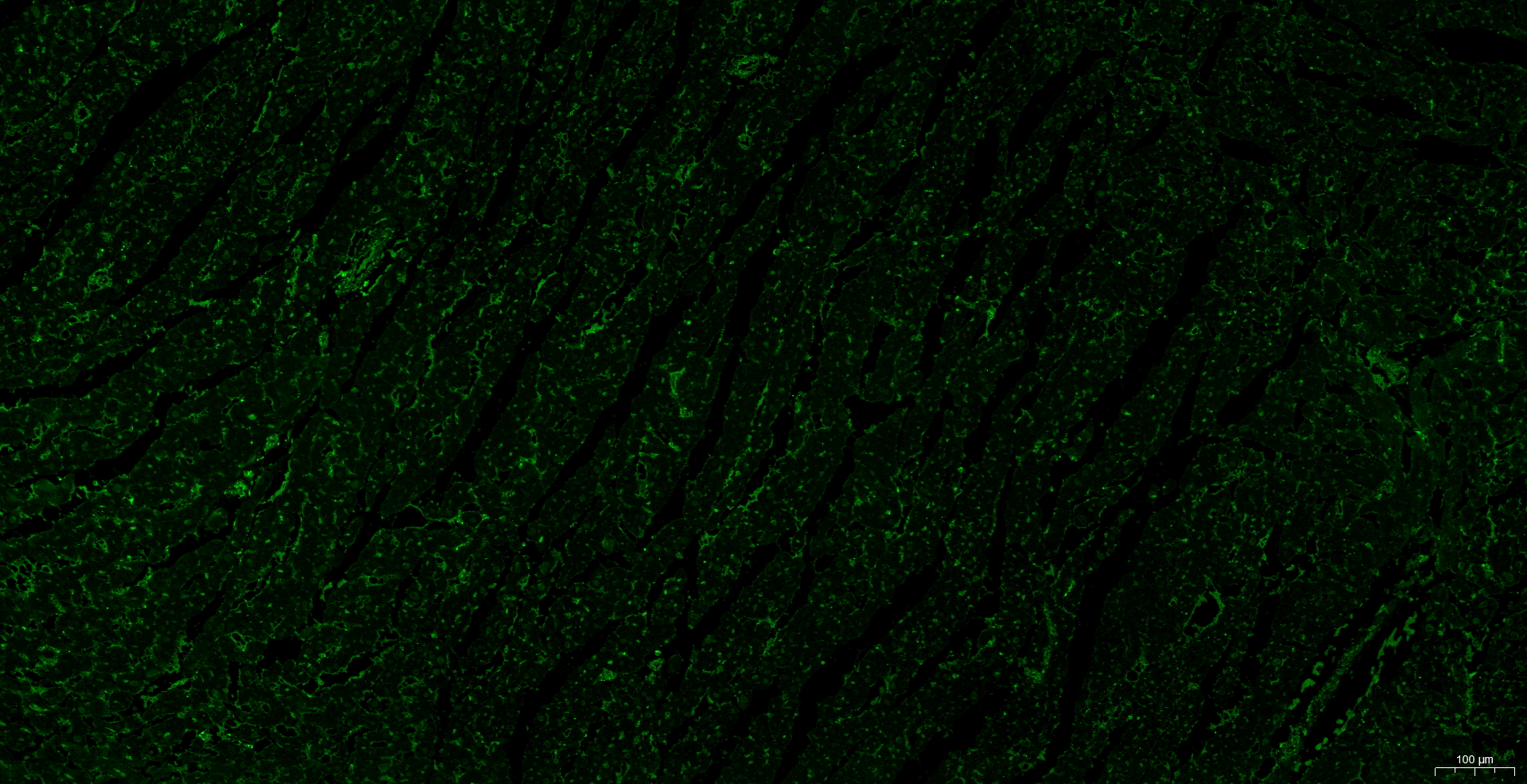

Supplement: Supplementary file 7 [file Data_Sheet_3.ZIP › Figure3 C TAC ADAMTS8.jpg]

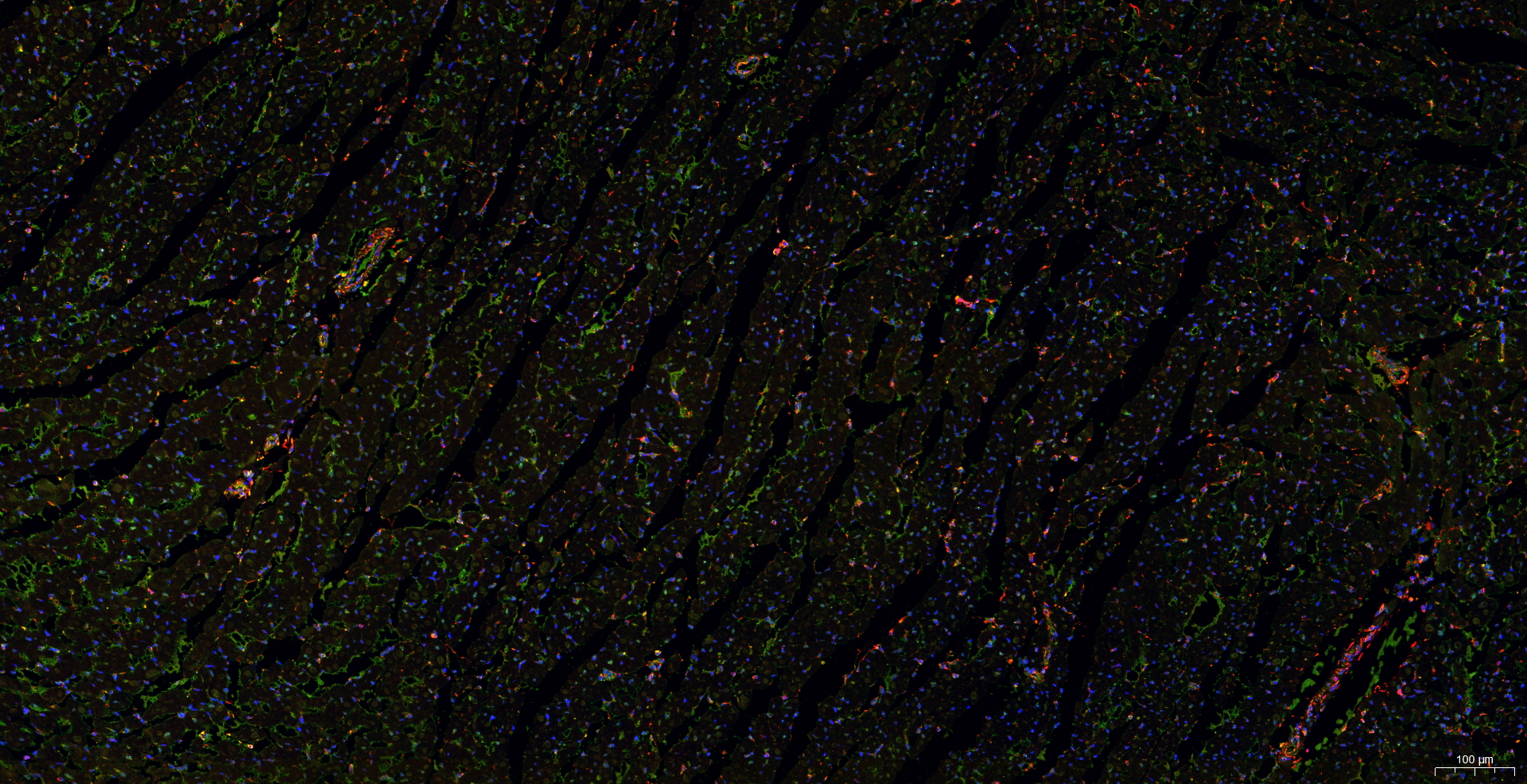

Supplement: Supplementary file 7 [file Data_Sheet_3.ZIP › Figure3 C TAC MERGE.jpg]

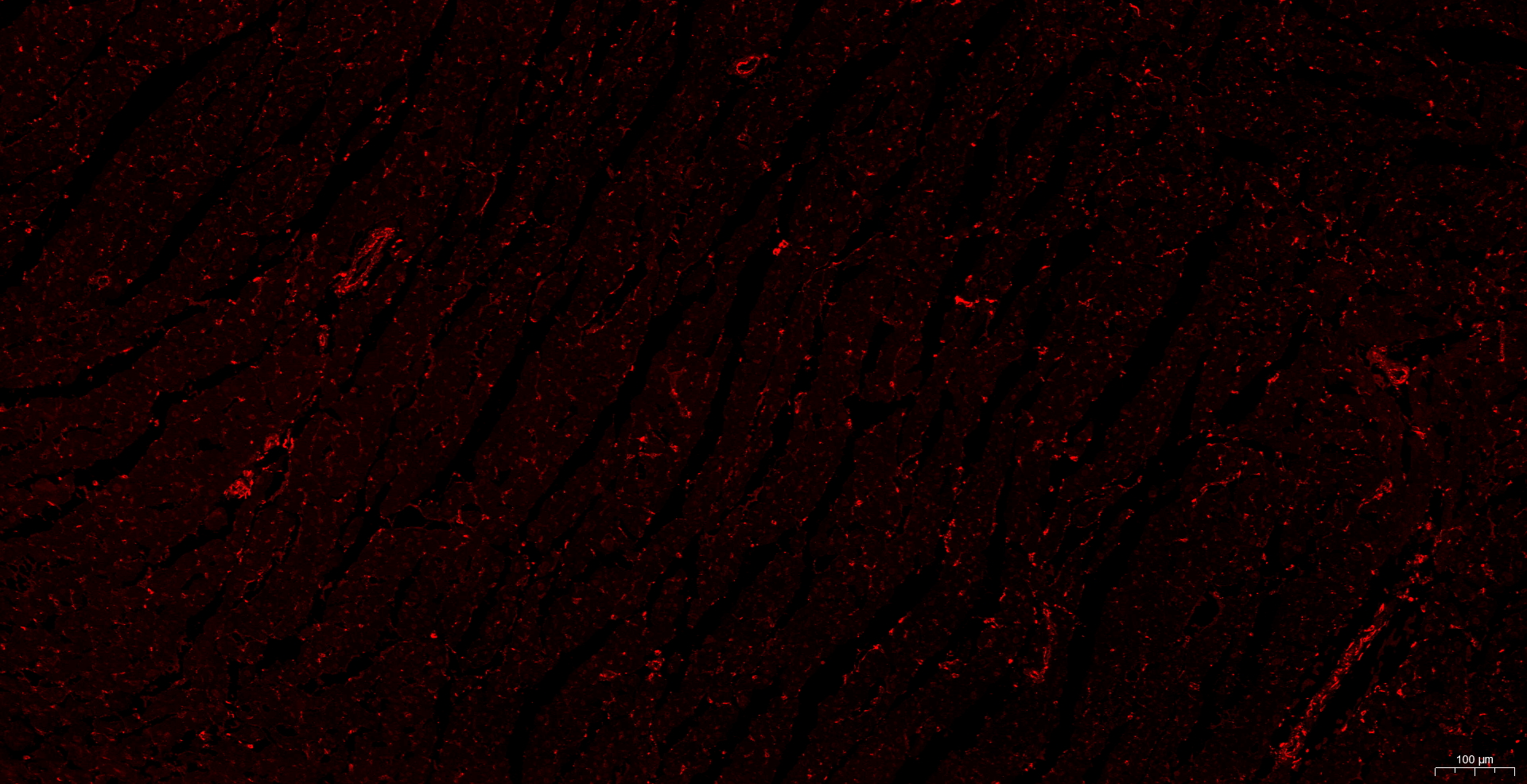

Supplement: Supplementary file 7 [file Data_Sheet_3.ZIP › Figure3 C TAC Vimentin.jpg]

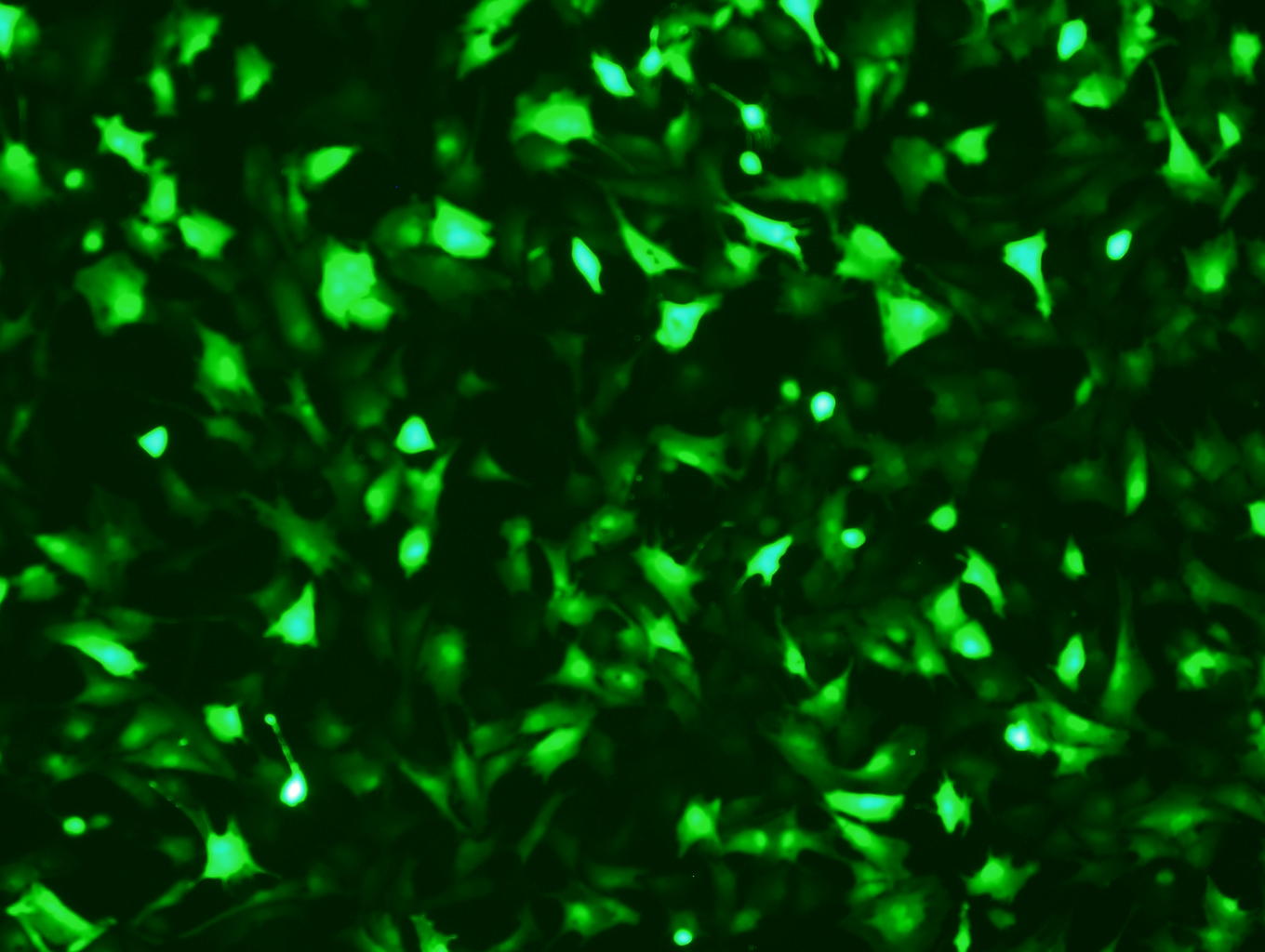

Supplement: Supplementary file 8 [file Data_Sheet_4.ZIP › Figure4 B Ad-ADAMTS8.tif]

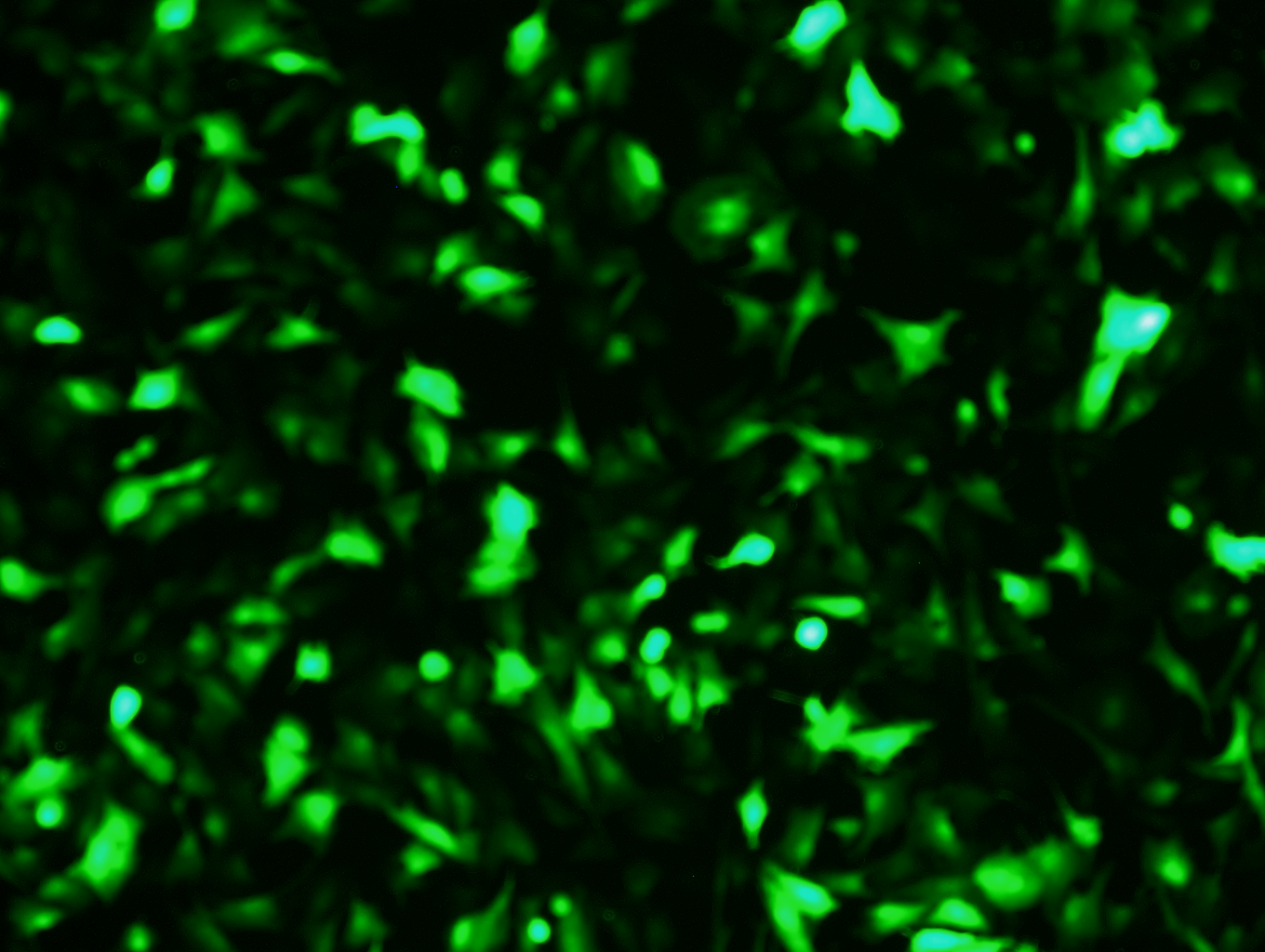

Supplement: Supplementary file 8 [file Data_Sheet_4.ZIP › Figure4 B Ad-v.tif]

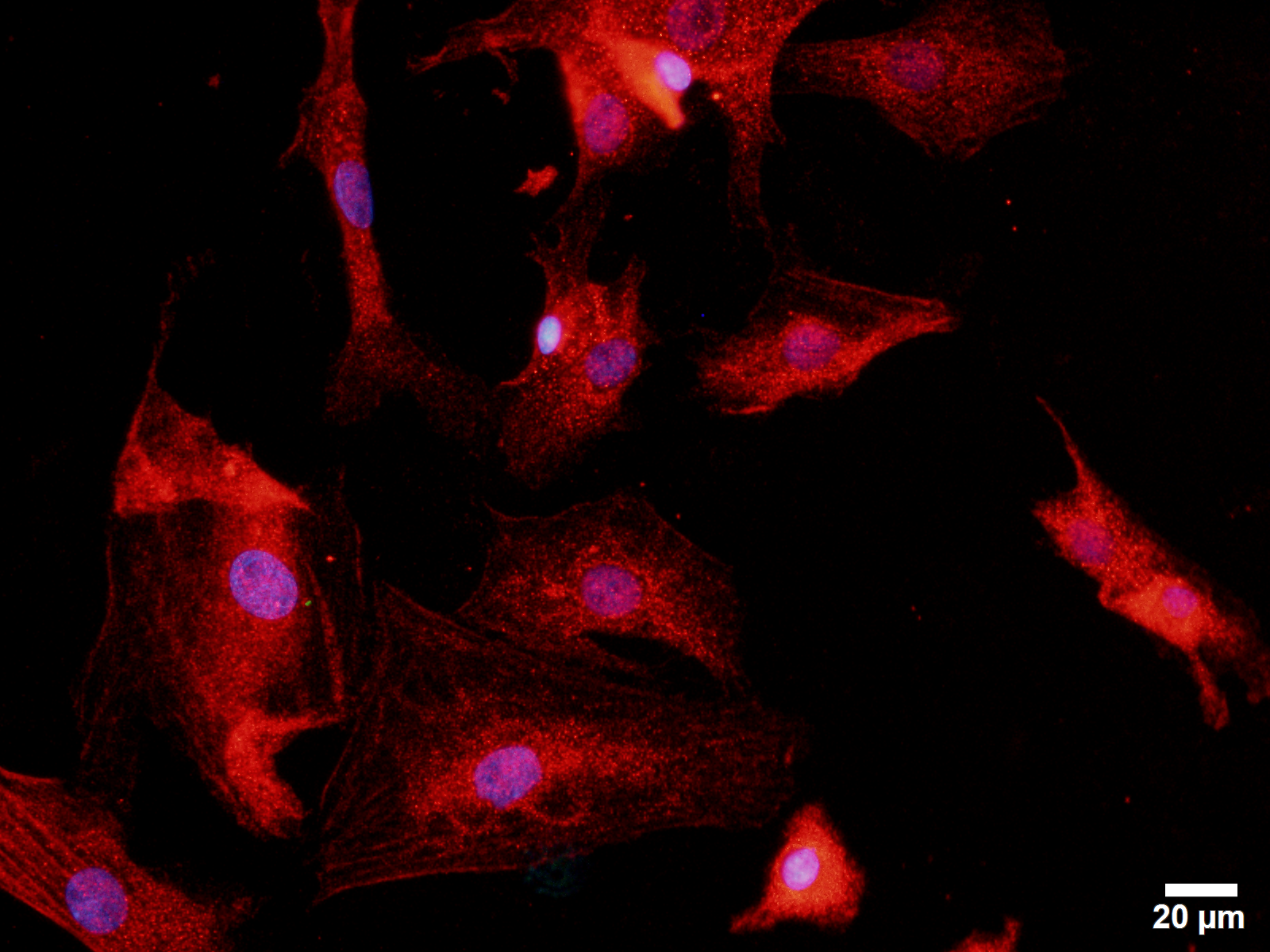

Supplement: Supplementary file 8 [file Data_Sheet_4.ZIP › Figure4 E Ad-ADAMTS8.tif]

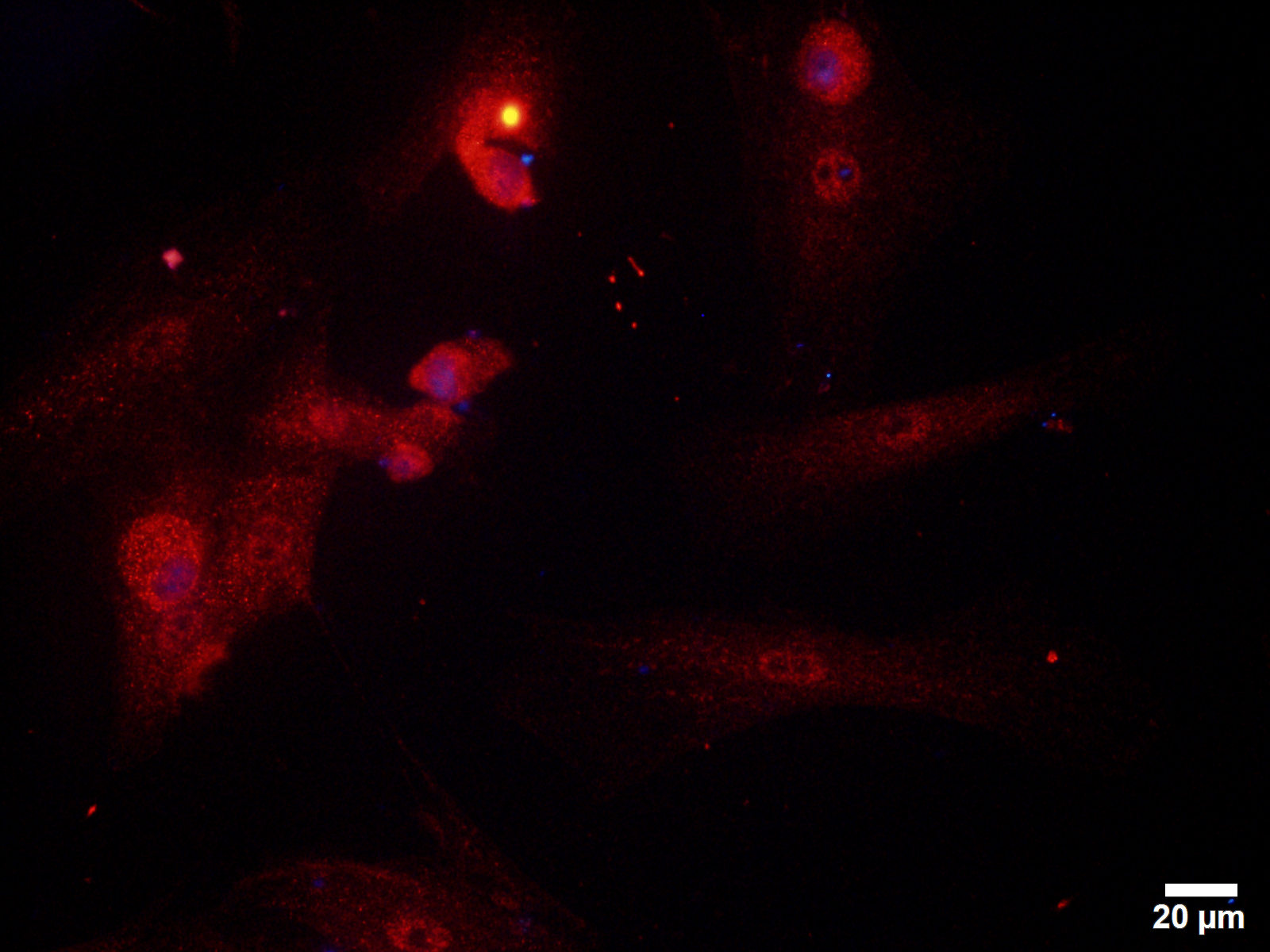

Supplement: Supplementary file 8 [file Data_Sheet_4.ZIP › Figure4 E Ad-v.tif]

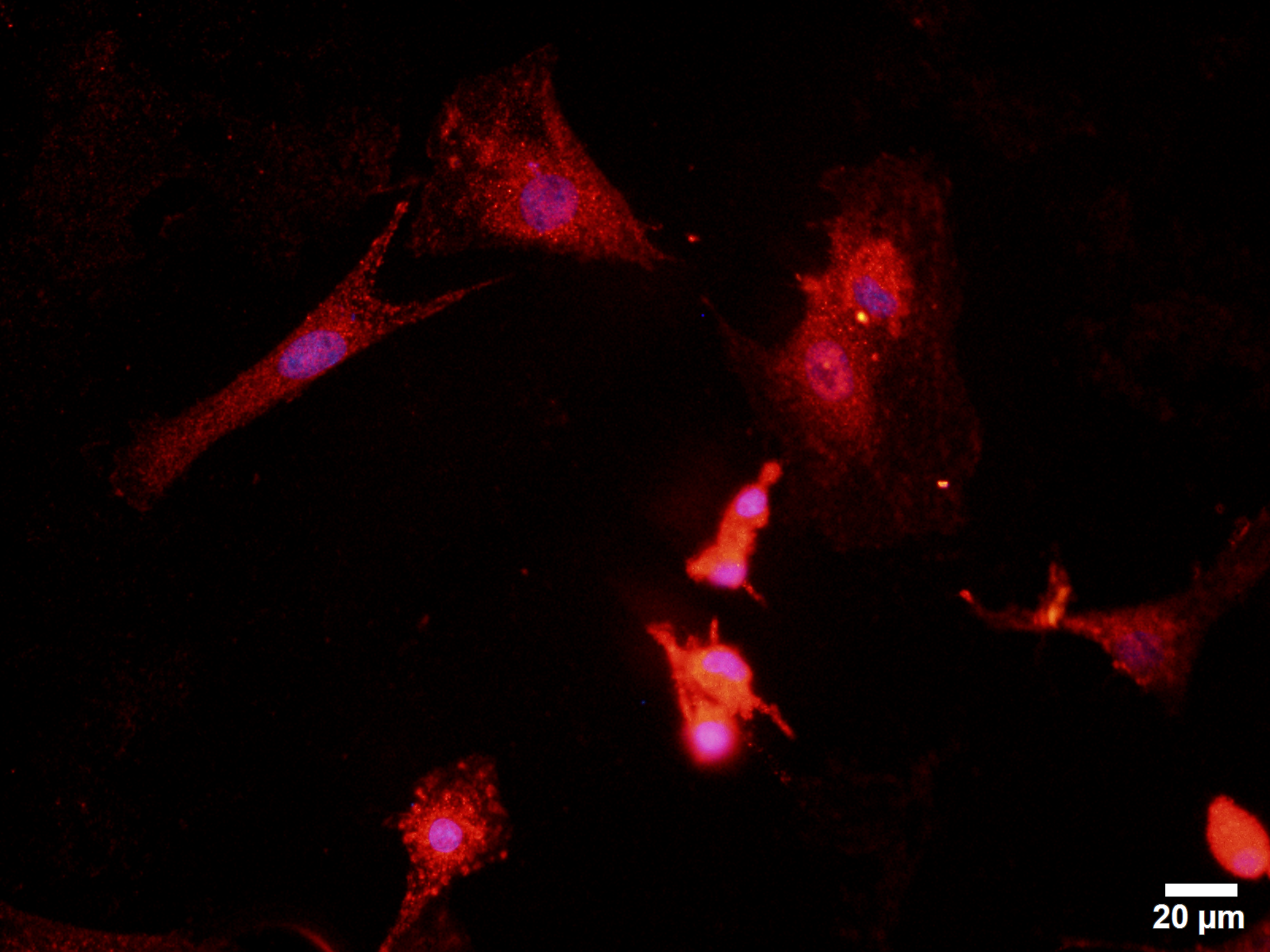

Supplement: Supplementary file 8 [file Data_Sheet_4.ZIP › Figure4 E SCR.tif]

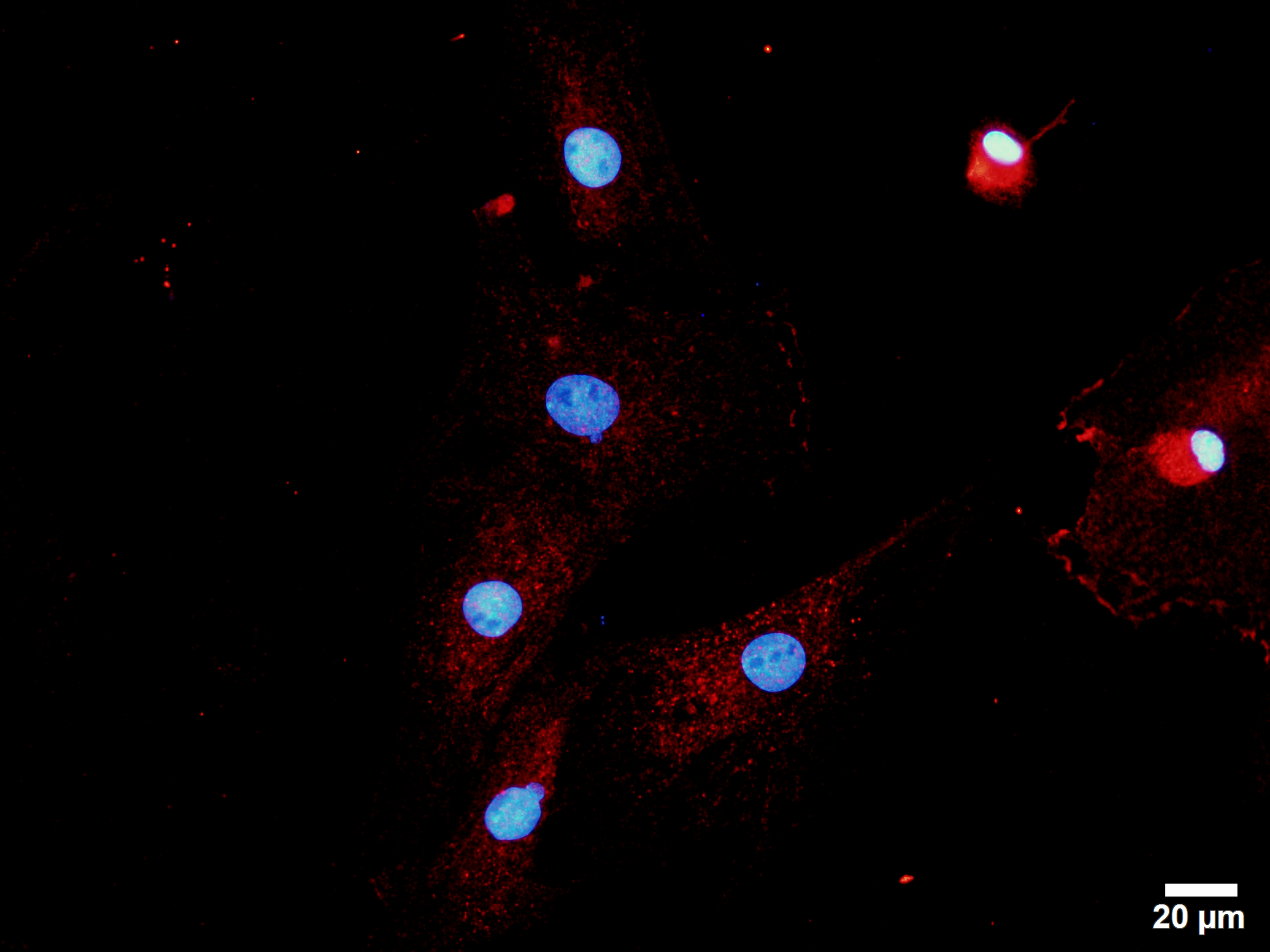

Supplement: Supplementary file 8 [file Data_Sheet_4.ZIP › Figure4 E si ADAMTS8.tif]

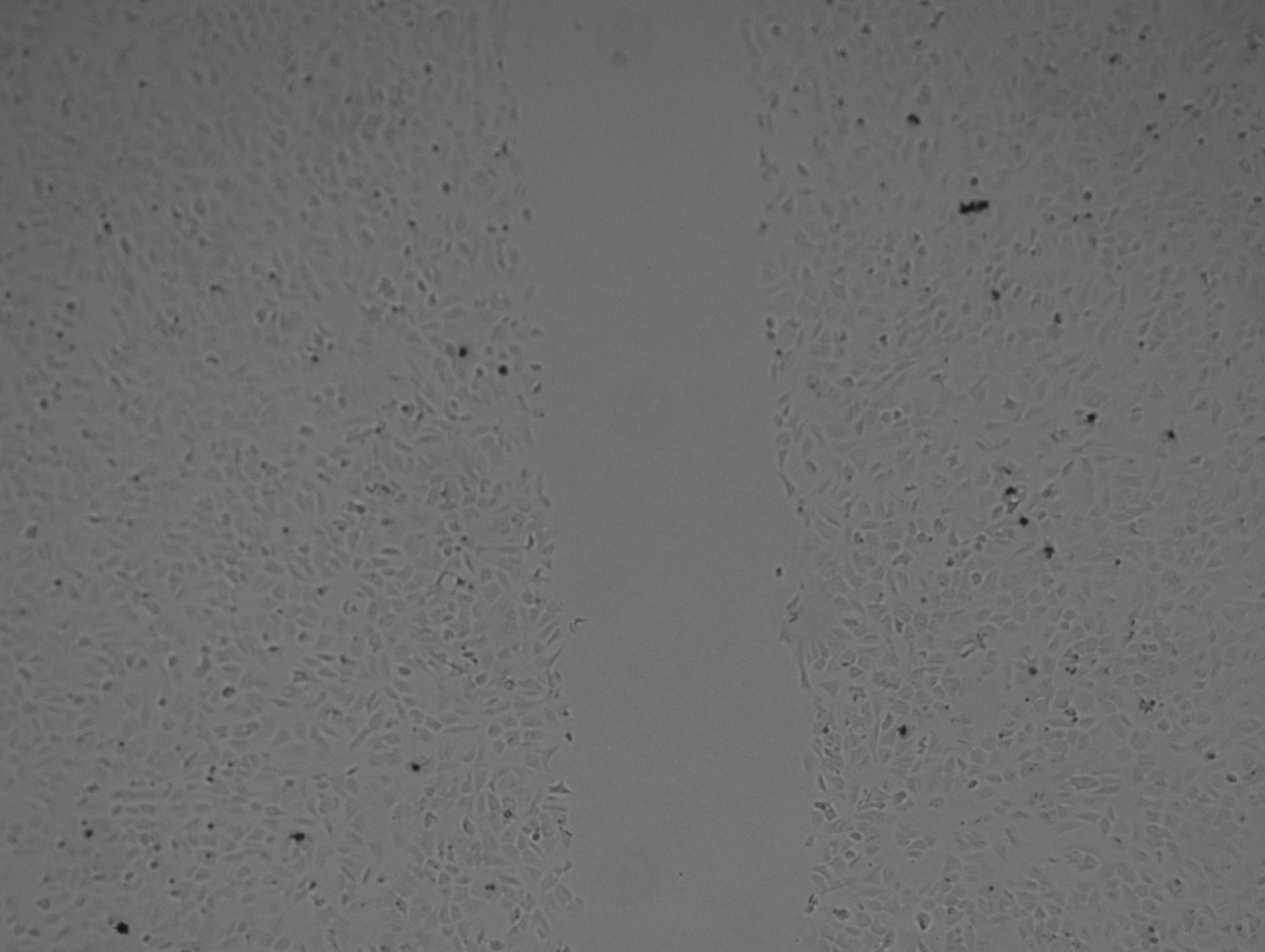

Supplement: Supplementary file 9 [file Data_Sheet_5.ZIP › Figure5 A 0h Co-culture 2.tif]

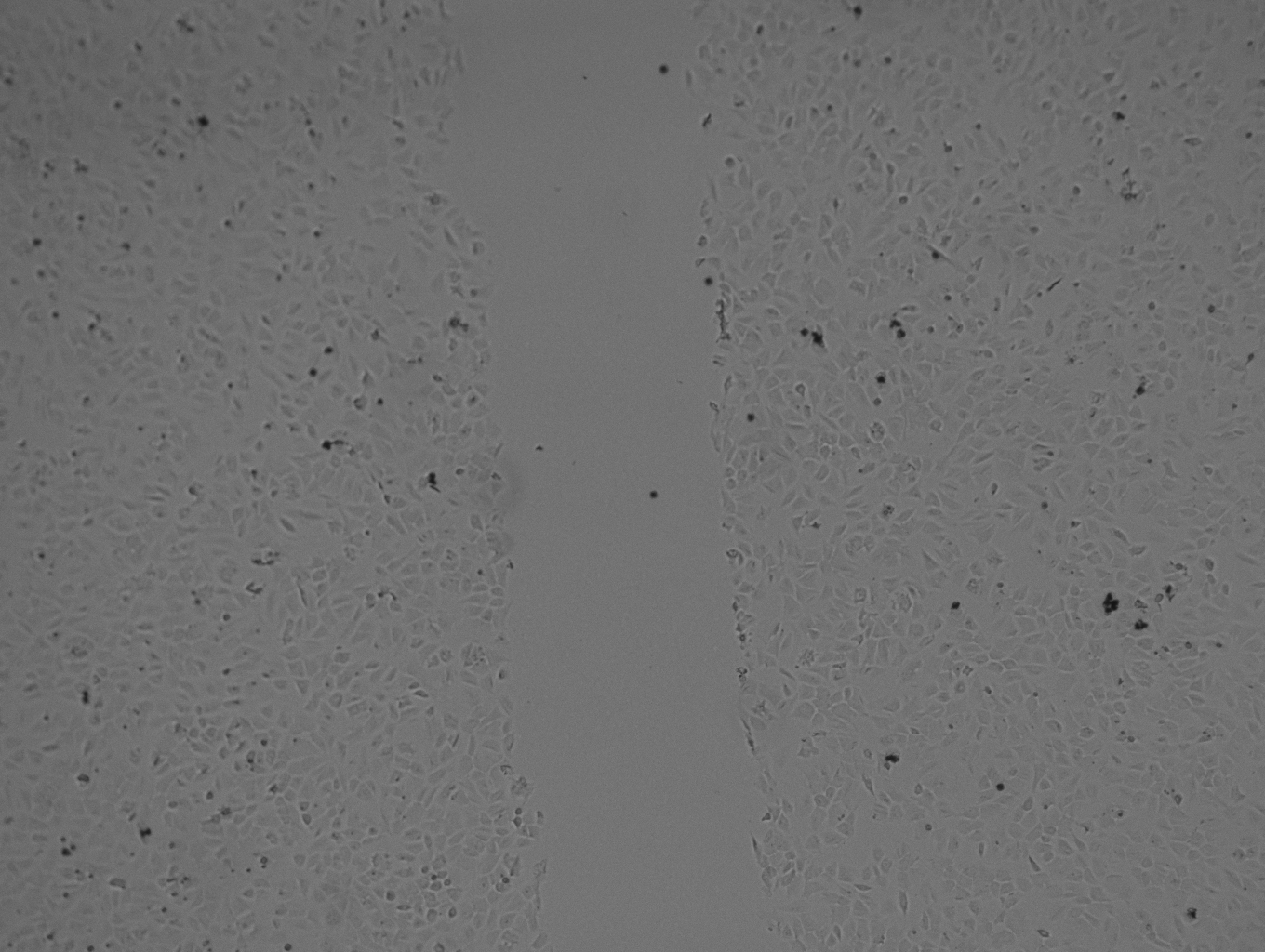

Supplement: Supplementary file 9 [file Data_Sheet_5.ZIP › Figure5 A 0h Co-culture 3.tif]

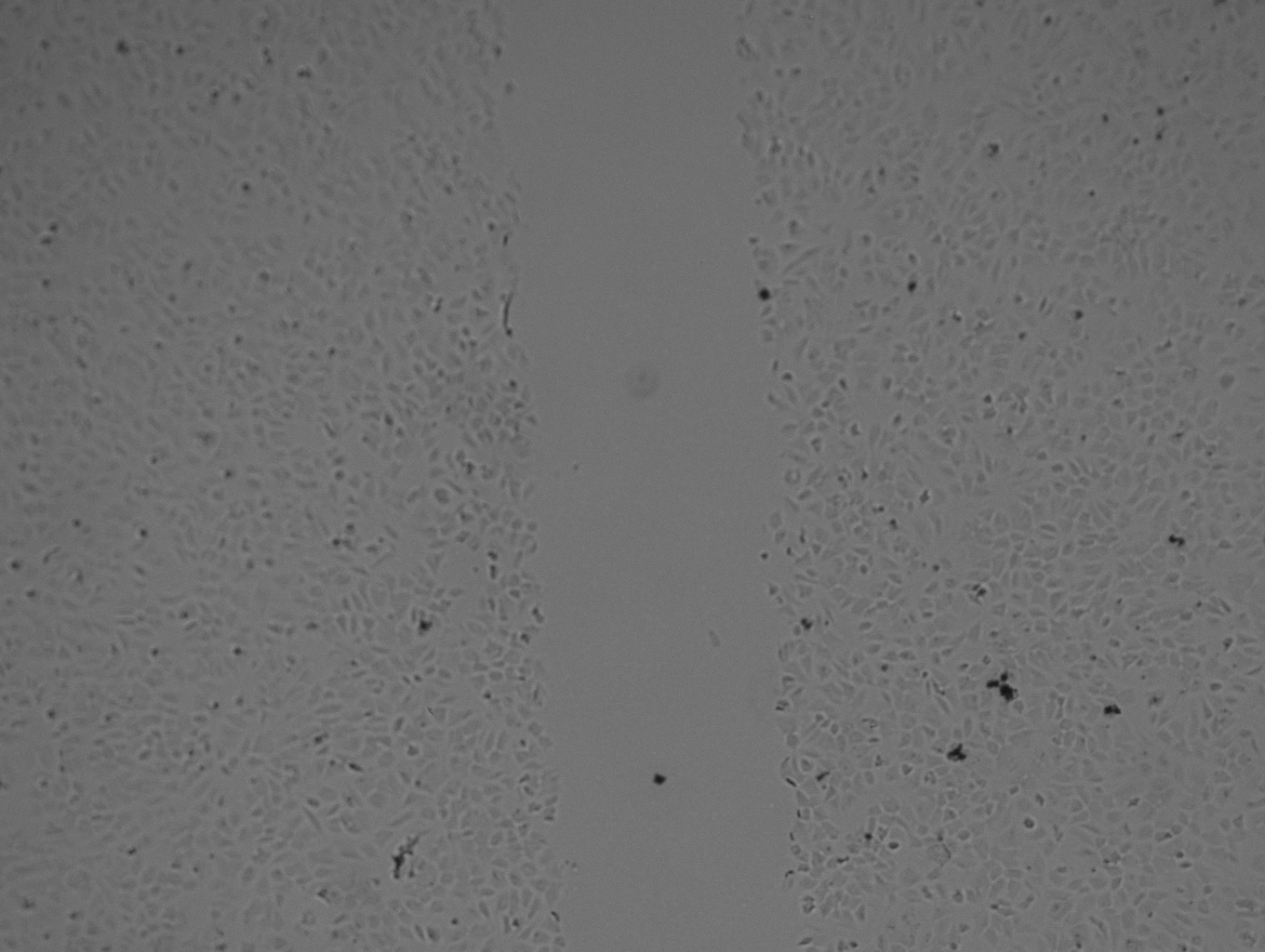

Supplement: Supplementary file 9 [file Data_Sheet_5.ZIP › Figure5 A 0h Co-culture 4.tif]

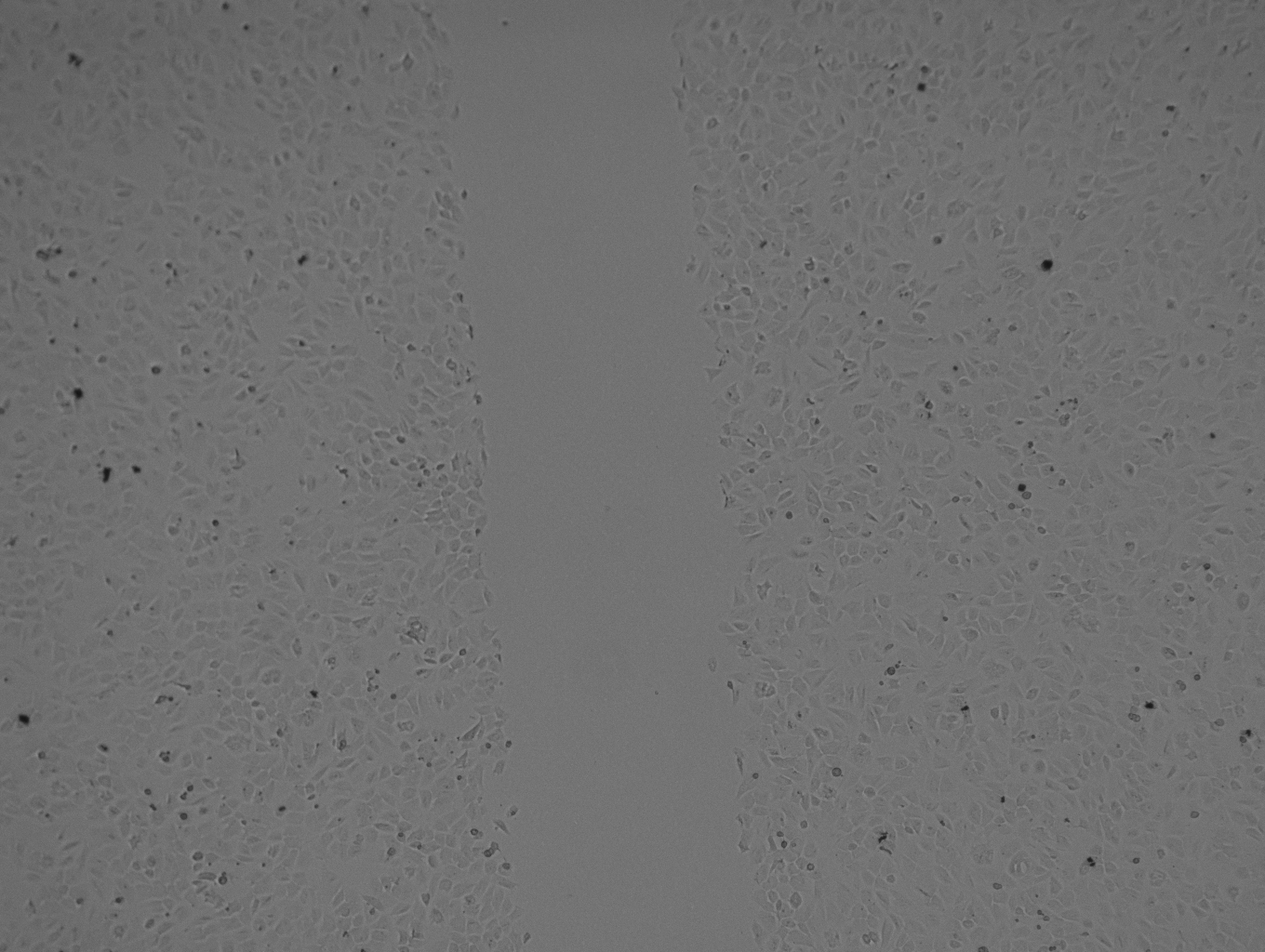

Supplement: Supplementary file 9 [file Data_Sheet_5.ZIP › Figure5 A 0h Co-culture.tif]

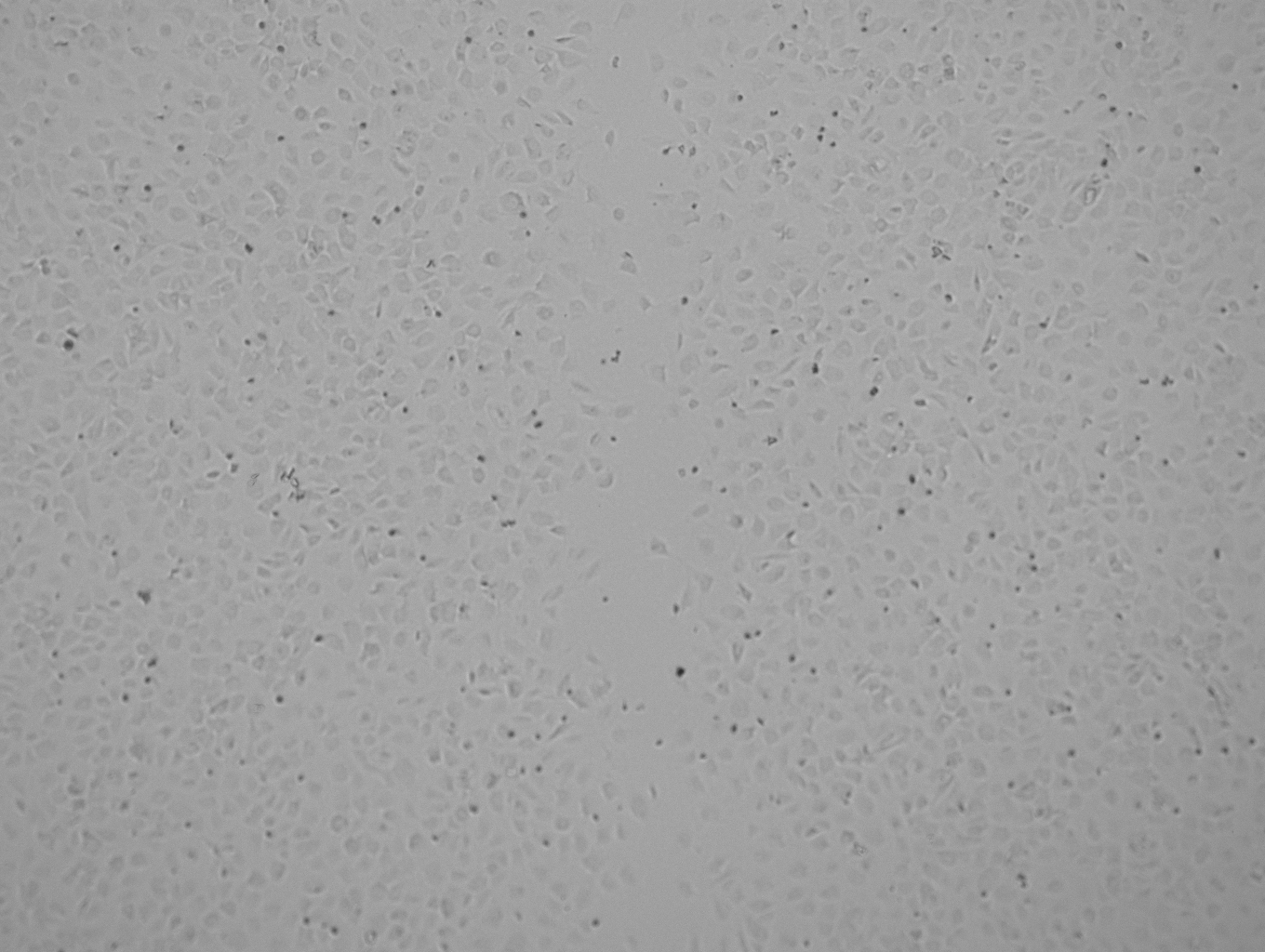

Supplement: Supplementary file 9 [file Data_Sheet_5.ZIP › Figure5 A Ad-ADAMTS8-Co 24h.tif]

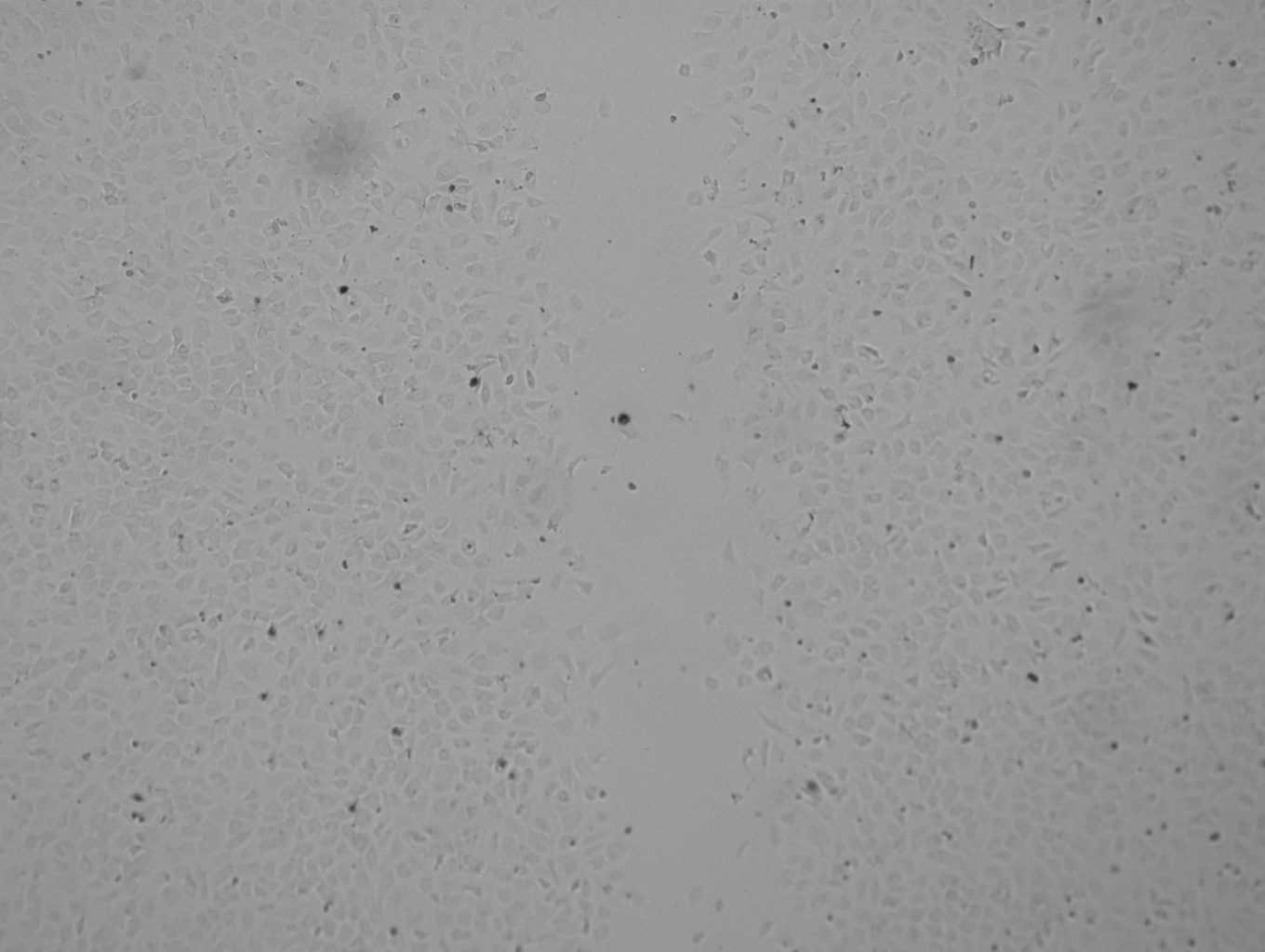

Supplement: Supplementary file 9 [file Data_Sheet_5.ZIP › Figure5 A Ad-v-Co 24h.tif]

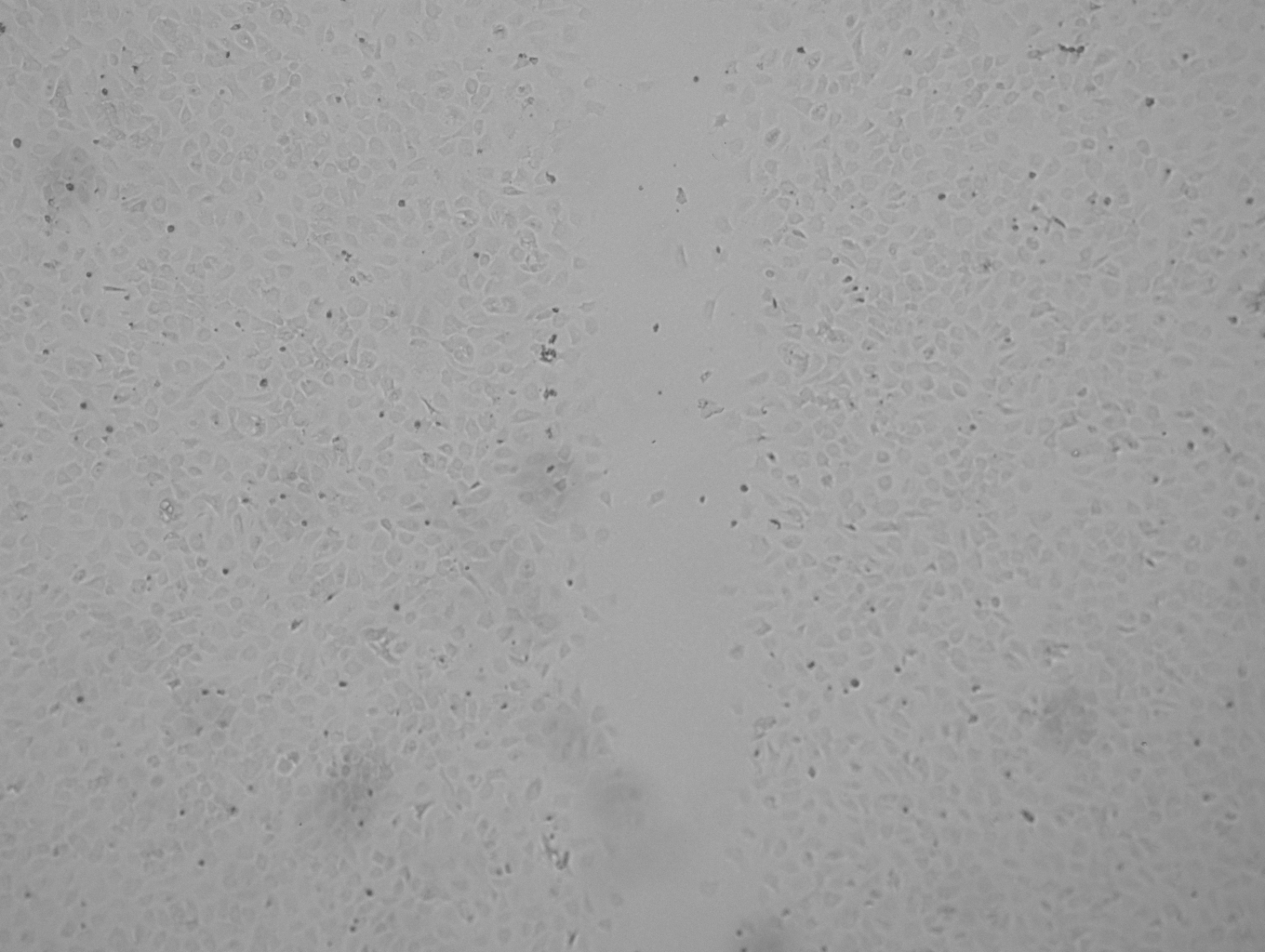

Supplement: Supplementary file 9 [file Data_Sheet_5.ZIP › Figure5 A SCramble-Co 24h.tif]

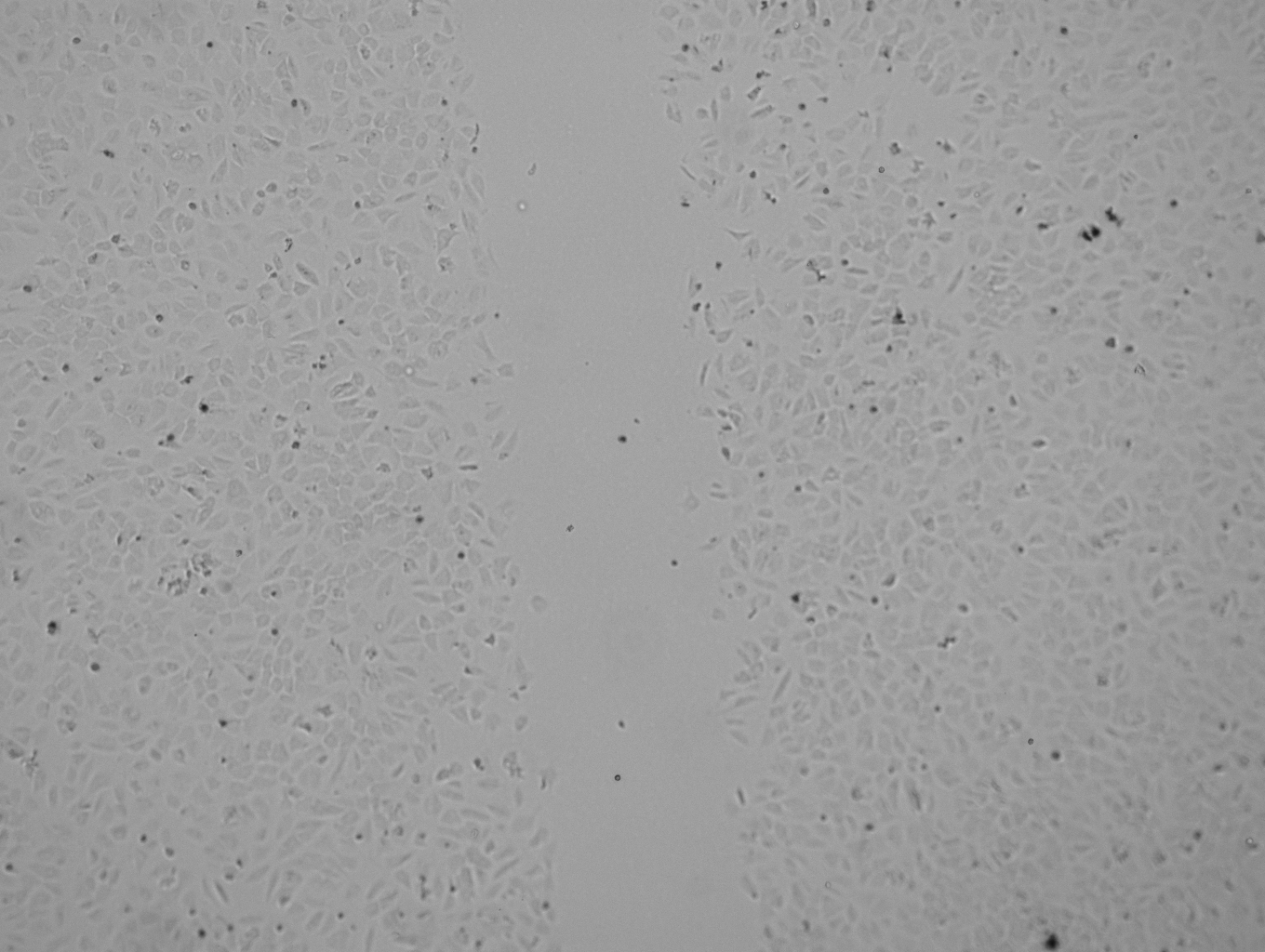

Supplement: Supplementary file 9 [file Data_Sheet_5.ZIP › Figure5 A si-ADAMTS8-Co 24h.tif]

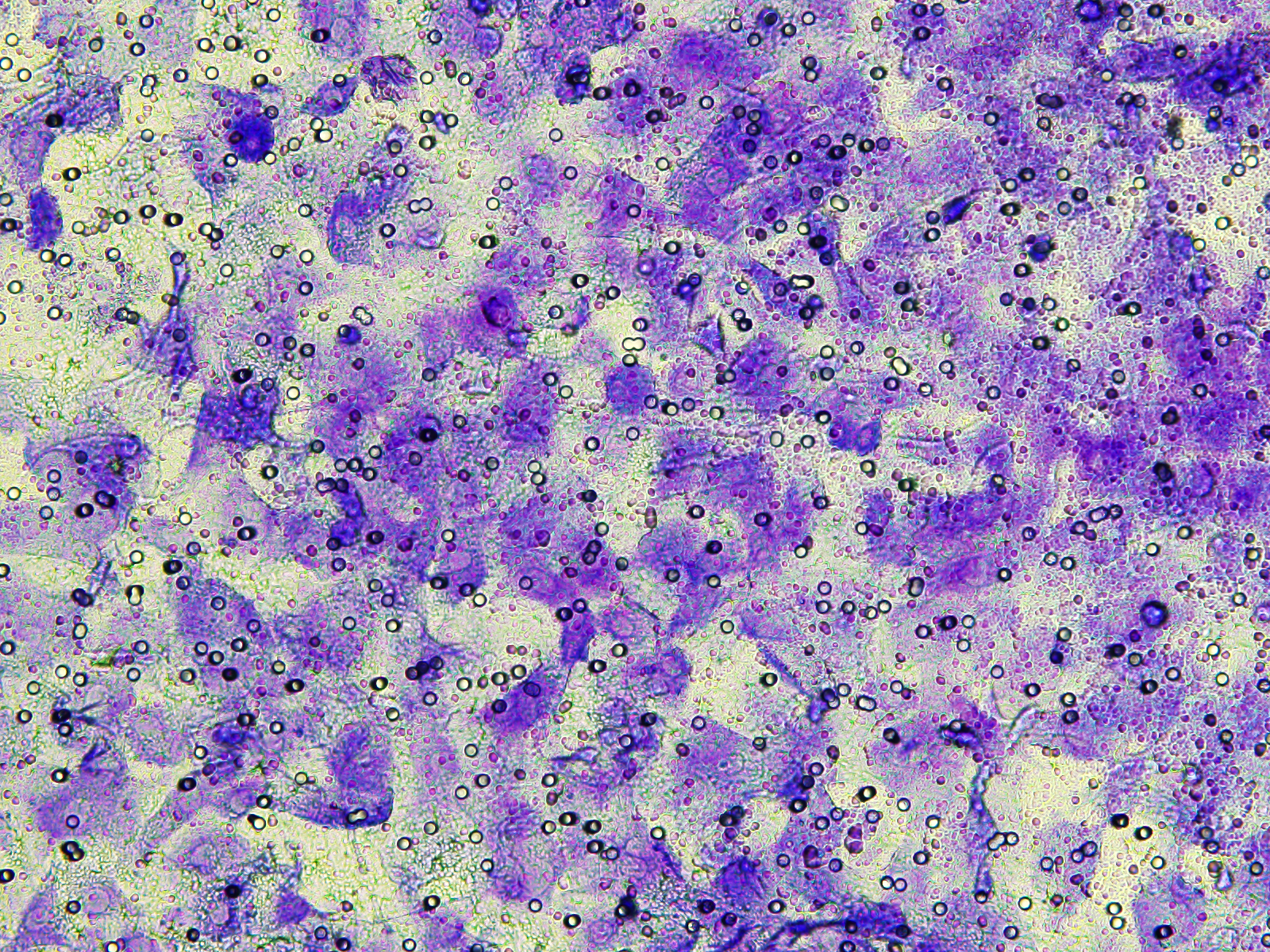

Supplement: Supplementary file 9 [file Data_Sheet_5.ZIP › Figure5 B Ad-ADAMTS8-Co.tif]

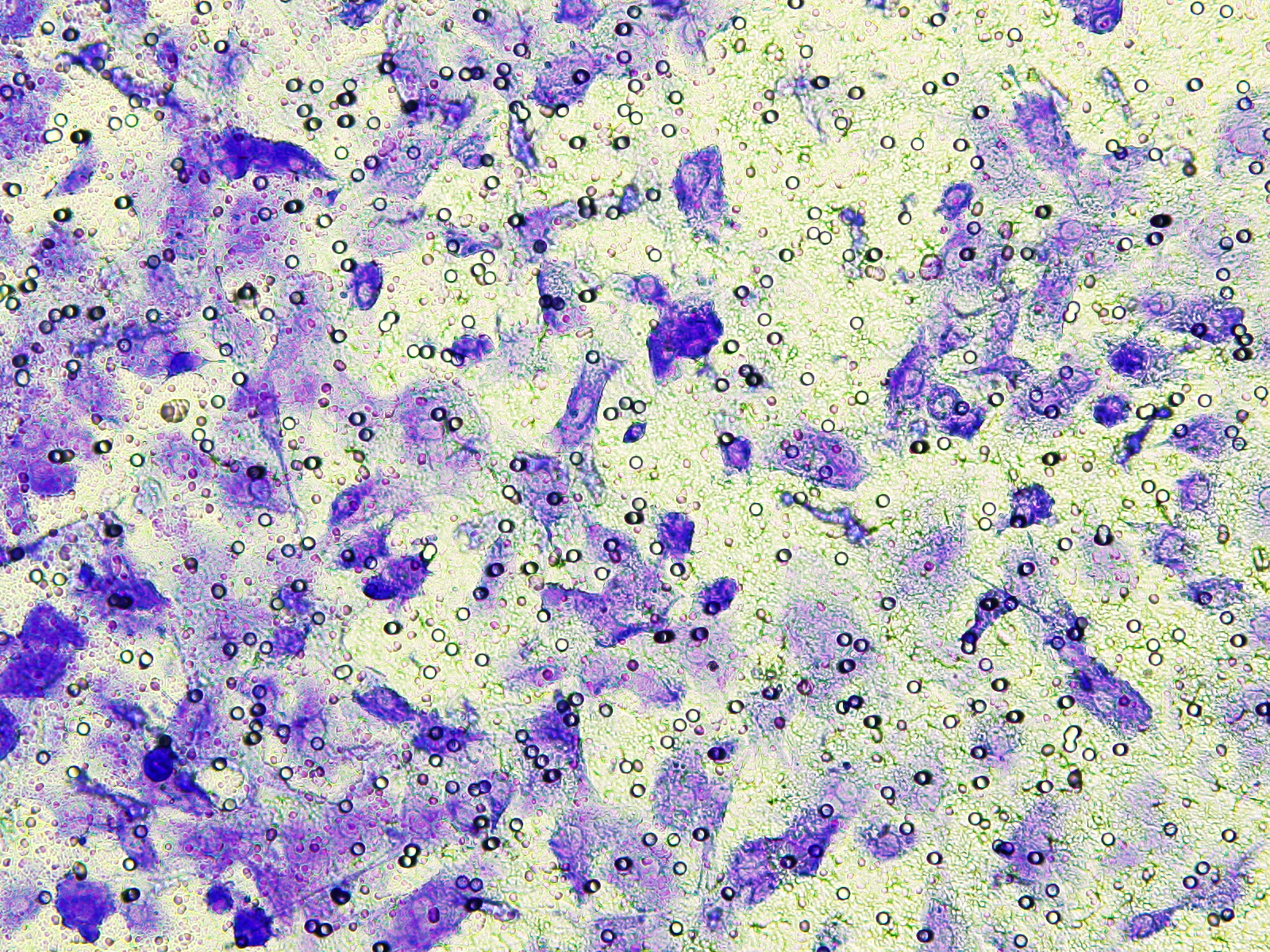

Supplement: Supplementary file 9 [file Data_Sheet_5.ZIP › Figure5 B Ad-v-co.tif]

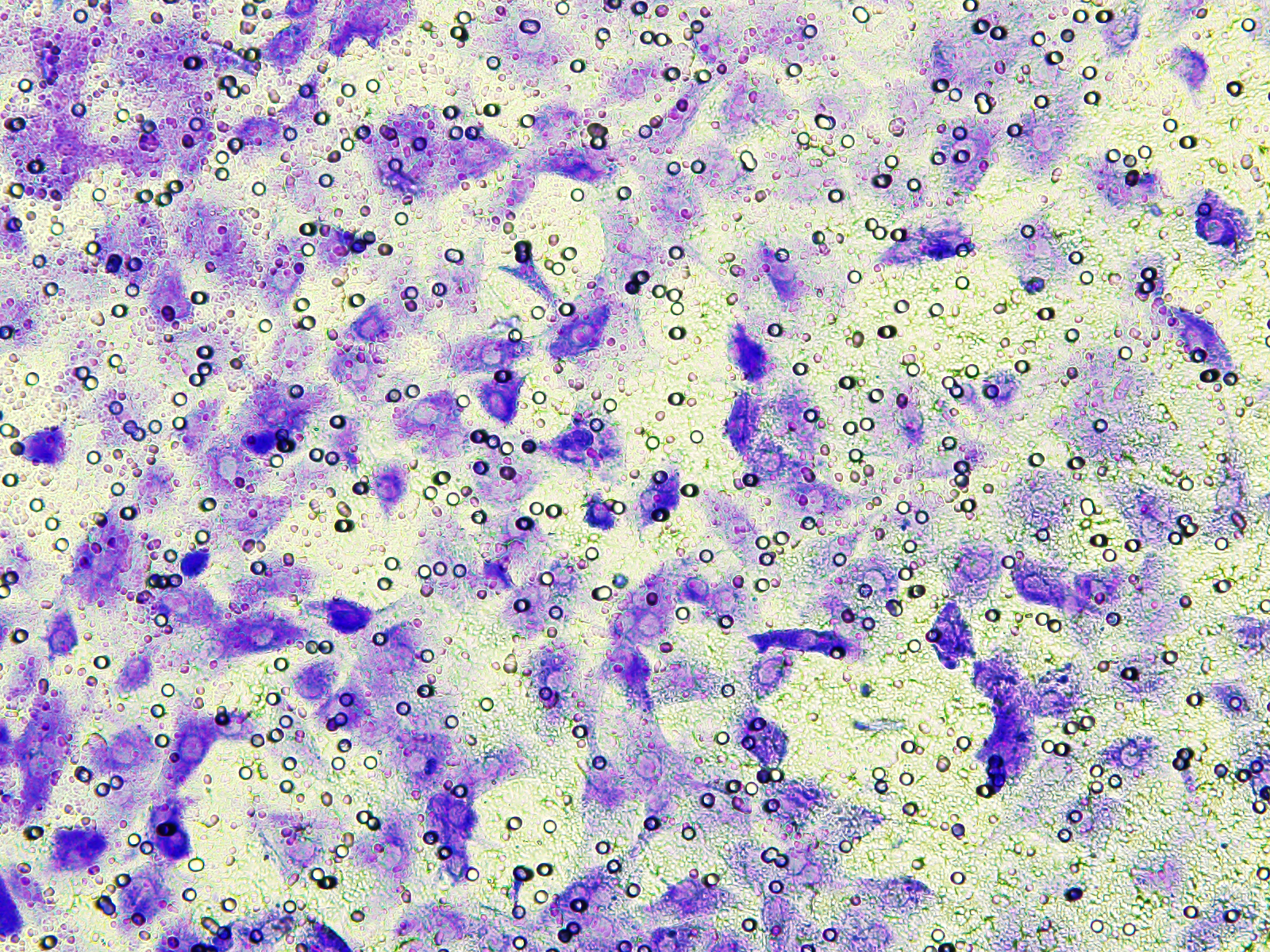

Supplement: Supplementary file 9 [file Data_Sheet_5.ZIP › Figure5 B SCR-Co.tif]

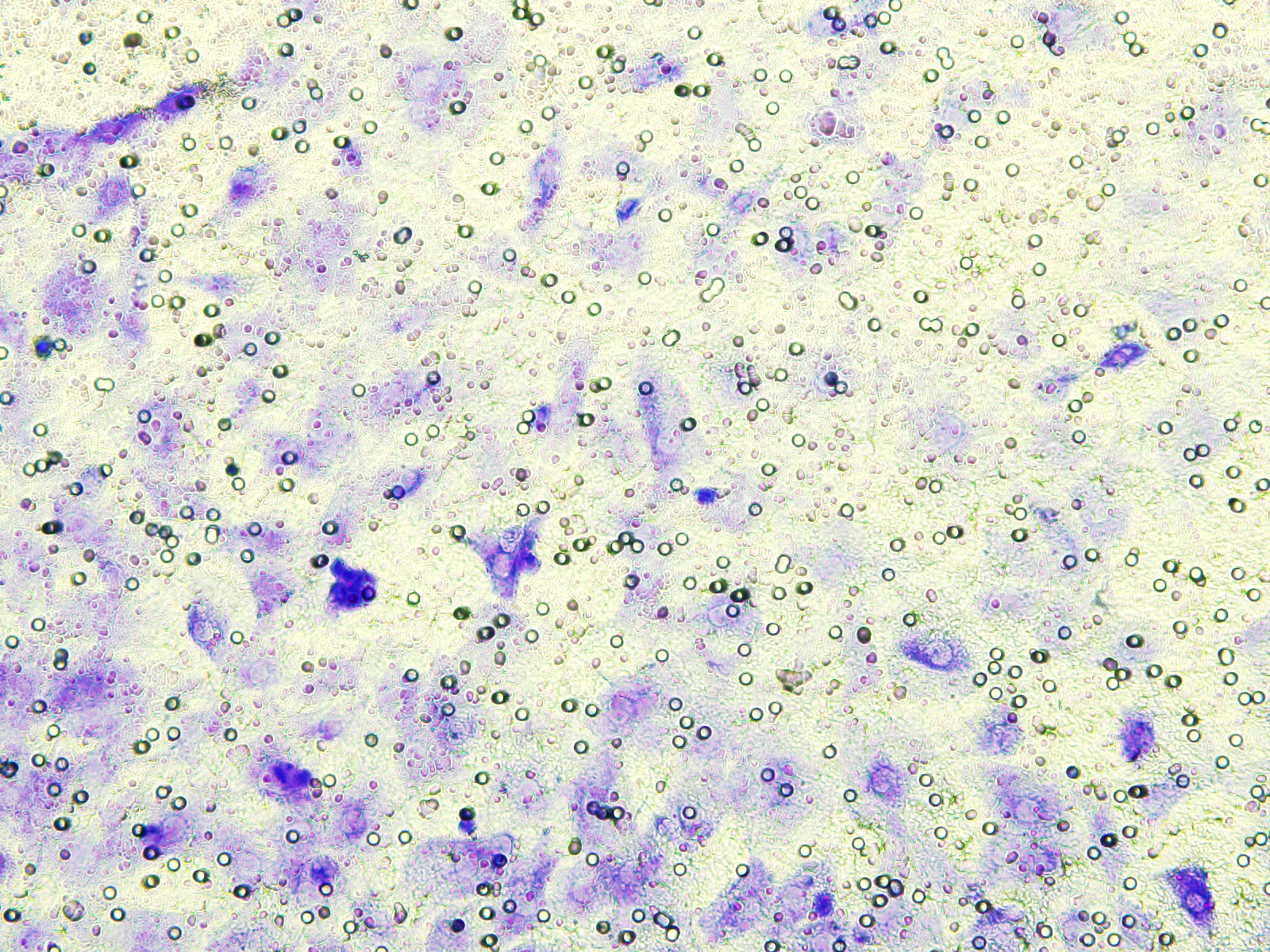

Supplement: Supplementary file 9 [file Data_Sheet_5.ZIP › Figure5 B si-ADAMTS8-Co.tif]

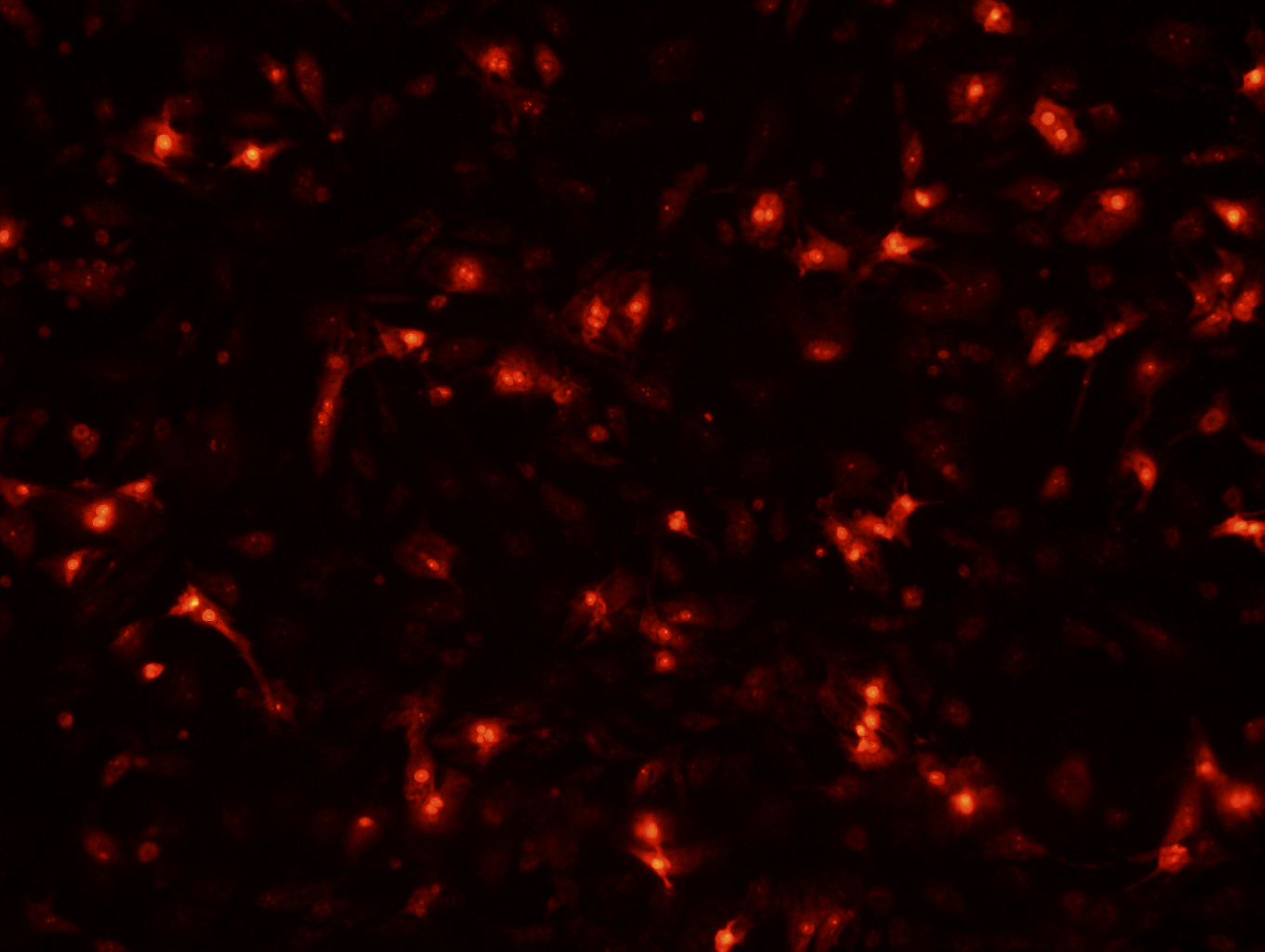

Supplement: Supplementary file 10 [file Data_Sheet_6.ZIP › Figure6 E Ad-ADAMTS8.tif]

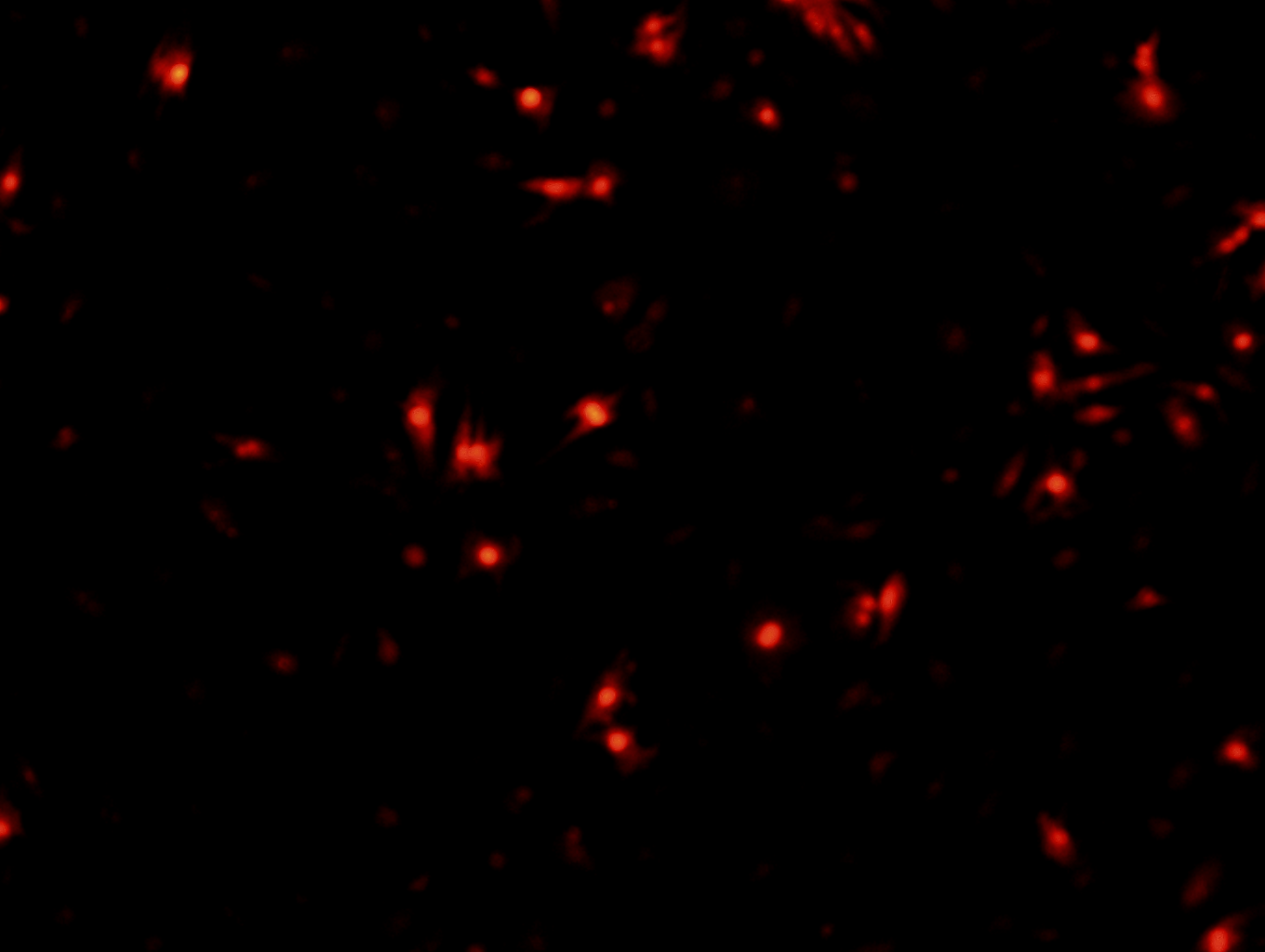

Supplement: Supplementary file 10 [file Data_Sheet_6.ZIP › Figure6 E Ad-v.tif]

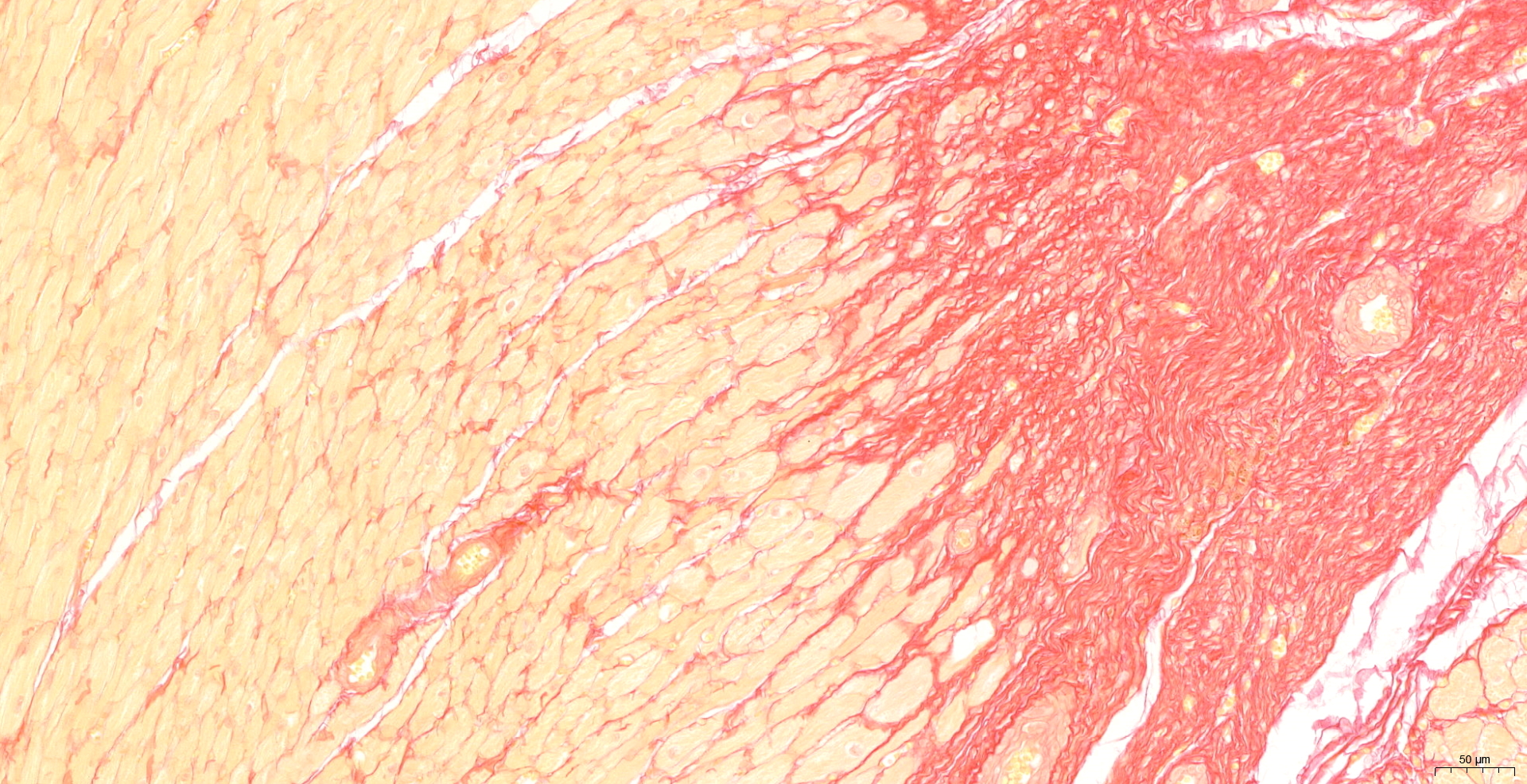

Supplement: Supplementary file 11 [file Data_Sheet_7.ZIP › Figure8 A MI Ad-ADAMTS8.jpg]

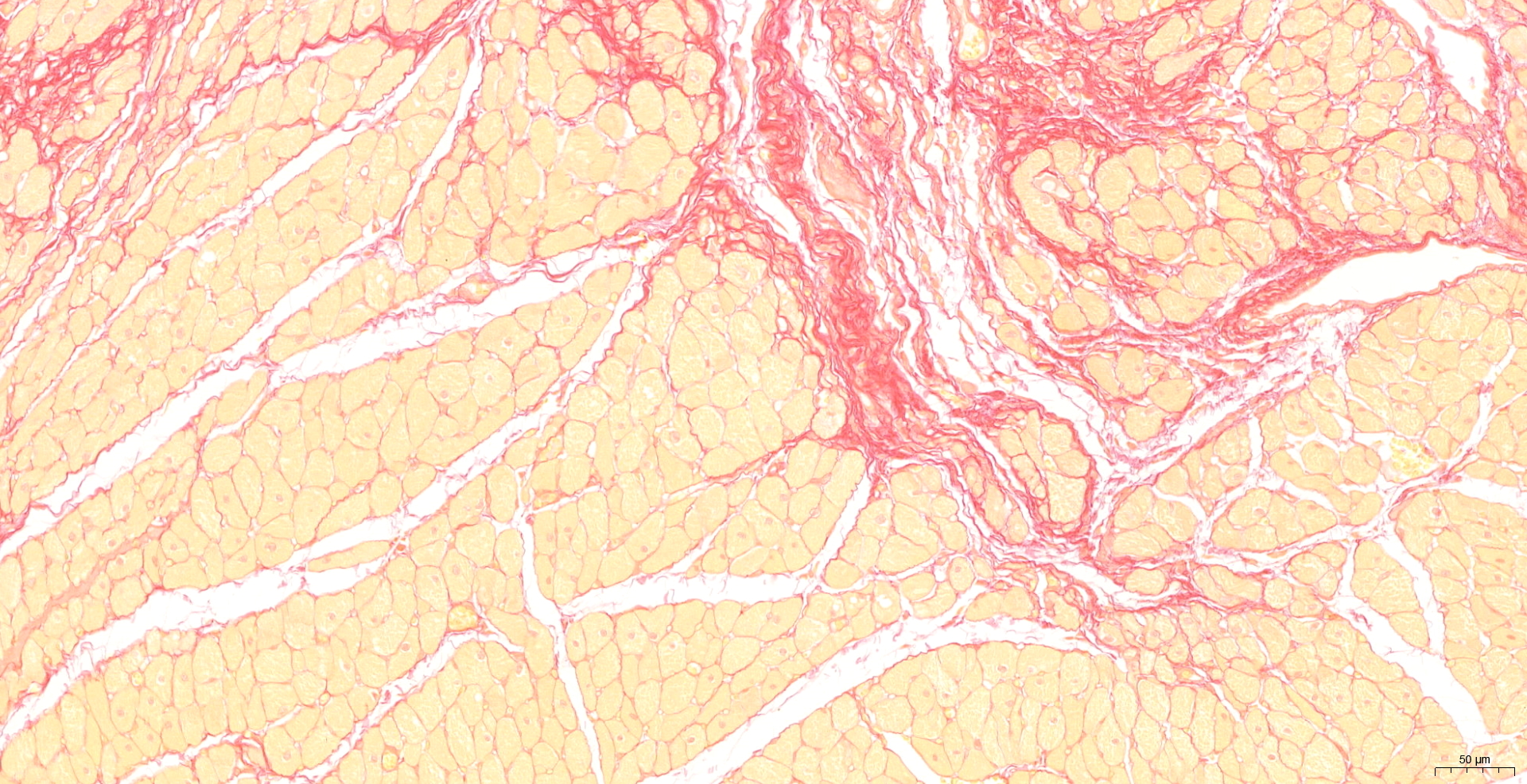

Supplement: Supplementary file 11 [file Data_Sheet_7.ZIP › Figure8 A MI Ad-v.jpg]

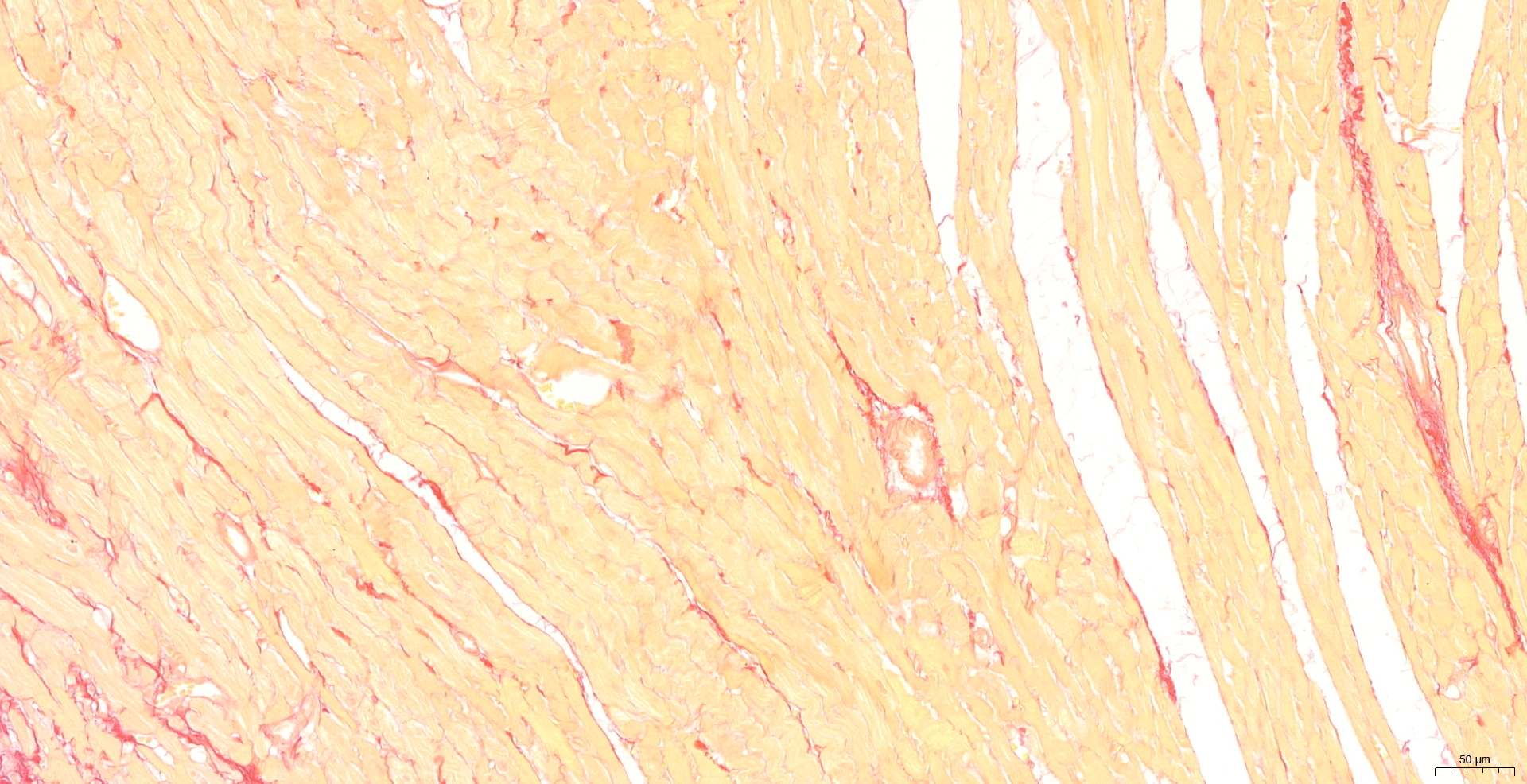

Supplement: Supplementary file 11 [file Data_Sheet_7.ZIP › Figure8 A Sham Ad-ADAMTS8.jpg]

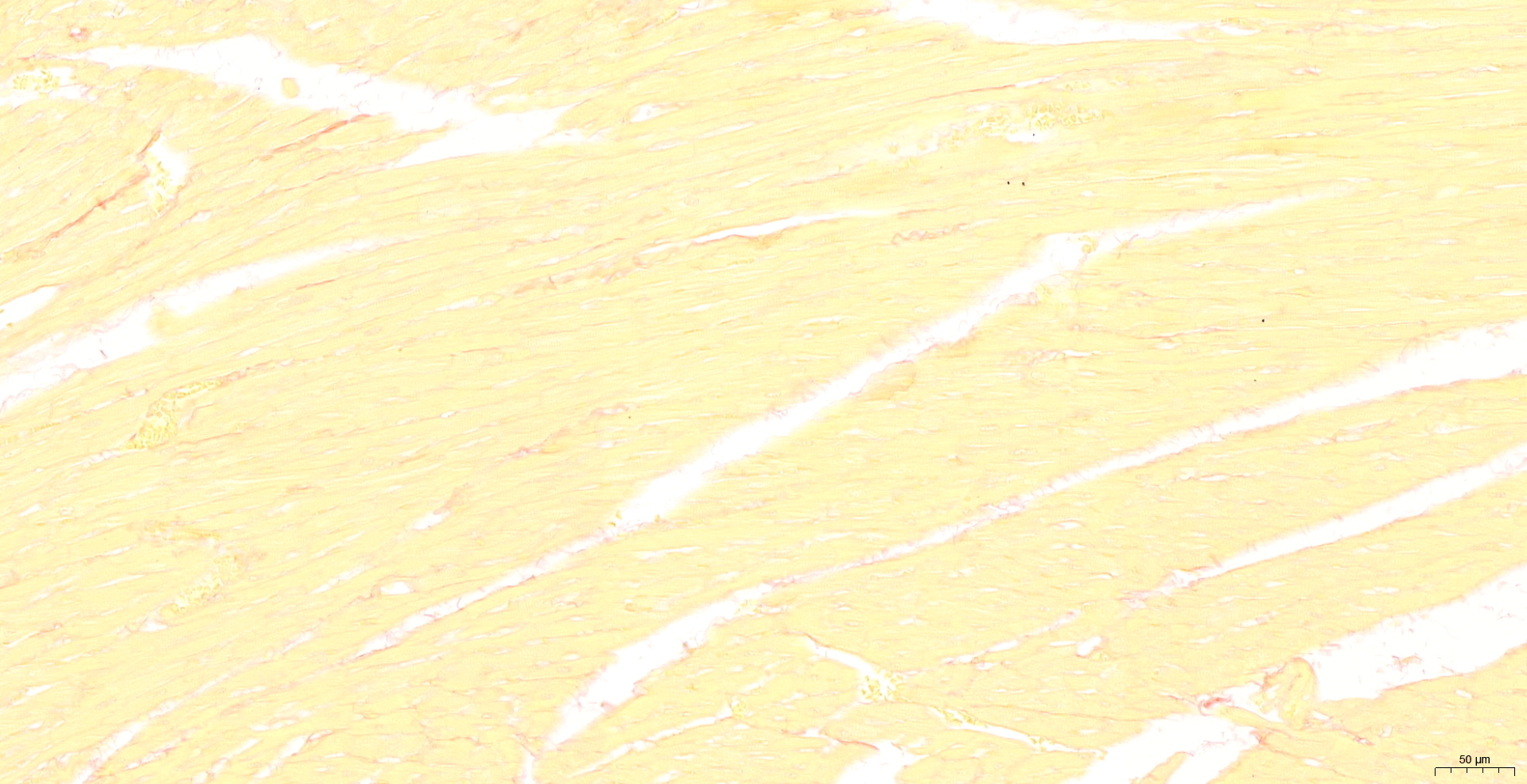

Supplement: Supplementary file 11 [file Data_Sheet_7.ZIP › Figure8 A Sham AD-v.jpg]

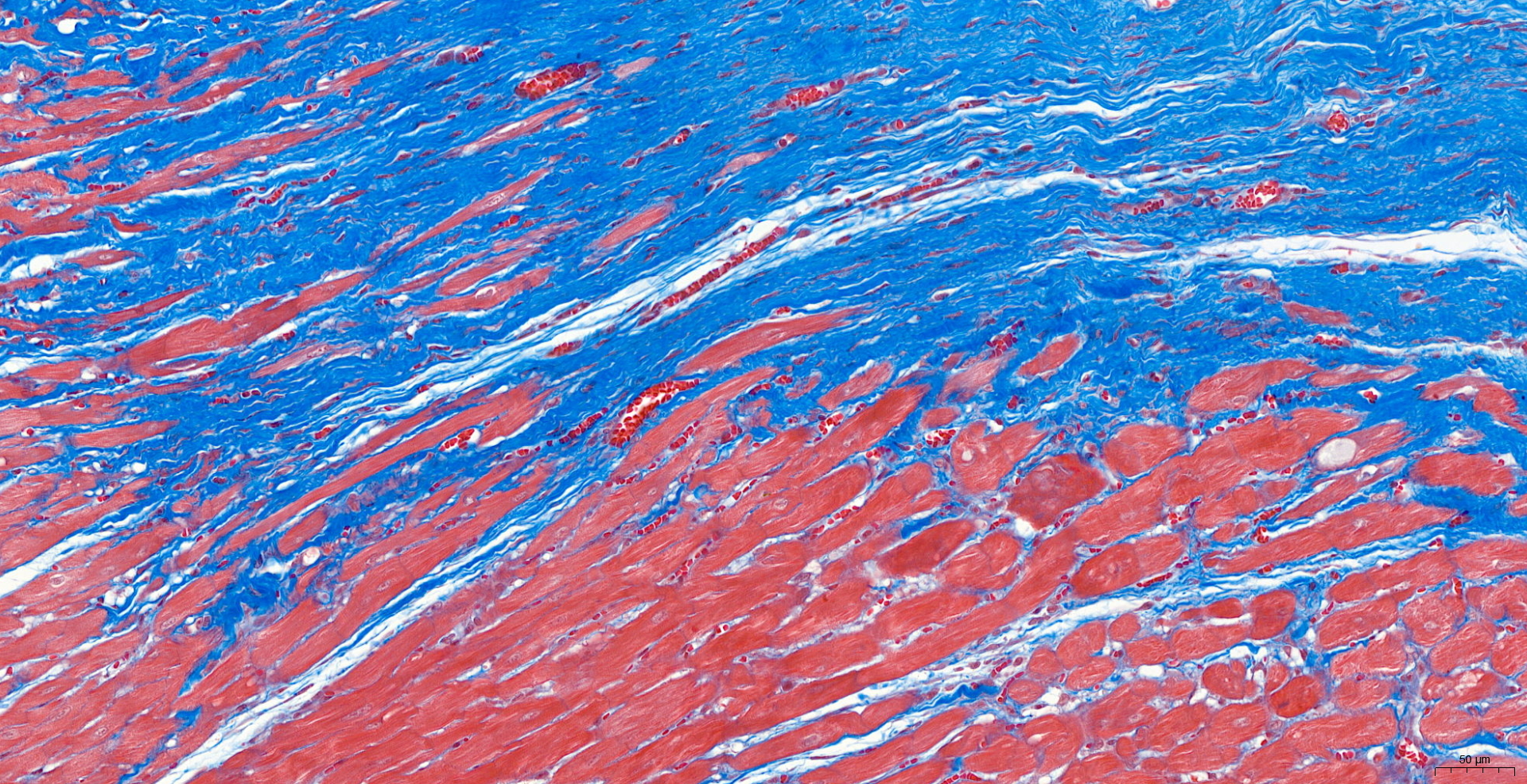

Supplement: Supplementary file 11 [file Data_Sheet_7.ZIP › Figure8 B MI Ad-ADAMTS8.jpg]

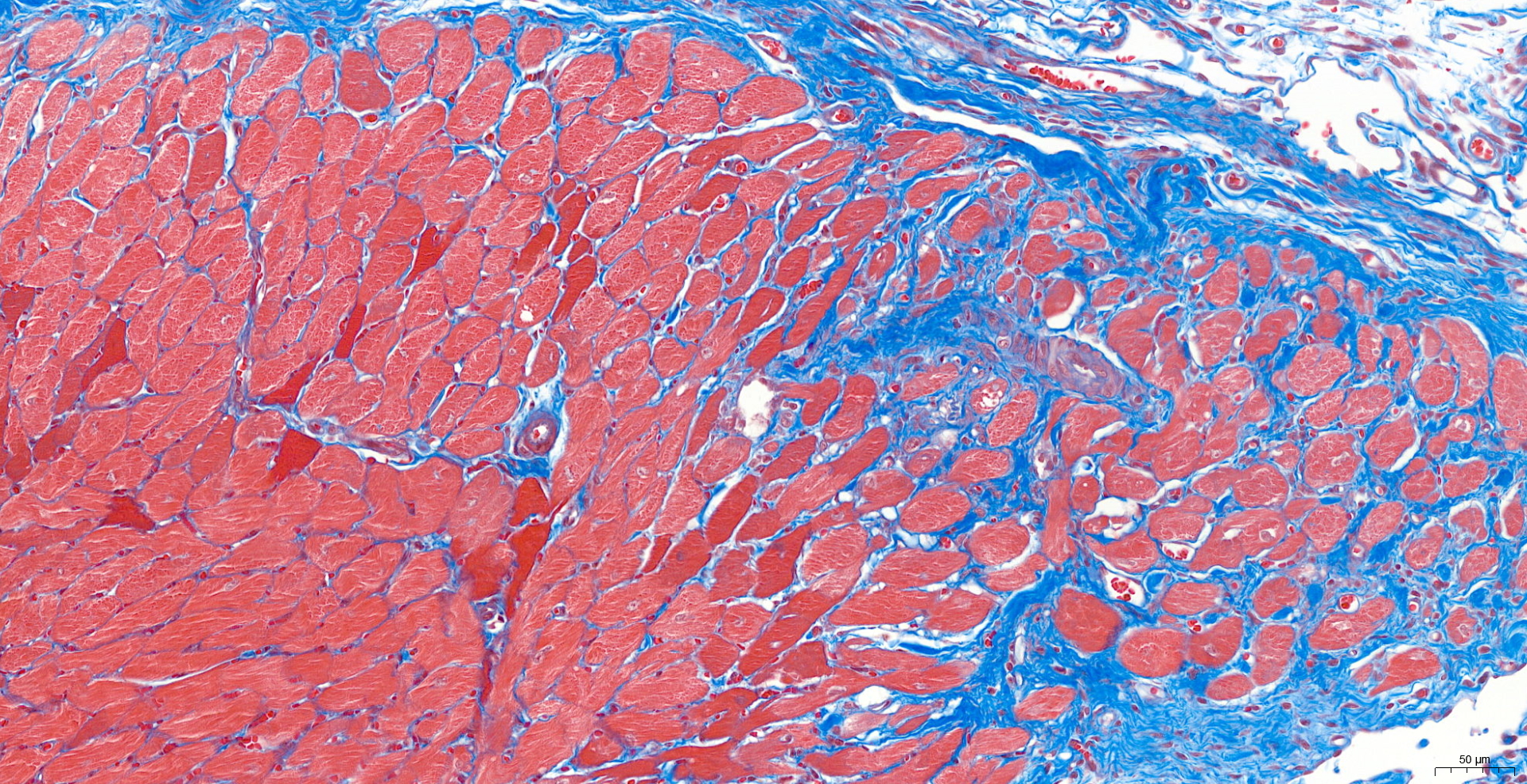

Supplement: Supplementary file 11 [file Data_Sheet_7.ZIP › Figure8 B MI Ad-v.jpg]

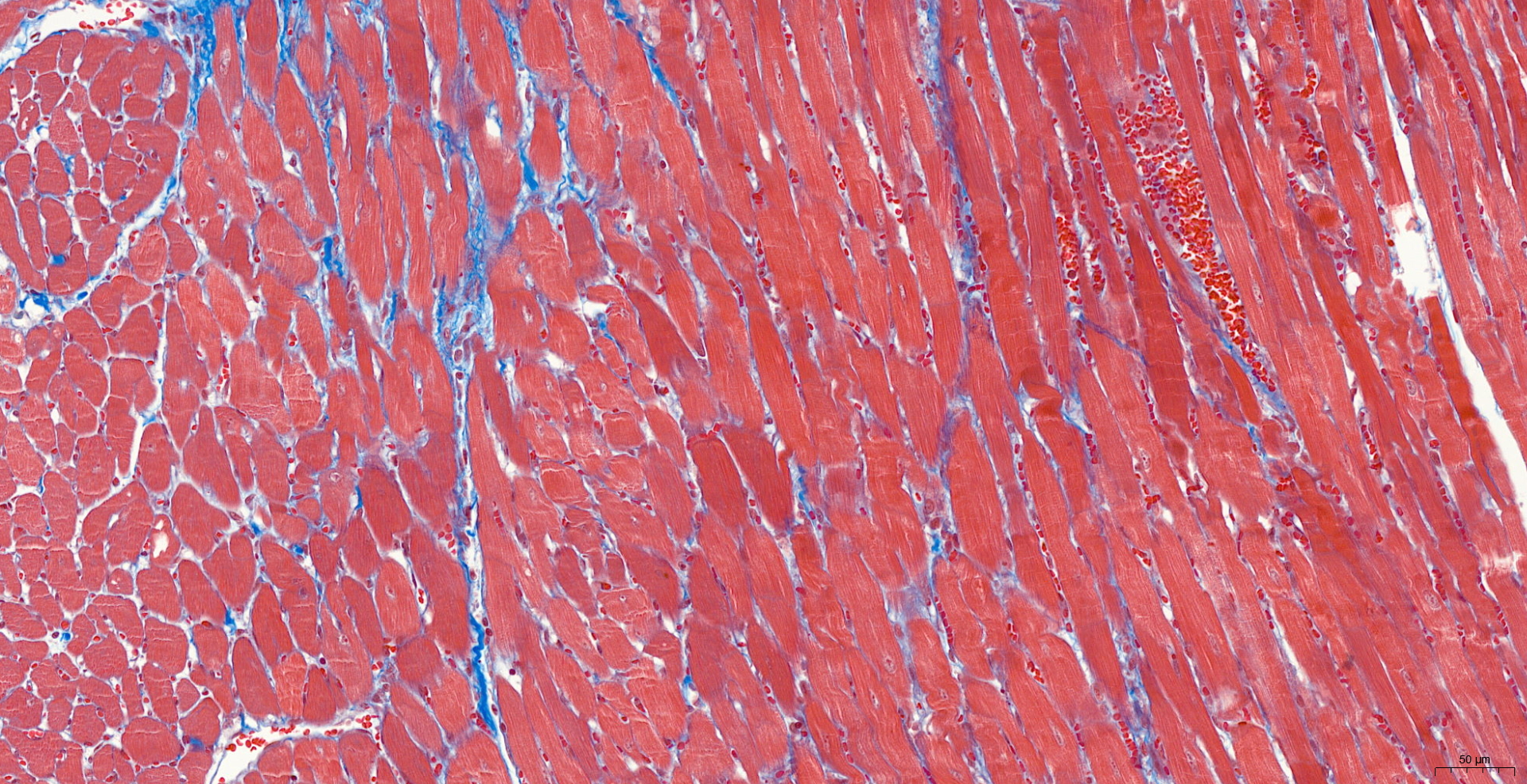

Supplement: Supplementary file 11 [file Data_Sheet_7.ZIP › Figure8 B Sham Ad-ADAMTS8.jpg]

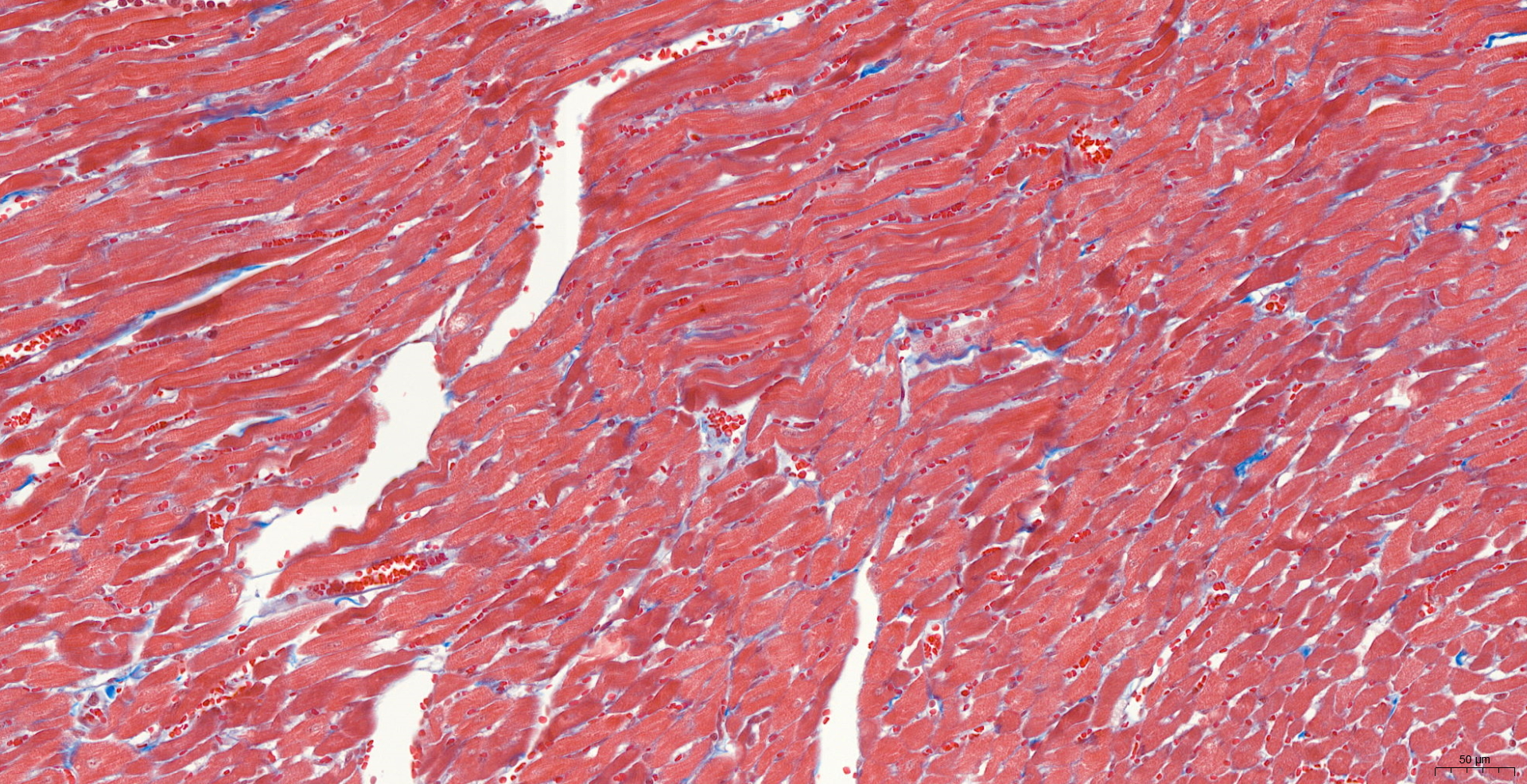

Supplement: Supplementary file 11 [file Data_Sheet_7.ZIP › Figure8 B Sham AD-v.jpg]
